# Mindfulness-Oriented Recovery Enhancement vs Supportive Group Therapy for Co-occurring Opioid Misuse and Chronic Pain in Primary Care: A Randomized Clinical Trial

**DOI:** 10.1001/jamainternmed.2022.0033

**Published:** 2022-02-28

**Authors:** Eric L. Garland, Adam W. Hanley, Yoshio Nakamura, John W. Barrett, Anne K. Baker, Sarah E. Reese, Michael R. Riquino, Brett Froeliger, Gary W. Donaldson

**Affiliations:** 1 Center on Mindfulness and Integrative Health Intervention Development, College of Social Work, University of Utah, Salt Lake City; 2 Veterans Health Care Administration, Veterans Integrated Service Network 19 Whole Health Flagship Site, VA Salt Lake City Health Care System, Salt Lake City, Utah; 3 Pain Research Center, Division of Pain Medicine, Department of Anesthesiology, University of Utah School of Medicine, Salt Lake City; 4 Community Physicians Group, University of Utah School of Medicine, Salt Lake City; 5 Department of Anesthesiology, Duke University, Durham, North Carolina; 6 School of Social Work, University of Montana, Missoula; 7 School of Social Welfare, University of Kansas, Lawrence; 8 Department of Psychology, University of Missouri, Columbia

## Abstract

**Importance:**

Successful treatment of opioid misuse among people with chronic pain has proven elusive. Guidelines recommend nonopioid therapies, but the efficacy of mindfulness-based interventions for opioid misuse is uncertain.

**Objective:**

To evaluate the efficacy of Mindfulness-Oriented Recovery Enhancement (MORE) for the reduction of opioid misuse and chronic pain.

**Design, Setting, and Participants:**

This interviewer-blinded randomized clinical trial enrolled patients from primary care clinics in Utah between January 4, 2016, and January 16, 2020. The study included 250 adults with chronic pain receiving long-term opioid therapy who were misusing opioid medications.

**Interventions:**

Treatment with MORE (comprising training in mindfulness, reappraisal, and savoring positive experiences) or supportive group psychotherapy (control condition) across 8 weekly 2-hour group sessions.

**Main Outcomes and Measures:**

Primary outcomes were (1) opioid misuse assessed by the Drug Misuse Index (self-report, interview, and urine screen) and (2) pain severity and pain-related functional interference, assessed by subscale scores on the Brief Pain Inventory through 9 months of follow-up. Secondary outcomes were opioid dose, emotional distress, and ecological momentary assessments of opioid craving. The minimum intervention dose was defined as 4 or more completed sessions of MORE or supportive group psychotherapy.

**Results:**

Among 250 participants (159 women [63.6%]; mean [SD] age, 51.8 [11.9] years), 129 were randomized to the MORE group and 121 to the supportive psychotherapy group. Overall, 17 participants (6.8%) were Hispanic or Latino, 218 (87.2%) were White, and 15 (6.0%) were of other races and/or ethnicities (2 American Indian, 3 Asian, 1 Black, 2 Pacific Islander, and 7 did not specify). At baseline, the mean duration of pain was 14.7 years (range, 1-60 years), and the mean (SD) morphine-equivalent opioid dose was 101.0 (266.3) mg (IQR, 16.0-90.0 mg). A total of 203 participants (81.2%) received the minimum intervention dose (mean [SD], 5.7 [2.2] sessions); at 9 months, 92 of 250 participants (36.8%) discontinued the study. The overall odds ratio for reduction in opioid misuse through the 9-month follow-up period in the MORE group compared with the supportive psychotherapy group was 2.06 (95% CI, 1.17-3.61; *P* = .01). At 9 months, 36 of 80 participants (45.0%) in the MORE group were no longer misusing opioids compared with 19 of 78 participants (24.4%) in the supportive psychotherapy group. Mixed models demonstrated that MORE was superior to supportive psychotherapy through 9 months of follow-up for pain severity (between-group effect: 0.49; 95% CI, 0.17-0.81; *P* = .003) and pain-related functional interference (between-group effect: 1.07; 95% CI, 0.64-1.50; *P* < .001). Participants in the MORE group reduced their opioid dose to a greater extent than those in the supportive psychotherapy group. The MORE group also had lower emotional distress and opioid craving.

**Conclusions and Relevance:**

In this randomized clinical trial, among adult participants in a primary care setting, the MORE intervention led to sustained improvements in opioid misuse and chronic pain symptoms and reductions in opioid dosing, emotional distress, and opioid craving compared with supportive group psychotherapy. Despite attrition caused by the COVID-19 pandemic and the vulnerability of the sample, MORE appeared to be efficacious for reducing opioid misuse among adults with chronic pain.

**Trial Registration:**

ClinicalTrials.gov Identifier: NCT02602535

## Key Points

**Question:**

Does a mindfulness-based intervention reduce comorbid chronic pain and opioid misuse in the primary care setting more than supportive psychotherapy?

**Findings:**

In this randomized clinical trial that included 250 adults with both chronic pain and opioid misuse, 45.0% of participants receiving Mindfulness-Oriented Recovery Enhancement (MORE) were no longer misusing opioids after 9 months of follow-up compared with 24.4% of participants receiving supportive group psychotherapy. Participants receiving MORE also reported significant improvements in chronic pain symptoms compared with those receiving supportive psychotherapy.

**Meaning:**

In this study, MORE appeared to be an efficacious treatment for opioid misuse among adults with chronic pain.

## Teaser

This randomized clinical trial assesses whether Mindfulness-Oriented Recovery Enhancement is more efficacious than supportive group psychotherapy for reducing opioid misuse among adults with chronic pain receiving long-term opioid therapy.

## Introduction

Long-term opioid therapy (LTOT; ≥90 days of opioid use^1^) for the treatment of chronic pain is common in primary care settings,^2^ despite risks including opioid misuse and opioid use disorder (OUD). Approximately 25% of individuals receiving LTOT misuse opioids,^3^ which is defined as aberrant drug-related behaviors^4^ (eg, use of opioids to alleviate negative emotions) inconsistent with prescription directions.^5^ Escalation from chronic pain to opioid misuse and OUD is thought to be propelled by effects of prolonged opioid use on stress and reward circuitry in the brain.^6^ These neurobiological changes increase sensitization to emotional distress and pain and decrease sensitivity to pleasure derived from natural rewards,^6,7^ promoting opioid dose escalation as a means of preserving a dwindling sense of well-being.^8^ Because of these complex pathogenic mechanisms as well as a dearth of studies in this area, successful treatment of opioid misuse among people receiving LTOT has proven elusive. Reviews^9,10^ have identified a lack of full-scale interventional clinical trials examining approaches to mitigate the risk of high-dose opioid use and misuse among people with chronic pain. With few exceptions,^11,12^ this evidence gap remains.

To combat the opioid crisis, guidelines from the Centers for Disease Control and Prevention^9^ have encouraged clinicians to consider nonpharmacologic therapies, such as mindfulness. Yet, absent large clinical trials, the impact of mindfulness techniques on patients with co-occurring pain and opioid misuse is uncertain.^13^ Mindfulness-Oriented Recovery Enhancement (MORE)^14^ unites mindfulness training, cognitive behavioral therapy (CBT), and principles from positive psychology into an integrative group therapy to target the reward dysregulation underpinning chronic pain and opioid misuse.^15^ Given preliminary evidence of the efficacy of MORE in pilot studies with short follow-up periods,^16,17,18^ this full-scale clinical trial with 9 months of follow-up evaluated the efficacy of MORE delivered in a primary care setting compared with a supportive group psychotherapy control condition (validated in previous studies^16,17,19^) designed to control for nonspecific therapeutic factors (eg, social support and therapeutic relationships).^20^ We hypothesized that implementation of the MORE intervention among adults with both chronic pain and opioid misuse who were receiving LTOT would result in significant reductions in opioid misuse, pain severity, and pain-related functional interference (primary outcomes) as well as decreases in opioid dosing, emotional distress, and opioid craving (secondary outcomes) compared with supportive psychotherapy.

## Methods

### Design, Setting, and Participants

This study was a parallel superiority randomized clinical trial with blinded assessments. Participants were recruited from January 4, 2016, to January 16, 2020. The University of Utah Institutional Review Board approved the study protocol (Supplement 1). All participants provided written informed consent. This study followed the Consolidated Standards of Reporting Trials (CONSORT) reporting guideline for randomized clinical trials.

Participants were recruited from and received treatment in 6 University of Utah primary care clinics across the Salt Lake Valley. Eligible participants were 18 years or older with a physician-confirmed chronic pain–related diagnosis and prescribed daily opioid use for 3 or more months, an average pain rating of 3 or greater on a 0 to 10 numeric scale, and a score higher than the validated cutoff (≥9 points) for opioid misuse on the Current Opioid Misuse Measure (COMM).^5^ We excluded patients receiving cancer treatment; those with suicidal behavior, psychosis, and/or a severe nonopioid substance use disorder (as assessed by the Mini International Neuropsychiatric Interview)^21^; and/or those who had previous exposure to mindfulness interventions (eg, mindfulness-based stress reduction). Participants were recruited from electronic health record data, physician referrals, and community advertisements. After obtaining informed consent, study coordinators collected demographic information and outcomes (Table 1). Participants were compensated $320 for completing all study activities.

**Table 1.** Participant Demographic and Clinical Characteristics

Characteristic	No. (%)	
	MORE group	Supportive psychotherapy group
Total participants, No.	129	121
Age, mean (SD), y	50.9 (12.4)	52.8 (11.3)
Sex		
Female	81 (62.8)	78 (64.5)
Male	48 (37.2)	43 (35.5)
Race and ethnicity		
Hispanic or Latino	11 (8.5)	6 (5.0)
White	109 (84.5)	109 (90.0)
Other^a^	9 (7.0)	6 (5.0)
Highest level of education		
<High school	13 (10.1)	13 (10.7)
High school	60 (46.5)	58 (47.9)
≥College	54 (41.9)	49 (40.5)
Missing	2 (1.6)	1 (0.8)
Estimated household income, $		
<25 000	49 (38.0)	49 (40.5)
25 000-49 999	40 (31.0)	34 (28.1)
50 000-99 999	24 (18.6)	26 (21.5)
≥100 000	14 (10.9)	11 (9.1)
Pain condition and location^b^		
Back	87 (67.4)	84 (69.4)
Osteoarthritis	60 (46.5)	52 (43.0)
Fibromyalgia	32 (24.8)	32 (26.4)
Neuropathic	39 (30.2)	24 (19.8)
Cervical pain	29 (22.5)	33 (27.3)
Extremity pain	27 (20.9)	32 (26.4)
Migraine or tension headache	14 (10.9)	25 (20.7)
Irritable bowel syndrome	11 (8.5)	11 (9.1)
Interstitial cystitis/pelvic pain	5 (3.9)	5 (4.1)
Other	11 (8.5)	7 (5.8)
Pain, mean (SD)		
BPI severity^c^	5.6 (1.5)	5.2 (1.5)
BPI functional interference^c^	6.5 (1.9)	6.1 (2.1)
Duration, y	14.6 (11.1)	14.7 (10.1)
Opioid prescription^b^		
Oxycodone	47 (36.4)	49 (40.5)
Hydrocodone	39 (30.2)	38 (31.4)
Tramadol	21 (16.3)	25 (20.7)
Morphine	17 (13.2)	17 (14.0)
Buprenorphine	7 (5.4)	7 (5.8)
Methadone	8 (6.2)	5 (4.1)
Other	10 (7.8)	15 (12.4)
Duration of opioid use, mean (SD), y	9.2 (7.4)	9.6 (8.2)
MEDD, mg		
Mean (SD)	110.7 (356.4)	91.7 (132.3)
IQR	15.0-80.0	18.0-90.1
Opioid misuse and craving, mean (SD)		
COMM misuse^d^	17.2 (7.0)	18.4 (8.1)
EMA craving^e^	5.0 (3.4)	5.4 (3.7)
DASS emotional distress and depression, mean (SD)^f^		
Emotional distress	21.5 (11.6)	22.8 (10.6)
Depression	7.5 (5.2)	8.3 (5.0)
Antidepressant medication prescription	51 (39.5)	56 (46.3)
Opioid use disorder	75 (58.1)	82 (67.8)
Alcohol or nonopioid substance use disorder	23 (17.8)	31 (25.6)
Major depressive disorder	87 (67.4)	84 (69.4)
Generalized anxiety disorder	25 (19.4)	24 (19.8)
Posttraumatic stress disorder	21 (16.3)	16 (13.2)

Abbreviations: BPI, Brief Pain Inventory; COMM, Current Opioid Misuse Measure; DASS, Depression Anxiety Stress Scale; EMA, ecological momentary assessment; MEDD, morphine-equivalent daily dose; MORE, Mindfulness-Oriented Recovery Enhancement.

^a^ Two participants were American Indian, 3 were Asian, 1 was Black, 2 were Pacific Islander, and 7 did not specify.

^b^ Some percentages sum to greater than 100% because participants could report multiple pain conditions or locations and opioid prescriptions.

^c^ Score range, 0-10, with higher scores on the pain severity subscale indicating more severe pain and higher scores on the pain-related functional interference subscale indicating greater impairments in daily functioning.

^d^ Score range, 0-68, with higher scores indicating greater likelihood of current opioid misuse.

^e^ Score range, 0-10, with higher scores indicating more severe craving.

^f^ Score range, 0-63, with higher scores indicating more severe symptoms of depression, anxiety, and stress.

### Blinding and Randomization

A study coordinator uninvolved in assessments or analysis randomized participants to receive the MORE intervention or supportive psychotherapy using random assignment (1:1 ratio) in blocks of 2 to 4 people via computerized random number generator. To prevent bias and maintain randomization concealment, participants were not randomized by the study coordinator until the day of the first treatment session. Assessments were conducted by staff members who were blinded to randomization groups (which remained concealed throughout the study). To maintain blinding, the study key with randomization groups was inaccessible to staff members involved in assessment as well as the principal investigator (E.L.G.) and statistician (G.W.D.) until study completion. Before each assessment, participants were reminded not to disclose their treatment assignment to staff.

### Interventions

The MORE and supportive psychotherapy interventions were delivered in primary care clinics to groups of 6 to 12 participants across 8 weekly 2-hour sessions. The same set of clinical social workers (A.K.B., S.E.R., M.R.R., and another nonauthor clinician; mean [SD], 6.5 [2.6] years of experience) provided both MORE and supportive psychotherapy.

The manualized MORE intervention^14^ provided sequenced training in mindfulness, reappraisal, and savoring skills (eMethods 1 in Supplement 2). Mindfulness consisted of meditation on breathing and body sensations to strengthen self-regulation of compulsive opioid use and to mitigate pain and opioid craving by reinterpreting these experiences as innocuous sensory information. Reappraisal consisted of reframing maladaptive thoughts to decrease negative emotions and engender meaning in life. Savoring consisted of training in focusing awareness on pleasurable events and sensations to amplify positive emotions and reward. Sessions provided psychoeducation to address opioid misuse and chronic pain. Participants were asked to engage in daily 15-minute audio-guided mindfulness, reappraisal, and savoring practices and to log practice minutes daily via their smartphones. In addition, participants were instructed to practice 3 minutes of mindfulness before taking opioid medications to clarify whether opioid use was due to craving or the need for pain relief and to foster an opioid-sparing effect by providing a nonpharmacologic pain management approach. The minimum intervention dose of MORE (and supportive psychotherapy) was defined a priori as 4 or more treatment sessions based on treatment completion thresholds established in other mindfulness clinical trials.^22,23^

The supportive psychotherapy intervention involved discussions about coping with pain, the adverse effects of opioids, and the use of opioids to alleviate negative emotions. To match the homework requirement in the MORE group, participants in the supportive psychotherapy group were asked to write on weekly session topics in a journal for 15 minutes per day and to log minutes spent writing in their journal each day via their smartphones. During supportive psychotherapy, social workers used empathic responding techniques, elicited emotional expression from participants, and promoted a supportive climate but eschewed discussions about mindfulness and did not provide therapeutic skill training. Participants were guided via reflective listening techniques to express emotions and thoughts about group topics and to provide advice and emotional support to their peers. Supportive psychotherapy interventions, which typify a widely available form of conventional process-oriented client-centered therapy,^24^ have been shown to reduce chronic pain.^25^ Our supportive psychotherapy intervention was validated as a control condition in previous mindfulness clinical trials,^16,17,19^ which found no significant difference in treatment credibility ratings between mindfulness interventions and supportive psychotherapy.

Sessions were audio recorded, and treatment fidelity was monitored using validated measures.^26^ Therapist adherence and competence measurements were excellent, indicating that therapists skillfully adhered to each of the standardized protocols with no treatment diffusion (eMethods 1 in Supplement 2).

### Coprimary Outcomes

Outcomes were collected at baseline, after treatment, and at 3, 6, and 9 months of follow-up. Our prespecified coprimary opioid misuse outcome was assessed using the Drug Misuse Index (DMI), a validated composite measure.^12^ Because no single criterion-standard opioid misuse measure exists, the DMI uses 3 levels of data to characterize opioid misuse: (1) self-reports from the COMM (score range, 0-68 points, with higher scores indicating greater likelihood of current opioid misuse); (2) clinical assessments of opioid misuse with the Addiction Behaviors Checklist (score range, 0-20, with higher scores indicating more aberrant drug-related behaviors),^27^ which were rated by clinical staff (ie, psychologists, social workers, and nurses) who were blinded to treatment assignment; and (3) urine toxicologic screening results. Participants were informed that reports of opioid misuse and OUD symptoms would not be disclosed to their physicians and were protected by a National Institutes of Health Certificate of Confidentiality. Scores of 9 or greater on the COMM and 2 or greater on the Addiction Behaviors Checklist were considered positive results. Positive ratings on the urine screening were given when participants received positive results for illicit drugs or nonprescribed opioid medications. Participants with positive COMM scores were given a positive rating on the DMI (ie, a score of 1). If COMM scores were inconsistent with opioid misuse (ie, scores <9 points), then positive results on both the Addiction Behaviors Checklist and urine screening were needed for a positive DMI rating because urine screening can be inaccurate as a result of false-positive results and variable drug metabolites, and clinician ratings may be unreliable. Otherwise, participants were given a negative DMI rating (ie, a score of 0). Multiple studies have used this DMI scoring method.^12,28,29,30,31^

Our coprimary chronic pain outcome was measured using 2 separate subscales (pain severity and pain-related functional interference) of the Brief Pain Inventory (BPI).^32^ Both subscales are scored from 0 to 10 points, with higher scores on the pain severity subscale indicating more severe pain and higher scores on the pain-related functional interference subscale indicating greater impairments in daily functioning.

### Secondary Outcomes

The morphine-equivalent daily dose was assessed by the Timeline Followback assessment method,^9^ in which individuals were prompted with validated interview procedures to recall their opioid use during the period between the previous and current assessment.^33^ Emotional distress was measured using the Depression Anxiety Stress Scale, version 21 (score range, 0-63 points, with higher scores indicating more severe symptoms of depression, anxiety, and stress).^34^ Opioid craving during daily life was assessed on a scale of 0 to 10 (with higher scores indicating more severe craving) using ecological momentary assessments,^35^ which were prompted by a text link sent to the participant’s smartphone at 3 random times per day throughout the intervention period and for 1 month after the intervention period (for a total of 90 days).

### Adverse Events

Adverse events were systematically queried at each study visit. All adverse events were reviewed by an independent medical monitor.

### Statistical Analysis

Based on dichotomous classification of opioid misuse in a pilot study^16^ (63% improvement among individuals receiving MORE vs 32% improvement among individuals receiving supportive psychotherapy), we determined that 200 participants (after 30% attrition) would provide power greater than 0.90 to detect DMI differences of this extent. Assuming moderately correlated repeated measures, this sample size would also provide power greater than 0.90 to estimate small clinical effects on the BPI pain severity and pain-related functional interference measures and other continuous outcomes (Cohen *d* in pilot studies of MORE were 0.50-0.84^16^). We planned to enroll 260 participants to account for unavailability for follow-up; the actual number of participants enrolled was 250.

Following prespecified analysis plans, intention-to-treat analyses were performed using full information maximum likelihood estimation, which yielded unbiased population estimates based on missing at random mechanisms. The primary dichotomous DMI outcome was analyzed using a generalized linear mixed model with a logistic link and random intercept. By design, all participants began the clinical trial with a positive DMI rating, so there was no baseline adjustment for this outcome. Primary BPI outcomes were analyzed (conditioned on prerandomization baseline values) using mixed-effects repeated-measures analyses of covariance. The primary fixed effect of interest was the adjusted (unadjusted for DMI rating) treatment main effect, which estimated the mean overall benefit of MORE vs supportive psychotherapy across all follow-up visits. Results of supplementary treatment-by-time interaction tests are available in eMethods 2 in Supplement 2. To model serial dependence, maximum likelihood models specified random intercepts, which generated compound symmetry covariances with autoregressive error if needed to improve model fit. We examined clinic and group cohort as random effects; because of minimal variance, these random effects left all model estimates essentially unchanged, so we omitted them from the final models for statistical parsimony (eMethods 3 in Supplement 2). Opioid dose (log transformed to reduce skew) and emotional distress outcomes were analyzed similarly. Continuous outcomes were reported as model-based estimates in the original scale measurement. Sample proportions of participants achieving clinically important outcomes for opioid misuse, pain, and opioid dose measures at 9 months were reported. Ecological momentary assessments of opioid craving were analyzed using a similar mixed model, in which the primary effect of interest was the difference in craving trajectory across the 270 possible ecological momentary assessments per participant. To control for false discovery in coprimary outcomes, we compared the unadjusted *P* values with Bonferroni-adjusted (α = .05 divided by 3) *P* = .0167. To control for false discovery in secondary outcomes, we conducted a global hypothesis test (with significance threshold of α = .05) of no treatment difference on any measure using a multivariate mixed-effects linear model. Data were analyzed using SAS software, version 9.4 (SAS Institute Inc), and MPlus software, version 8.5 (Muthen & Muthen).

Participants who missed follow-up visits were permitted to participate at later time points. No missing data were imputed. Likelihood methods provided consistent estimates of quantities of interest when data were missing at random. Sensitivity analyses were performed to assess possible departures from the missing at random assumption (eMethods 4 in Supplement 2). Data cannot be considered not missing at random when the probability of missingness depends on the underlying extent of the outcome (such as study withdrawal due to a disinclination to report opioid misuse or nonresponse to the study interventions). We used a simple selection model for data not missing at random^36,37^ to incorporate dependence into likelihood estimation. Sensitivity to data missing at random was also evaluated using full information maximum likelihood estimation multivariate analyses that introduced a larger set of auxiliary demographic and clinical variables expected to correlate with missingness (designated as the expanded missing at random model), an alternative to multiple imputation that often produces more accurate and precise parameter estimates.^36^ We also conducted pattern mixture models in which participants in the MORE group who withdrew from the study were assumed to respond similarly to participants receiving supportive psychotherapy, providing a conservative lower-bound estimate of the treatment effect based on the assumption that MORE conferred no benefit after discontinuation.

## Results

### Participant Characteristics

**Figure 1.**
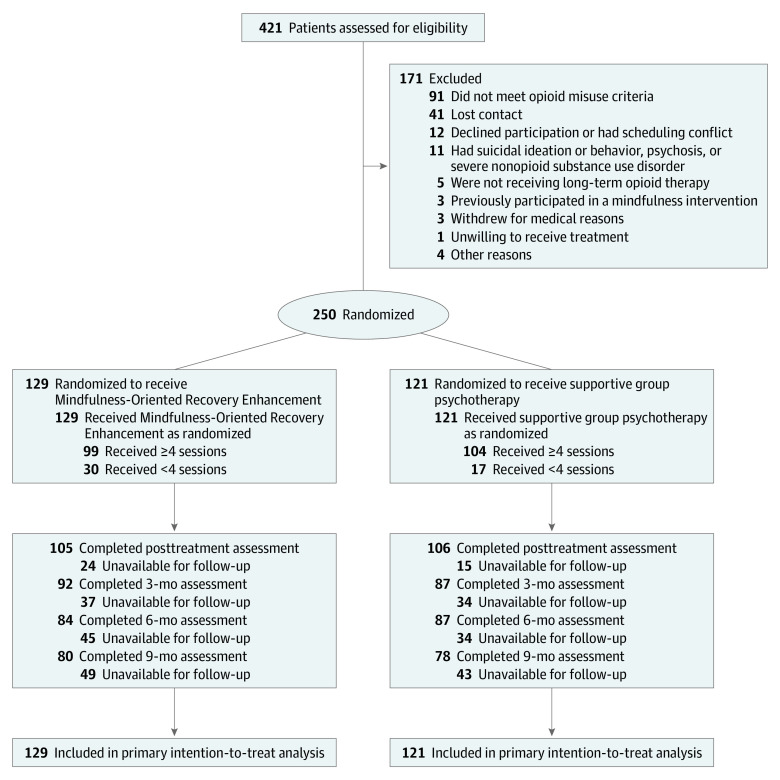
CONSORT Flow Diagram Follow-up was affected by the onset of the COVID-19 pandemic.

We assessed 421 patients for eligibility and enrolled 250 participants (129 randomized to the MORE group and 121 to the supportive psychotherapy group) (Figure 1). Of those, 159 participants (63.6%) were women and 91 (36.4%) were men, with a mean (SD) age of 51.8 (11.9) years; 17 participants (6.8%) were Hispanic or Latino, 218 (87.2%) were White, and 15 (6.0%) were of other races and/or ethnicities (2 American Indian, 3 Asian, 1 Black, 2 Pacific Islander, and 7 did not specify). At baseline, the mean pain duration was 14.7 years (range, 1-60 years), most commonly occurring in the lower back (171 participants [68.4%]); however, 190 participants (76.0%) reported having 2 or more pain conditions. The mean (SD) BPI pain severity score was 5.5 (1.5) points, the mean (SD) COMM score was 17.6 (7.6) points, and the mean (SD) morphine-equivalent daily dose was 101.0 (266.3) mg (IQR, 16.0-90.0 mg). Most participants (173 [69.2%]) were prescribed oxycodone or hydrocodone; a smaller number (27 participants [10.8%]) were prescribed methadone or buprenorphine for pain or OUD. Most participants met the criteria for major depression (171 participants [68.4%]) and OUD (157 participants [62.8%]) based on Mini International Neuropsychiatric Interview scores at baseline.^21^ Participants in the MORE group attended a mean (SD) of 5.5 (2.4) sessions, and participants in the supportive psychotherapy group attended a mean (SD) of 5.8 (2.0) sessions. Demographic and baseline clinical characteristics were similar between the 2 groups (eg, MORE group vs supportive psychotherapy group: mean [SD] age, 50.9 [12.4] vs 52.8 [11.3] years; mean [SD] COMM score, 17.2 [7.0] vs 18.4 [8.1] points; mean [SD] pain duration, 14.6 [11.1] vs 14.7 [10.1] years) (Table 1).

A total of 203 participants (81.2%) completed treatment (mean [SD], 5.7 [2.2] sessions). Participants who discontinued the study did not differ from those who completed the study with regard to psychiatric diagnoses or baseline values of any outcome. Discontinuation rates did not differ significantly by group; at 9 months, 92 of 250 participants (36.8%) discontinued the study: 49 of 129 participants (38.0%) in the MORE group and 43 of 121 participants (35.5%) in the supportive psychotherapy group were unavailable for follow-up. These discontinuation rates were similar to those observed in clinical trials of psychosocial treatments for OUD.^38^

### Outcomes

**Figure 2.**
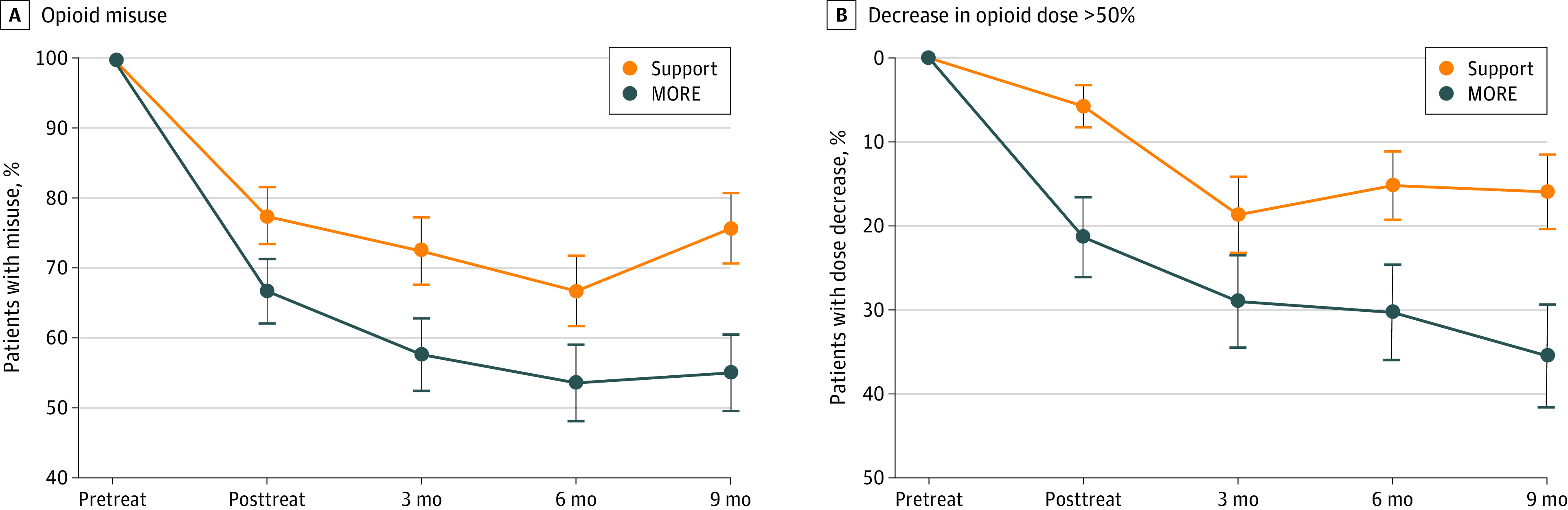
Opioid Outcomes Through 9-Month Follow-up Period for Mindfulness-Oriented Recovery Enhancement and Supportive Psychotherapy A, Measured by the Drug Misuse Index (with 1 indicating a positive rating and 0 indicating a negative rating). B, Measured in morphine-equivalent daily dose. Error bars represent SEs. MORE indicates Mindfulness-Oriented Recovery Enhancement; and Support, supportive psychotherapy.

**Figure 3.**
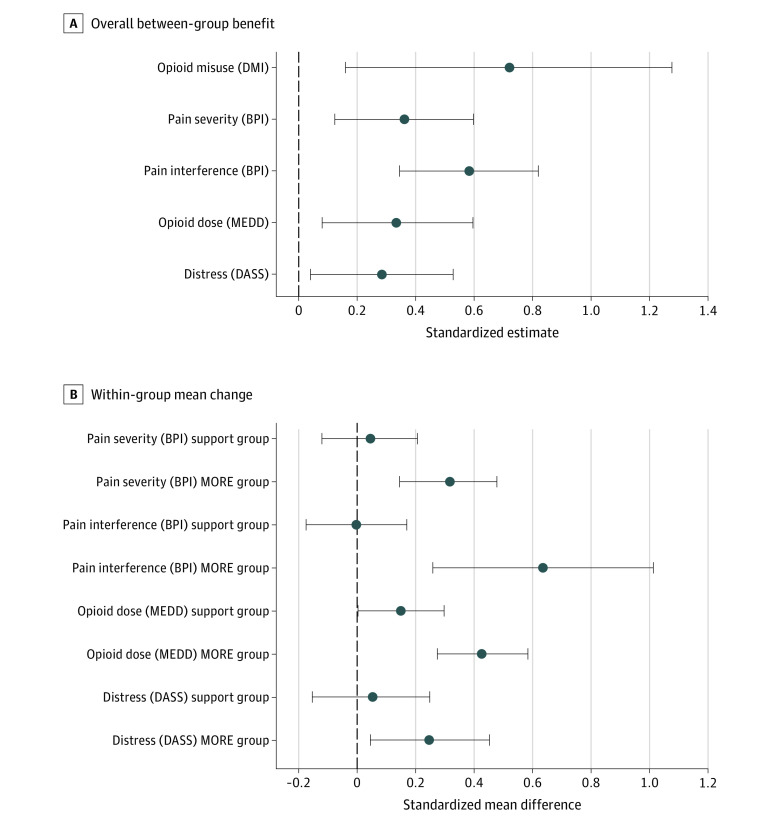
Standardized Estimates of Between-Group and Within-Group Differences A, Standardized point estimate of between-group differences (baseline adjusted and averaged over all follow-up visits) with 95% CIs (represented by horizontal line lengths), with positive directional effect indicating an improvement in the Mindfulness-Oriented Recovery Enhancement (MORE) group relative to the supportive psychotherapy (support) group. B, Standardized point estimate of within-group change from baseline to 9-month follow-up (standardized mean differences) with 95% CIs (represented by horizontal line lengths). Standardized estimates are interpretable in terms of Cohen *d* small (0.2), moderate (0.5), and large (0.8) effect sizes. BPI indicates Brief Pain Inventory; DASS, Depression Anxiety Stress Scale; DMI, Drug Misuse Index; and MEDD, morphine-equivalent daily dose.

The odds ratio for reduction in opioid misuse through 9 months of follow-up in the MORE group compared with the supportive psychotherapy group was 2.06 (95% CI, 1.17-3.61; *P* = .01), corresponding to a risk difference of 0.15 (Figure 2; Table 2). At 9 months, 36 of 80 participants (45.0%) in the MORE group were no longer misusing opioids compared with 19 of 78 participants (24.4%) in the supportive psychotherapy group. Participants in the MORE group also experienced greater reductions in pain severity (between-group effect: 0.49; 95% CI, 0.17-0.81; *P* = .003) and pain-related functional interference (between-group effect: 1.07; 95% CI, 0.64-1.50; *P* < .001) than participants in the supportive psychotherapy group (Figure 3; Table 2). Based on guidelines from the Initiative on Methods, Measurement, and Pain Assessment in Clinical Trials,^39^ a greater number of participants in the MORE group vs the supportive psychotherapy group achieved minimally clinically important reductions in pain severity (35 of 70 participants [50.0%] vs 22 of 75 participants [29.3%]) and pain-related functional interference (41 of 71 participants [58.6%] vs 19 of 75 participants [25.3%]) at the 9-month follow-up (eTable 2 in Supplement 2). The standardized point estimates of MORE vs supportive psychotherapy through the 9-month follow-up for pain severity (0.36) and interference (0.58) exceeded the meta-analytically derived effect size for CBT at follow-up (0.08 and 0.12 for pain severity and disability, respectively) found by de C Williams et al in a meta-analysis.^40^

**Table 2.** Intention-to-Treat Repeated-Measures Analyses for Primary and Secondary Outcomes^a^

Outcome	Pretreatment		Posttreatment		Months of follow-up						Between-group effect^b^					
					3		6		9		MAR model		Expanded MAR model^c^		NMAR model	
	Probability	Participants, No.	Probability	Participants, No.	Probability	Participants, No.	Probability	Participants, No.	Probability	Participants, No.	OR (95% CI)	*P* value	OR (95% CI)	*P* value	OR (95% CI)	*P* value
**Opioid misuse**																
DMI^d^																
MORE	1.00	129	0.71	105	0.59	92	0.54	84	0.54	80	2.06 (1.17-3.61)	.01	2.15 (1.43-3.27)	.008	2.11 (1.17-3.75)	.01
Supportive psychotherapy	1.00	121	0.82	106	0.75	87	0.66	87	0.78	78						
**Chronic pain**																
BPI pain severity, mean (SE)^e^																
MORE	5.65 (0.13)	129	4.93 (0.14)	97	4.91 (0.14)	81	5.13 (0.15)	73	4.86 (0.15)	70	0.49 (0.17-0.81)	.003	0.46 (0.14-0.78)	.005	0.28 (0.06-0.50)	.01
Supportive psychotherapy	5.24 (0.14)	121	5.33 (0.14)	97	5.42 (0.15)	80	5.51 (0.14)	82	5.51 (0.15)	75						
BPI functional interference, mean (SE)^e^																
MORE	6.47 (0.16)	129	5.41 (0.18)	97	5.34 (0.19)	81	5.19 (0.20)	73	4.91 (0.20)	70	1.07 (0.64-1.50)	<.001	1.03 (0.59-1.47)	<.001	0.41 (0.19-0.63)	<.001
Supportive psychotherapy	6.14 (0.19)	121	6.19 (0.18)	97	6.34 (0.20)	80	6.28 (0.20)	82	6.30 (0.20)	75						
DASS emotional distress, mean (SE)^f^																
MORE	21.45 (1.03)	125	16.99 (0.98)	94	18.48 (1.04)	80	18.23 (1.10)	67	18.78 (1.22)	68	2.79 (0.41-5.18)	.02	3.05 (0.63-5.47)	.01	0.28 (0.04-0.52)	.02
Supportive psychotherapy	22.77 (0.98)	119	21.36 (0.99)	91	19.92 (1.05)	71	21.17 (1.07)	77	20.77 (1.16)	72						
**Opioid medication**																
MEDD, median (IQR), mg^g^																
MORE	35.0 (15.0-80.0)	101	24.0 (10.0-62.0)	81	21.0 (8.0-66.5)	77	18.5 (6.0-75.0)	75	23.0 (6.0-75.5)	70	0.15 (0.03-0.27)	.009	0.15 (0.03-0.27)	.01	0.27 (0.07-0.47)	.008
Supportive psychotherapy	38.0 (18.0-90.5)	105	39.0 (18.5-83.5)	89	30.0 (12.5-90.0)	77	35.0 (17.0-86.0)	83	38.0 (14.0-82.5)	73						

Abbreviations: BPI, Brief Pain Inventory; DASS, Depression Anxiety Stress Scale; DMI, Drug Misuse Index; MAR, missing at random; MEDD, morphine-equivalent daily dose; MORE, Mindfulness-Oriented Recovery Enhancement; NMAR, not missing at random; OR, odds ratio.

^a^ Posttreatment and follow-up data are maximum likelihood estimates based on the longitudinal statistical model. Baseline prerandomization descriptive sample means are provided.

^b^ Point estimates indicate the main effect of treatment (ie, the between-group effect adjusted for baseline and averaged over time).

^c^ Included data missing at random plus age, sex, income, educational level, and the full set of repeated dependent outcomes at each time point

^d^ Data are reported as the probability of having a positive DMI rating (ie, a score of 1 vs 0) indicating opioid misuse. The DMI model was not baseline adjusted because all participants had a positive DMI rating at baseline.

^e^ The BPI model was baseline adjusted. The score range of both BPI subscales was 0-10, with higher scores on the pain severity subscale indicating more severe pain and higher scores on the pain-related functional interference subscale indicating greater impairments in daily functioning.

^f^ The DASS model was baseline adjusted. The score range of the DASS was 0-63, with higher scores indicating more severe symptoms of depression, anxiety, and stress.

^g^ The MEDD model was baseline adjusted. Morphine-equivalent daily doses were highly skewed and therefore logged for analysis.

All 3 primary outcomes met the Bonferroni-adjusted standard of *P* < .0167. Moreover, the global multivariate test was highly significant (change in χ^2^ = 27.9; df = 5; *P* < .001). Sensitivity analyses indicated similar results for the expanded missing at random model, the not missing at random model, and the pattern-mixture model (eMethods 4 in Supplement 2). The consistency of these findings suggested that the inferential conclusions were robust over a range of missing data mechanisms and scenarios.

With regard to secondary outcomes, the MORE intervention reduced the morphine-equivalent daily dose to a greater extent than supportive psychotherapy (between-group effect: 0.15 log mg; 95% CI, 0.03-0.27 log mg; *P* = .009) (Table 2). These effects remained significant after excluding participants who were prescribed methadone or buprenorphine. By the 9-month follow-up visit, 22 of 62 participants (35.5%) in the MORE group had decreased their opioid dose by at least 50% compared with 11 of 69 participants (15.9%) in the supportive psychotherapy group (*P* = .009) (Figure 2). Participants in the MORE group also experienced greater decreases in emotional distress than participants in the supportive psychotherapy group (between-group effect: 2.79 points; 95% CI, 0.41-5.18 points; *P* = .02) (Table 2; Figure 3), who showed little improvement. This treatment effect was, in large part, a result of decreases in depression subscale scores (between-group effect: 2.19 points; 95% CI, 0.73-3.66 points; *P* = .004) to levels lower than those below the threshold for major depressive disorder.^34^ Results from the sensitivity analyses were consistent with the missing at random findings. Opioid craving ratings measured by ecological momentary assessments decreased by 0.49 points (95% CI, 0.27-0.70 points; *P* < .001) more in the MORE group vs the supportive psychotherapy group.

### Adverse Events

No adverse events related to the MORE or supportive psychotherapy interventions occurred (eTable 1 in Supplement 2). Seventeen participants (7 in the MORE group and 10 in the supportive psychotherapy group) reported experiencing unrelated adverse events (eg, pneumonia and nephritis).

## Discussion

In this randomized clinical trial, among participants with chronic pain who were misusing opioids at the beginning of the study, the MORE intervention led to large and statistically significant decreases in opioid misuse compared with supportive psychotherapy. In addition, MORE led to greater improvements in pain severity and functional interference than supportive psychotherapy; these therapeutic effects were larger than the effect size of CBT (the current criterion standard for treatment of psychological pain) reported in a meta-analysis.^40^ Participants in the MORE group also reduced their daily opioid dose to a greater extent than those in the supportive psychotherapy group. Finally, compared with supportive psychotherapy, MORE decreased emotional distress, depressive symptoms, and real-time reports of opioid craving in daily life.

Although opioid misuse decreased in the supportive psychotherapy group, possibly reflecting the therapeutic activity of this approach, the effects of the MORE intervention were substantially greater than those of this active control condition. Unlike many interventions with effects that are greatest immediately after treatment but gradually diminish, MORE’s effect was sustained, likely a function of the intervention’s unique mechanisms of action, including enhanced neurophysiologic responsivity to natural, healthy rewards and improved self-regulation of reactivity to opioid-related cues (eg, craving and brain reward responses elicited by the sight of one’s opioid pill bottle).^15,41,42,43,44,45^ By integrating mindfulness with reappraisal and savoring techniques, the MORE intervention aimed to restructure reward processing from valuing drug-related rewards to valuing natural rewards,^15^ a therapeutic focus that is distinct from that of other mindfulness-based interventions, such as mindfulness-based stress reduction.

Before this clinical trial, in addition to pilot studies of MORE,^16,17,18^ only 3 other small studies evaluated behavioral therapies for comorbid chronic pain and opioid misuse.^10,13^ A large clinical trial^46^ recently found that CBT reduced pain but did not reduce opioid use among people receiving LTOT. In contrast, the current full-scale clinical trial demonstrated that MORE could reduce opioid use and misuse while alleviating chronic pain and emotional distress. The MORE intervention’s broad-spectrum effects were noteworthy given that many participants in the sample presented with multiple chronic pain conditions, were taking high opioid doses, and had co-occurring psychiatric disorders. Nonetheless, MORE is an intensive intervention, and brief CBT interventions have been shown to improve chronic pain and emotional distress among individuals who do not misuse opioids.^47^

Furthermore, although not an inclusion criterion, more than 50% of participants met the criteria for OUD on the Mini International Neuropsychiatric Interview. Opioid use disorder is often unrecognized in primary care settings,^48,49^ and most patients with OUD in primary care settings are not receiving medications for OUD.^50^ Although not all patients who misuse opioids have OUD, opioid misuse is a risk factor for OUD.^3,51^ In this clinical trial, the MORE intervention decreased opioid misuse and craving among individuals with comorbid pain and OUD, a population that is especially difficult to treat given that OUD increases pain sensitivity, and pain promotes OUD relapse.^6^ Unlike outpatient addiction specialty care that primarily focuses on treating OUD symptoms, the MORE intervention was delivered in a primary care setting and simultaneously addressed pain and addictive behavior. The MORE approach may be efficacious for the treatment of this challenging comorbidity, as shown in the current clinical trial and reported in previous pilot studies^16,18^; however, additional clinical trials are needed.

### Limitations

This study has several limitations. The clinical trial was limited by its discontinuation rate, which was similar to that of other clinical trials of psychosocial treatment for individuals using opioids (mean discontinuation rate of 42%^38^) and not unexpected given the onset of the COVID-19 pandemic during the final study year (4% of participants completed 9-month follow-up visits in the final study year compared with 74% of participants in the year before the pandemic). Some participants declined to provide follow-up data during the height of the pandemic, and COVID-19–related research restrictions also resulted in missing data. The transient and vulnerable nature of the study sample, who had chronic medical and psychiatric comorbidities, substance use disorders, and poverty status, also contributed to study discontinuation. However, our discontinuation rate was better than that of clinical trials of medications for OUD that had shorter durations of 24 weeks (eg, discontinuation rates of 43% in the study by Haight et al^52^ and 55% in the study by Krupitsky et al^53^). Because study withdrawal rates did not differ between the MORE and supportive psychotherapy groups, missing data were unlikely to bias the outcome analyses toward 1 group vs the other. Sensitivity analyses accounting for data not missing at random continued to show the superiority of the MORE intervention over supportive psychotherapy.

The clinical trial may have been limited by the lack of stratified randomization by LTOT duration or the use of medications for OUD. However, LTOT duration and the proportion of participants receiving medications for OUD did not differ by intervention group, and sensitivity analyses including these covariates did not substantively change the findings. Although we used the DMI, a composite measure that combines data from clinical interviews and urine screening with self-reported opioid misuse scores on the COMM, the COMM is a major component of the DMI, which is a limitation given that it was impossible to conceal treatment assignment from participants. This inability to conceal treatment assignment may have biased participants’ responses on the self-reported COMM measure. Nonetheless, the study was presented to participants as a comparison of 2 behavioral treatments, and no indication was given that participants receiving MORE were in the experimental group.

## Conclusions

In this randomized clinical trial, MORE demonstrated sustained efficacy for improving opioid misuse, opioid dosing, and chronic pain symptoms across 9 months of follow-up. Future comparative-effectiveness clinical trials are needed to determine the impact of MORE relative to other empirically supported interventions (eg, CBT or mindfulness-based stress reduction), and implementation clinical trials are needed to determine the extent to which MORE can be effectively implemented at scale in the context of standard medical care. Integrated health care teams comprising social workers, psychologists, nurses, and/or physicians could potentially deliver MORE in primary care settings via group medical visits.

## Notes

Supplement 1.
Trial Protocol

Supplement 2. **eMethods 1.** Additional Intervention, Training, and Fidelity Monitoring Details **eMethods 2.** Considering Mixed-Effects Models in Which Time Is a Key Component **eMethods 3.** Additional Details on Outcome Analyses **eMethods 4.** Missing Data Strategy and Additional Sensitivity Analyses **eTable 1.** Adverse Events **eTable 2.** Sample Proportions of Patients Achieving a Minimally Clinically Important Reduction in Chronic Pain Symptoms

Supplement 3.
Data Sharing Statement
