# Supplementary material for: Mindfulness-Oriented Recovery Enhancement vs Supportive Group Therapy for Co-occurring Opioid Misuse and Chronic Pain in Primary Care: A Randomized Clinical Trial
Source: JAMA Intern Med. 2022 Feb 28;182(4):407–17. doi: 10.1001/jamainternmed.2022.0033 (PMC8886485; doi:10.1001/jamainternmed.2022.0033)
Supplement: Supplement 1. — Trial Protocol [file jamainternmed-e220033-s001.pdf]

PROTOCOL

Mindfulness-Oriented Recovery Enhancement for Opioid Misuse and Chronic Pain in Primary Care: A Randomized Controlled Trial

This supplement contains the following items:

1. Final protocol with summary of changes, original protocol
2. Final statistical analysis plan with summary of changes, original protocol

TITLE: Mindfulness-Oriented Recovery Enhancement for Opioid Misuse and Chronic Pain in Primary Care: A Randomized Controlled Trial

**PI:**	**ERIC GARLAND, PHD**
**AGENCIES:**	**NATIONAL INSTITUTE ON DRUG ABUSE**
**NIDA GRANT NUMBER:**	**R01DA042033**
**APPLICANT ORGANIZATION:**	**UNIVERSITY OF UTAH**
**DATE OF ORIGINAL IRB APPROVAL:**	**03/25/2015**
**VERSION:**	**2.0**

Title	**Mindfulness-Oriented Recovery Enhancement for Opioid Misuse and Chronic Pain in Primary Care: A Randomized Controlled Trial**
Study Design	The design is a parallel group superiority trial. The unit of randomization is the individual patient.
Study Duration	5 years.
Trial Sites	University of Utah Health – Community Physicians Group.
Objective	Conduct a randomized controlled trial to determine the efficacy of a Mindfulness-Oriented Recovery Enhancement intervention for opioid misuse and chronic pain in primary care.
Number of Subjects	The target sample size is 260 participants enrolled (200 + oversampling by 30% for attrition) to provide 80% probability that a two-sided 95% confidence interval will have a margin (half-width) less than +/- .18 standard deviation for the between-groups difference in the study outcomes.
Main Inclusion Criteria	Age ≥18 with a chronic pain-related diagnosis, reporting current pain ≥3 on a 0-10 scale, currently taking prescription opioids for ≥3 months, and surpassed a validated cutpoint for opioid misuse on the Current Opioid Misuse Measure (COMM).
Intervention	Mindfulness-Oriented Recovery Enhancement (MORE) is a group behavioral intervention that unites mindfulness training, cognitive reappraisal, and positive psychological principles into an integrative intervention strategy targeting mechanisms of pain and opioid misuse.
Duration of Intervention	8 weeks.
Primary Outcome	The primary outcome is opioid misuse measured by the Drug Misuse Index, operationalized as scores on the COMM and triangulated by blinded clinical interview and urine drug screen. The co-primary outcome is chronic pain symptomology, operationalized as pain severity and functional interference scores on the Brief Pain Inventory (BPI).

Primary Analysis	Effects of treatment on the DMI will be analyzed with generalized mixed effects models, and effects on the BPI will be analyzed with mixed effect ANCOVA models, adjusting for baseline levels.
Secondary Outcomes	Opioid dose, psychological distress, and opioid craving.
Interim Analysis	Interim monitoring will focus on patient accrual, baseline comparability of treatment groups, protocol adherence, data completeness and quality, and safety. No interim outcome analysis is planned.

Summary of Changes from Protocol Version 1.0 to Version 2.0

In addition to corrections to typographical errors and spelling mistakes, the following changes were made to convert version 1.0 to version 2.0:

1. Addition of new supplementary recruitment methods including radio advertisements.
2. Increasing participant compensation from $300 to $320 for completing all study assessments and visits.
3. Addition of one tertiary outcome measure.
4. Addition of updated information to the description of the statistical analysis plan in the protocol document.

CONTENTS

1. INTRODUCTION
2. STUDY OBJECTIVES
3. BACKGROUND
4. STUDY DESIGN
4.1 NUMBER OF SUBJECTS
4.2 SUBJECT SELECTION AND WITHDRAWAL
4.2.1 Inclusion and Exclusion Criteria
4.3 SCREENING, RECRUITMENT, AND ENROLLMENT
4.3.1 Screening
4.3.2 Recruitment and Enrollment
4.3.3 Rescreening
4.3.4 Early Withdrawal of Participants
5. STUDY INTERVENTION
6. CONTROL INTERVENTION
7. OUTCOME MEASURES
7.1 DEFINITION AND ASCERTAINMENT OF OUTCOMES
7.1.1 Primary Outcomes
7.1.2 Secondary Outcomes
7.1.3 Tertiary (Mechanistic) Outcomes
7.1.4 Ecological Momentary Assessment (EMA) and Process Measures
8. STUDY PROCEDURES AND VISITS
8.1 RANDOMIZATION
8.2 SCHEDULE OF EVENTS
9. STATISTICAL ANALYSIS, SAMPLE SIZE AND POWER CALCULATIONS
9.1 DESIGN OVERVIEW
9.2 SUBJECT ALLOCATION TO TREATMENT ARMS
9.3 SAMPLE SIZE
9.4 SAMPLE SIZE AND POWER FOR SECONDARY OUTCOMES
9.5 INTERIM MONITORING
9.6 STATISTICAL ANALYSIS PLAN
10. DATA MANAGEMENT
10.1 RESEARCH MATERIALS HANDLING
10.2 HANDLING OF STUDY TREATMENT AUDIO RECORDINGS
10.3 RESEARCH MATERIALS HANDLING
10.4 DATA MONITORING PROCEDURES
10.5 DATA RISK REPORTING
10.6 DATA INTEGRITY

11. SAFETY AND ADVERSE EVENTS
11.1 BACKGROUND
11.2 DEFINITIONS
11.3 RESPONSIBILITIES
11.4 ASCERTAINMENT OF AE, UNANTICIPATED PROBLEMS (UP), AND SAE
11.5 SAFETY PERSONNEL
11.6 REPORTING OF UNANTICIPATED PROBLEMS AND SERIOUS ADVERSE EVENTS
12. ETHICAL AND REGULATORY CONSIDERATIONS
12.1 GOOD CLINICAL PRACTICE STATEMENT
12.2 INFORMED CONSENT
12.3 INSTITUTIONAL REVIEW BOARD
12.4 RISKS TO HUMAN SUBJECTS
12.4.1 Protection Against Risks
12.4.2 Potential Benefits of the Proposed Research to Human Participants and Others
12.4.3 Importance of the Knowledge to be Gained
12.5 INCLUSION OF WOMEN AND MINORITIES
13. STUDY DOCUMENTS
13.1 RETENTION OF RECORDS
14. PUBLICATION AND DISSEMINATION POLICY
14.1 DISSEMINATION

1. INTRODUCTION

This document is a protocol for a human research study to be conducted according to US and international standards of Good Clinical Practice (FDA Title 21 part 312 and International Conference on Harmonization guidelines), applicable government regulations, and institutional research policies and procedures.

2. STUDY OBJECTIVES

The proposed parallel-group randomized clinical trial will determine the efficacy of Mindfulness-Oriented Recovery Enhancement (MORE) as a treatment for opioid misuse and chronic pain in a primary care setting. Design features of this study draw upon strengths of Stage 2 and Stage 3 research per the NIH Stage Model,[1] maximizing internal validity, intervention potency, and fidelity assurance with research therapists while maximizing external validity by delivering the intervention in community clinics – the setting where most chronic pain patients seek care.[2]

MORE integrates training in mindfulness, reappraisal, and savoring of natural rewards to enhance hedonic regulation and target the risk chain at the point where maladaptive emotion-cognition interactions link chronic pain to opioid misuse. MORE is innovative in that it aims to modify associative learning mechanisms dysregulated by the allostatic effects of opioid misuse on brain reward systems via strengthening top-down cognitive control to restructure bottom-up reward learning from valuation of drug reward to natural reward – something that no other behavioral intervention for opioid misuse has been designed to do.

MORE was originally tested in a university setting as an intervention for opioid misuse among patients prescribed opioids for chronic pain in an R03-funded, Stage I pilot randomized controlled trial (RCT). Results demonstrated that MORE significantly decreased opioid misuse and opioid craving, as well as reduced chronic pain severity and functional impairment, relative to a support group (SG) control.[3] Notably, MORE significantly reduced opioid cue-reactivity and enhanced neurophysiological responsiveness to natural reward stimuli relative to the SG control, providing compelling preliminary evidence that MORE may restructure reward processing.[4,5]

The Specific Aims of this study are as follows:

AIM 1. To conduct a RCT of the Mindfulness-Oriented Recovery Enhancement (MORE) intervention for co-occurring aberrant drug-related behaviors and chronic pain in primary care.
We will compare the therapeutic impact of MORE and a conventional supportive group (SG) psychotherapy active control condition on clinical outcomes germane to opioid misuse and chronic pain. **Hypotheses**: Opioid misusing patients assigned to MORE, as compared to SG participants, will evidence decreased opioid misuse and pain (*PRIMARY OUTCOMES*), as well as reduced opioid craving, opioid dosing, and psychological distress (*SECONDARY OUTCOMES*) from pre- to post-treatment through 9-month follow-up.

AIM 2. To test and quantify the degree to which MORE's impact on aberrant drug-related behaviors and pain is mediated by proactive control over emotion-cognition interactions (top-down mechanism).
Hypotheses: The impact of MORE on opioid misuse and pain will be mediated by improvements in: **a)** attentional disengagement from opioid-related cues (reduced attentional bias); **b)** emotion regulation**;** and **c)** cognitive coping (e.g., reinterpretation of pain as innocuous sensory information).

AIM 3. To test and quantify the degree to which MORE's impact on aberrant drug-related behaviors is mediated by restructuring of reward processing (bottom-up mechanism).
Hypotheses: The impact of MORE on opioid misuse will be mediated by restructured reward processing, as indicated by a shift in the relative responsiveness to opioid and natural reward cues.

3. BACKGROUND

Prescription opioid misuse among chronic pain patients is an emerging public health threat that is being addressed with heightened urgency at both clinical and policy levels. Though opioid analgesic therapy can manage chronic pain, it may confer with significant health risks, including dependence, overdose, and misuse. Opioid misuse is evidenced by aberrant drug-related behaviors such as unauthorized dose escalation or use of prescribed opioids to self-medicate negative emotions that exacerbate craving.[6] Research on treatments for opioid misuse among chronic pain patients is scant; according to a 2015 NIH-AHRQ systematic review of long-term opioid therapy for chronic pain, "no study evaluated the effectiveness of risk mitigation strategies for improving outcomes related to overdose, addiction, abuse, or misuse."[7] Extant therapies may have limited efficacy because they fail to directly target cognitive-affective neural circuits that govern hedonic regulatory responses elicited by pain-, opioid-, and reward-related stimuli.[8] To address this need, the PI translated mechanistic findings from behavioral neuroscience into an innovative treatment for prescription opioid misuse, called **Mindfulness-Oriented Recovery Enhancement** (MORE). MORE integrates training in mindfulness, reappraisal, and savoring of natural rewards to enhance hedonic regulation and target the risk chain at the point where maladaptive emotion-cognition interactions link chronic pain to opioid misuse. MORE is innovative in that it aims to modify associative learning mechanisms dysregulated by the allostatic effects of opioid misuse on brain reward systems via strengthening **top-down cognitive control to restructure bottom-up reward learning from valuation of drug reward to natural reward** – something that no other behavioral intervention for opioid misuse has been designed to do. Accordingly, this project funded by the National Institute on Drug Abuse (NIDA) is a randomized clinical trial (RCT) of MORE to reduce opioid misuse and chronic pain symptoms among patients receiving long-term opioid therapy in primary care.

4. STUDY DESIGN

A two-arm parallel RCT design with an active control group will minimize internal threats to validity. Mindfulness-Oriented Recovery Enhancement (MORE; n=130) and the Supportive Group Psychotherapy control (SG; n=130) will be equated for time spent in treatment. Assessors will be blind to treatment condition. Target sample sizes were determined via power and precision analyses. The unit of randomization is the individual patient. The co-primary outcomes are opioid misuse and chronic pain symptoms. Participants will complete a clinical assessment battery at pre- and post-treatment, and at 3-, 6-, and 9-month follow-ups. Participants will complete a psychophysiological assessment protocol at pre- and post-treatment. Participants will complete ecological momentary assessments (EMAs) of symptoms, skill practice, and opioid use during treatment and 1 month afterward up to 3 times/day (morning, noon, and night).

4.1 Number of Subjects

Our estimates indicate that a target sample size of 260 participants will have 80% probability that a two-sided 95% confidence interval will have a margin (half-width) less than +/- .18 standard deviation for the between-groups difference in the study outcomes.

4.2 Subject Selection and Withdrawal

4.2.1 Inclusion and Exclusion Criteria

Table 1 lists the inclusion and exclusion criteria for screening patients for participation in the trial.

Table 1: Inclusion and Exclusion Criteria
**Inclusion Criteria**<br>• Age 18-60+<br>• Current back pain diagnosis as determined from ICD-9 codes in claims data (including but not limited to ICD-9 diagnoses 724.x, or 847.x) or current chronic pain diagnosis determined by physician assessment (including but not limited to ICD-9 diagnoses 338.0, 338.21, 338.22, 338.28, 338.29, 338.4)<br>• Current use of prescription opioid agonist or mixed agonist-antagonist analgesics for >90 days, and evidence of opioid misuse as indicated by the Current Opioid Misuse Measure<br>• Willingness to participate in study interventions and assessments
**Exclusion Criteria**<br>• Prior experience with Mindfulness-Based Stress Reduction, Mindfulness-Based Cognitive Therapy, or Mindfulness-Based Relapse Prevention<br>• Active suicidality, schizophrenia, psychotic disorder, and/or severe non-opioid substance use disorder<br>• Presence of clinically unstable systemic illness judged by physician to interfere with treatment

Because participants must be able to receive the MORE or SG treatments in English, it is not feasible to enroll participants who do not speak English.

4.3 Screening, Recruitment, and Enrollment

4.3.1 Screening

Patients aged 18 or older will be identified via the University of Utah Health system electronic health record (EHR) and subsequently screened. Patients will also be referred by their health care practitioner into the study, or notified about the study via print, radio, and online advertisements. Screening will occur in clinic, over the phone, or in person at the Center on Mindfulness and Integrative Health Intervention Development (C-MIIND).

Screening questions assess the duration of prescribed opioid use, the type of opioid prescribed, the patient's pain condition, the patient's pain level while taking opioids, and whether or not the patient meets criteria for opioid misuse, according to the validated opioid misuse cutpoint on the COMM.[6]

4.3.2 Recruitment and Enrollment

Patients who screen positive will be informed about the study during an interview, during which the recruiter will confirm the absence of any exclusion criteria, review the purpose of the study, answer any questions and, after obtaining written informed consent, collect baseline data on: key demographic characteristics, clinical characteristics, and primary, secondary, and mechanistic outcomes. Recruitment staff will be kept blinded to randomization status of the patients and will be rigorously trained to reduce potential bias. Patients will remain blind to the nature of the experimental versus the control interventions by being told that the intention of the study is to compare two forms of group behavioral treatment for chronic pain, one based on mindfulness and the other based on social support. The informed consent process will not identify which condition (MORE or SG) is the experimental intervention and which is the control intervention.

4.3.3 Rescreening

Patients who screened out initially as not meeting study criteria for long-term opioid therapy (<90 days of

opioid use) will be rescreened during subsequent visits. However, screen positive patients who had previously opted out or declined to participate will not be re-contacted.

4.3.4 Early Withdrawal of Participants

Because this trial is conducted via the "intent-to-treat" (ITT) framework, all participants will be analyzed in the group to which they were randomly assigned, regardless of whether they complete the intervention or are noncompliant. This preserves the effect of randomization. We plan to follow participants for the study outcomes even if they elect to stop the study intervention. If the participant elects full withdrawal from the study, this will be recorded and all contact with the participant will cease. Participants who are disruptive, or who develop active suicidality or psychosis, or medical conditions that would preclude completion of study procedures, may be withdrawn at any time by the discretion of the PI.

5. STUDY INTERVENTION

The **MORE arm** will participate in 8 weekly, 2-hour group sessions led by a Master's level clinical social worker. MORE sessions involve mindfulness training to disrupt aberrant drug-related behavior and reduce pain, cognitive reappraisal to decrease negative affect and regulate opioid craving, and savoring to augment natural reward processing and evoke positive emotion. MORE participants will be asked to engage in 15 minutes/day of skill practice at home, and complete daily EMA.

6. CONTROL INTERVENTION

The SG control arm will participate in 8 weekly, 2 hour SG sessions led by a Master's level clinical social worker, discussing topics and expressing emotions related to opioid misuse and chronic pain, in addition to completing daily 15 minute journaling sessions on pain and opioid-related themes and daily EMA. The SG format, which was adapted from the supportive group psychotherapy condition piloted in the PI's R03 and several of our prior RCTs. SG therapists will use a Rogerian client-centered therapy approach. We selected the SG as the control for the proposed R01 based on its success as a credible and active control condition in our preliminary studies of MORE,[3,9] which found no significant difference in ratings of intervention credibility between MORE and SG participants. Self-help and therapist-led SGs are standard care and among the most commonly available treatments for substance use disorders and chronic pain. The SG and MORE groups will be fixed and comprised of 8-10 individuals each.

7. OUTCOME MEASURES

7.1 Definition and Ascertainment of Outcomes

Outcome measures were informed by the Initiative on Methods, Measurement, and Pain Assessment in Clinical Trials (IMMPACT).[10] Outcome measures summarized below.

7.1.1. *Primary outcomes (PRE/POST/FOLLOW-UP).* Our pre-specified primary opioid misuse outcome in clinicaltrials.gov is a validated composite measure - the Drug Misuse Index (DMI).[11] Because there is no single way to identify patients who misuse opioids,[12] the DMI uses 3 levels of data to triangulate opioid misuse: self-reports on a structured questionnaire (i.e., the COMM); clinical assessment of opioid misuse—the Addiction Behaviors Checklist (ABC)[13]—rated by clinical staff (i.e., psychologists, social workers, and nurses) blinded to treatment assignment; and urine toxicology screens. The COMM consists of 17 items rated on a Likert scale (0=never, 4=very often) regarding how often in the past 30 days patients engaged in opioid misuse-related behaviors (e.g., used opioids in ways other than prescribed). COMM scores ≥9 and ABC scores ≥2 are considered positive. A positive rating from the urine screen is given when subjects test positive for illicit drugs or a non-prescribed opioid. Subjects with positive COMM scores will be given a positive DMI and classified as a misuser. If patients deny engaging in opioid misuse-related behavior (COMM scores <9), then positive ratings on both the urine screen and the ABC are needed for a positive DMI, because urine screens can be inaccurate due to variable drug metabolites, and clinician ratings may unreliable.[14,15] Otherwise, subjects will be given a negative DMI. Multiple studies support the validity of this DMI scoring method.[11,16–18] **Chronic pain symptoms** (pain severity and functional interference) will be assessed with the Brief Pain Inventory (BPI).[19]

7.1.2. <u>*Secondary outcomes (PRE/POST/FOLLOW-UP).*</u> **Opioid craving** will be measured with 2 items "How much do you want to take your opioids right now?" and "How strong of an urge do have to take opioids right now?" using 0-10 Numeric Rating Scales (NRS) delivered via EMA. Single item measures of craving have been shown to validly predict opioid misuse[16] and be sensitive to MORE treatment effects.[3] **Reduction in opioid dose** will be assessed by the Timeline Followback Procedure (TLFB) and triangulated by chart review of prescription history by the study coordinator.[20] Opioid dose will be converted to oral morphine-equivalent using standardized equianalgesic conversions. Subjects will also document daily opioid dose via EMA. **Psychological distress** will be measured with the Depression Anxiety Stress Scale-21 (DASS-21), a validated, reliable scale (α=.87-.94).[21]

<u>7.1.3. Tertiary (mechanistic) outcomes *(PRE/POST)*.</u>
<u>*Self-report mediators (PRE/POST).*</u> **Reinterpretation of pain sensations** as innocuous sensory information will be assessed with that validated subscale on the Coping Strategies Questionnaire (CSQ).[22] In the PI's R03, we found that effects of MORE on pain were mediated by increases in the Reinterpreting Pain Sensations of the CSQ.[3] **Mindfulness** will be measured with the Five Facet Mindfulness Questionnaire (FFMQ),[23] which the PI found in his R03 to mediate effects of MORE on pain.[3] **Reappraisal** will be measured with the positive reappraisal subscale of the Cognitive Emotion Regulation Questionnaire (CERQ)[149] which mediated the stress-reductive effects of mindfulness in an earlier study.[107] **Savoring** will be measured with the momentary savoring subscale from the Ways of Savoring Checklist (WOS), which mediated impact of daily positive events on positive mood in a prior study.[24]

<u>*Cognitive-affective tasks and psychophysiology (PRE/POST).*</u> See ancillary mechanistic study protocol.
<u>*7.1.4. Other tertiary (mechanistic) measures*</u>. **Trait positive and negative affect** will be measured with the PANAS. **Pain catastrophizing** will be measured with the CSQ. **Meaning in life** will be measured with the Meaning in Life Questionnaire. **Self-transcendence** will be measured with our own in-house instrument (in development). **Post-traumatic stress symptoms** will be measured with the Posttraumatic Symptom Checklist-Civilian Version (PCL-C). **Anhedonia** will be measured with the Snaith Hamilton Anhedonia and Pleasure Scale (SHAPS).

<u>7.1.5. *EMA and process measures*</u>. In symptom diaries 3 times/day, **opioid craving, pain intensity** and **unpleasantness,** and **positive and negative affect** will be assessed with 0-10 Numeric Rating Scales (NRS). Opioid dosing and amount of **mindfulness, reappraisal, and savoring skill practice** will be assessed at the end of the day. **State mindfulness** will be measured after each treatment session with the validated Toronto Mindfulness Questionnaire (TMS).[25]

8. STUDY PROCEDURES AND VISITS

8.1 Randomization

The trial has a two-group parallel randomized design. The unit of randomization is the individual patient. Following pre-intervention assessment, patients will be assigned in a 1:1 ratio to undergo MORE or a supportive group (SG) psychotherapy control condition.

8.2 Schedule of Events

The trial Schedule of Events is shown in **Table 3**.

Event	Screen	Pre -Tx	In Tx	Post-Tx	Follow-Up			
					1 Mo	3 Mo	6 Mo	9 Mo
Screening and Stratification Measures								
Informed Consent	X							
Demographics & Medical History	X							
MINI	X							
Primary and Secondary Outcome Measures								
BPI Pain severity and interference		X		X		X	X	X

DMI: ABC, COMM & Urine screen		X		X		X	X	X
Opioid dosing – TLFB and chartreview		X		X		X	X	X
DASS Psychological distress		X		X		X	X	X
Opioid Craving NRS – EMA (see below)			X			X		
Mediators								
**Self-report mediators:**<br>CSQ Reinterpretation<br>FFMQ Mindfulness<br>CERQ Reappraisal<br>WOS Savoring		X		X		X	X	X
**Other tertiary measures:**<br>PANAS Affect<br>CSQ Catastrophizing<br>MLQ Meaning in Life<br>SHAPS Anhedonia<br>PCL PTSD<br>Self-Transcendence		X		X		X	X	X
**Psychophysiological protocol**		X		X				
Intervention Process Measures and Ecological Momentary Assessments								
TMS State mindfulness (after each session)			X					
Opioid craving NRS - EMA			X			X		
Opioid dosing – EMA			X			X		
Affect NRS - EMA			X			X		
Pain Intensity & Unpleasantness NRS - EMA			X			X		
Therapeutic skill practice			X			X		

9. STATISTICAL ANALYSIS, SAMPLE SIZE AND POWER CALCULATIONS

9.1 Design Overview

This is a two-group, parallel randomized controlled superiority trial. The co-primary outcomes are opioid misuse (measured by the Drug Misuse Index [DMI]) and chronic pain symptoms (measured by the Brief Pain Inventory [BPI]). The target sample size is 260 participants before attrition to detect a small-moderate effect size reduction (Cohen's f = .20) in opioid misuse and pain with 80% power.

9.2 Subject Allocation to Treatment Arms

An electronic random number generator will randomize participants with simple random assignment in blocks of varying sizes (2 - 4) to preserve unpredictability of allocation, which will be concealed by opaque envelopes. To prevent bias and maintain allocation concealment, participants will not be allocated until the first treatment session. Assessments will be conducted by project staff blinded to group assignment (which remained concealed throughout the study). To maintain blinding, the allocation list was inaccessible to project staff involved in assessment or treatment, and before each assessment, participants were reminded to not reveal anything that would disclose their treatment assignment to study staff.

9.3 Sample Size

Minimally detectable effect sizes (Cohen's *f*) for treatment X time interaction (pre-tx, post-tx, follow-up)

Correlation	N = 100	N = 110	N = 120	N = 130
0.25	0.16	0.15	0.14	0.14
0.50	0.13	0.12	0.12	0.11
0.75	0.09	0.09	0.08	0.08

Power calculations are based on three complementary approaches. (a) *Univariate effect sizes*. The PI's R03 identified medium to large effect sizes on pain and opioid-related variables (Cohen's *d*=.50 - .84).[3] We used GPower 3.1 software to determine the smallest detectable effect for power of .80 against a range of sample sizes. The table indicates the smallest detectable effects for a treatment X time interaction term given different sample sizes, assuming a Type I error rate of .05 and varying correlations between repeated measures, which ranged from .34 -.56 per outcome in the R03. Cohen[26] defined *f*=0.10 and *f*=0.25 as small and medium effects, respectively. Thus our target N of 260 (N=200 + oversampling by 30% for attrition) will allow us to detect small-to-medium effects on continuous outcomes. Based on dichotomous clinical classification of opioid misuse data from the R03 (68% improvement for MORE versus 32% for SG), the proposed sample size will offer outstanding power (>.99) to detect differences of this magnitude on this outcome. (b) *Precision of parameter estimation*. Our target N will allow model parameters to be estimated with a high degree of precision. A per group sample size of N=130 provides 80% probability that a two-sided 95% confidence interval will have a margin (half-width) less than +/- .18 standard deviation. Thus the proposed sample will provide precision more than adequate for estimating small clinical effects with high confidence. (c) *Multivariate models*. Generally, structural equation power calculations can be based on Root Mean Square Error of Approximation (RMSEA) comparisons of nested models. The proposed sample provides, in a realistic testing scenario, greater than 80% power in a 1 *df* test (26 versus 25) of close fit comparing two models with RMSEA of .06 and .05 at α=.05. These calculations are conservative. Maximum likelihood techniques retain all observations at all times, and efficiently incorporate correlated observations to yield an Effective Sample Size that is quantifiably larger than the number of subjects. With fully specified repeated dimensions, the anticipated correlations will yield an effective sample size that is 40% larger, or roughly N=360.

9.4 Interim Monitoring

Interim monitoring will focus on patient accrual, baseline comparability of treatment groups, protocol adherence, data completeness and quality, and safety. No interim outcome analysis is planned.

Hypothetical treatment-response distributions

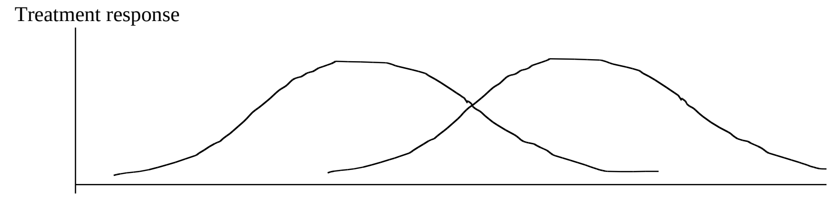

One of the purposes of the intermin monitoring is to provide the data-dependent guidance for the ongoing trial. Series of interim analyses can be conducted to determine whether the data-dependent stopping is called for, due to 1) the presence of overwhelming efficacy of the therapy(ies) in question or 2) "negative stopping" (terminate the project early because there appears to be no chance of a significant positive outcome). The basic premise underlying the former is to make the optimally effective treatment available to all patients as soon as possible. Generally, a sequence of interim analyses are conducted, using adjusted alpha levels (e.g., the Lan-DeMets approach with the O'Brien-Fleming spending function in order not to exceed an overall alpha of 5%). However, we propose that this assumption is problematic with our proposed study given our intent to investigate individual differences in treatment responses (i.e., moderators). Due to the large within-group variability, the presence of between-group differences does not provide specific information about who benefits from what treatment. For example, suppose that we obtain the data of treatment response in each of the 2 treatment groups as depicted in the Figure above. The distance between the two means may be sufficiently distant to show the significant group effect. However, when the within group variability is large, as in the case in many clinical trials in the area of chronic pain and addictions treatment, the presence of a significant between-groups difference does not tell us much about what treatment a clinician should provide given a patient. Thus, it would be premature to set up specific positive stopping roles based upon the interim group comparisons. Similarly, we will not consider the negative stopping. The rationale for this is that

given the pre-specified power calculations based on structural equation models of moderated mediation analyses, we will need to continue the trials until the end to assure the adequate level of statistical power to detect true difference. Thus, we propose that early termination of the project will occur only when the safety analysis of the project indicates as such (see below).

This study will be stopped prior to its completion if: (1) the intervention is associated with adverse effects that call into question the safety of the intervention; (2) any new information becomes available during the trial that necessitates stopping the trial; or (3) other situations occur that might warrant stopping the trial.

9.5 Statistical Analysis Plan

9.5.1. We will inspect scatter plots and descriptive statistics for study variables at pre-treatment, post-treatment, and the four follow-ups to determine the pattern of performance across time. Pearson correlation coefficients will be computed for associations among outcome measures and potential mediators. After data cleaning, we will examine the empirical distribution function of all variables, using the Kolmogorov-Smirnov GOF test and the Shapiro-Wilk normality test. If it is or can be transformed to a Gaussian distribution, parametric tests will be used for analysis. If not, non-parametric methods will be used.

9.5.2. In line with **Aim 1**, effects of treatment on opioid misuse as measured by DMI by 9-month follow-up will be analyzed with generalized linear mixed models with a binary outcome, with fixed effects consisting of a time factor and between-subjects treatment factor (MORE vs. SG). Because all patients will have a positive DMI at baseline (given the inclusion criterion of opioid misuse), models will include data from all post-treatment time points. Effects of treatment on continuous outcomes (will be evaluated using linear mixed models with fixed effects consisting of a time factor and between-subjects treatment factor (MORE vs. SG), and adjust for randomization imbalance by covarying baseline levels of the outcome. Our primary interest lies in the fixed effect of treatment, which with this model specification represents the average effect of treatment across all post-treatment measurement points. We will compare the change in log-likelihood (deviance) of the unrestricted maximum likelihood for the full model including interaction versus a constrained additive model. Random effects will incorporate correlated intercept and trend components, as in standard growth models, with additional dependence modeled, if needed and supported by the Bayesian Information Criterion, as autoregression in the repeated dimension. In addition, treatment group cohort and study therapist will also be modeled as random effects if indicated by the BIC and warranted by the ICC. If we identify a significant treatment X time interaction, we will investigate effects between time points through custom contrasts. Similar mixed models will be estimated for each of the proposed mediators, and mediational effects will be explored more fully in mediation models described below. Little's MCAR test will be used to determine if data are Missing Completely at Random (an ideal that is rarely met with longitudinal data). There is no definitive test for Missing at Random, which describes data that are random conditional on observed data. We will use maximum likelihood estimation (MLE) procedures to deal with missing data according to an intent-to-treat philosophy that is robust against common patterns of missing data (see also our didactic article[27]). MLE is based on all data observations; no values are deleted or imputed. We will conduct follow-ups on all clients regardless of whether or not they complete treatment. To control for false discovery in the three primary outcomes, we will compare the unadjusted p-values against Bonferroni-adjusted alpha =.05/3=.0167. For secondary outcomes, we conducted a global hypothesis test, at alpha=.05, of no treatment difference on any measure using a multivariate mixed effects linear model. Exact p-values will be reported for all comparisons.

9.5.3. To explore whether the cognitive, affective, and physiological mediators specified in **Aims 2 and 3** statistically mediate the effect of treatment on outcome variables, we will conduct structural equation path analyses according to established guidelines[28] by evaluating three regression paths: A) *'a'* path between treatment indicator and change in outcome (e.g., opioid misuse) from pre-treatment to follow-up; B) the *'b'* path between pre-post treatment change in the mediator (e.g., attentional bias) and pre-treatment to follow-up change in the outcome (e.g., opioid misuse); and C) the *'c'* path between treatment and pre-post treatment change in the mediator (e.g., attentional bias). We will test mediation by evaluating the significance of the joint product of the *a* and *b* paths, with bootstrapping[29] used to test the significance of the indirect effect. Our team developed a comprehensive analysis of mediation under possible moderation for clinical trials,[30] allowing for

the possibility that treatment has changed relationships, not just levels, among variables - this will further guide analyses for Aims 2 and 3. Path models will be corrected for multiple comparisons. Mediational analyses will be published in a subsequent report following publication of the primary trial outcomes.

9.5.4. For EMA, we will use growth curve analysis[31] to examine treatment and homework practice effects on: A) trajectories of momentary craving, affect, and pain intensity/unpleasantness; B) relationships among these variables over time; and C) how trajectories in craving, pain, and affect predict variations in opioid dose in separate autoregressive latent trajectory (ALT)[32] models. Growth curve models will be specified with fixed effects consisting of a time factor and between-subjects treatment factor (MORE vs. SG), whereby the treatment X time interaction is the fixed effect of primary interest. As supported by the Bayesian Information Criterion, random effects will incorporate correlated intercept and trend components, as in standard growth models, with additional dependence modeled as autoregression in the repeated dimension. ALT models will estimate cross-lagged and auto-regressive effects, as well as latent intercept and growth factors, among opioid dose and other EMA variables, conditioned on a treatment group variable and process measures of homework practice (e.g., mindfulness, reappraisal, and savoring). EMA models will be corrected for multiple comparisons.

10. DATA MANAGEMENT

Data will be collected from the participants for research purposes only. Questionnaire data, clinical interview data, urine screen data, computerized cognitive task and psychophysiological data, and audio recorded materials will be the principal materials. They will be collected from the subjects at pre and post-intervention, as well as at each of the tri-monthly follow-ups, and weekly after the intervention sessions. These materials will be stored in the locked office of the PI prior to and during processing. Data management will include entry of pre-, post- and follow-up data directly into a designated computer (see Resources) for processing, with double entry to verify the accuracy of data. All computer data will be password protected and maintained on a computer in the PI's locked office, and participant information will be identified only by study identification numbers. Participants will be asked to provide contact information for follow-up purposes only. For added security, participant contact information will be removed from the original database and stored in a separate location. A separate record linking participant names and contact information to participant ID numbers will be created and stored separately in a password protected file. Only project staff will have access to subject identities. All necessary steps will be taken to ensure subject confidentiality. All information will be archived for 7 years from completion of the project.

10.1.1. *Research Materials Handling*: All materials derived from the study will be handled only by study personnel during collection, storage, and in subsequent data analysis. The PI is experienced in conducting clinical studies and is fully aware of the need for anonymity, privacy and security, and maintains strict surveillance of the office environment. No incidents of violation of patient confidentiality have occurred in the previous studies in which the PI has participated.

10.1.2. *Handling of Study Treatment Audio Recordings*. We will use a password protected, secure file transfer protocol to maintain security of audio recordings when they are sent to fidelity monitors. This protocol includes the following features:

- Access to the data entry system is restricted to SSL (128 bit) encrypted connections only. If FTP or application-driven email is implemented we will implement encryption for those services as required.
- The password is hashed (one-way-encrypted) using MD5 encoding so it is nearly impossible to reverse engineer the hash and get a person's password. Password length is restricted to a minimum length of eight characters to make brute force hacking difficult.
- After three failures the user is locked out for a period of one hour, making brute force hacking difficult.
- Access is logged making hacking attempts easier to discover.
- The servers are behind a locked door with a combination lock. Access is restricted to key IT personnel in the Data & Statistics Core. The servers are on a backup power supply so that only extended power outages would cause problems. All servers are backed up on a nightly basis. The data

on each of the database servers is backed up nightly and the backups are saved for a period of ten days.

- Server logs are monitored for system problems and hacking attempts.
- All critical data transactions are maintained in a series of audit and archive tables.
- Firewalls: The University provides a series of CISCO firewalls and switched routers to provide the first line of campus security. Additionally, an external agency is responsible for monitoring firewall activity and event logs to provide an independent analysis of intrusion attempts and trends. Network Design: The University has subdivided its network into Virtual Local Area Networks (VLANS). A user must be physically mapped into a VLAN before any resources may be accessed.
- Each user is required to maintain a unique username and password. Once the user logs in and the system verifies they are a valid user, a "server session object" is created. This "object" monitors, tracks, and logs the user's activity while logged in, and disconnects the user if they have not had any activity within a specified time frame.

10.1.3. Data Monitoring Procedures: There will be weekly meetings of the research team led by the PI for monitoring progress, reviewing recruitment goals, evaluation of lapsed participation, and review of missing data. Plans for remediation of missing information and lapsed participation will be formulated by the PI at those times as needed.

10.1.4. Data Risk Reporting: The Study Coordinator will be responsible for compiling recruitment numbers, completing assessments, maintaining databases, and identifying missing data and lapsed participation. They will report this information to the PI at weekly meetings.

10.1.5. Data Integrity: Data integrity will be monitored closely. All personnel will have undergone Good Clinical Practice (GCP) training prior to study initiation and will be human subjects/ethics certified. Study records will be entered into the password protected, secure study database (backed up on a secure server). Files and database entries will be audited by an independent research coordinator from the Social Research Institute at University of Utah. Audits will occur on approximately 10% of all study files and will be conducted yearly during the course of the study as part of overall quality assurance.

11. SAFETY AND ADVERSE EVENTS

11.1 Background

This section describes the requirements and processes for reporting adverse events (AE), serious AE (SAE) and unanticipated problems to the Central IRB, National Institute on Drug Abuse (NIDA), and DSMB. It incorporates guidelines provided by the Office of Human Research Protections (OHRP) of the Department of Health and Human Services (DHHS) and Food and Drug Administration (FDA) reporting requirements. NIH is obligated to ensure that researchers comply with their approved reporting procedures. Clinical trial investigators funded by NIDA are obligated under federal regulations to appropriately inform the Institute of adverse events and unanticipated problems, and NIDA is required to ensure that the appropriate procedures are in place to support this reporting.

11.2 Definitions

Adverse Event: Because 45 CFR 46 does not provide a specific definition for an adverse event (AE), the definition of an AE will conform to the International Conference on Harmonization (ICH) Good Clinical Practice (GCP) Guidelines. The same definition is used by the U.S. Food and Drug Administration (FDA) except that "drug" is typically used instead of the term "intervention." An AE is defined as any untoward medical occurrence in a patient or clinical investigation subject administered an intervention and which does not necessarily have a causal relationship with this treatment. An AE can therefore be any unfavorable and unintended sign (including an abnormal laboratory finding), symptom, or disease temporally associated with the use of an investigational intervention, whether or not related to the intervention.

Serious Adverse Event (SAE): Any AE that:

- Results in death;
- Is life threatening, or places the participant at immediate risk of death from the event as it occurred;
- Requires or prolongs hospitalization;
- Causes persistent or significant disability or incapacity;
- Results in congenital anomalies or birth defects;
- Is another condition, which the investigators judge to represent significant hazards.

Unanticipated Problem: any incident, experience, or outcome that meets all of the following criteria:

- Unexpected, in terms of nature, severity, or frequency, given (a) the research procedures that are described in the protocol-related documents, such as the IRB-approved research protocol and informed consent document; and (b) the characteristics of the study population;
- Related or possibly related to participation in the research; in this guidance document, possibly related means there is a reasonable possibility that the incident, experience, or outcome may have been caused by the procedures involved in the research;
- Suggests that the research places participants or others at a greater risk of harm (including physical, psychological, economic, or social harm) than was previously known or recognized.

Adverse Event Reporting Period: The study period during which adverse events must be reported is normally defined as the period from the initiation of any study procedures to the end of the study treatment follow-up.

Preexisting Condition: A preexisting condition is one that is present at the time of providing the consent for the study. A preexisting condition is considered an adverse event if the frequency, intensity, or the character of the condition worsens during the study period.

11.3 Responsibilities

Investigators conducting clinical research are responsible for:

- Assurance that their procedures are conducted in compliance with these guidelines.
- Submission of IRB-approved protocol to the NIDA program office. A DSMP that is commensurate with the study risks and reflects these guidelines will also be submitted to NIDA program staff.
- DSMP describes the plans for adverse events, serious adverse events, and unanticipated problems commensurate with the nature and complexity of the study.
  - Recipients of Serious Adverse Event and Unanticipated Problem reports must include the IRBs, DSMB, and NIDA.
  - Adherence to the DSMP with respect to timely submission of adverse events, serious adverse events, and unanticipated problems.

11.4 Ascertainment of AE, Unanticipated Problems (UP), and SAE

The PI will record all reportable events with start dates occurring any time after informed consent is obtained until 7 (for non-serious AEs) or 30 days (for SAEs) after the last day of study participation. At each study visit, the investigator will inquire about the occurrence of AE/SAEs since the last visit. Events will be followed for outcome information until resolution or stabilization.

Unanticipated problems will be recorded in the data collection system throughout the study.

Prior to the study, volunteers will undergo physical exams to rule out the presence of undetected medical or psychological/psychiatric problems. During the performance of the studies, the volunteers will be monitored at all times by research personnel associated with the project (research assistant, technologists) or the investigators themselves. All the volunteers will have direct access to the phone numbers and pagers of the study coordinator and the responsible clinician, as well as an additional 24-hour contact number (emergency room services). These numbers are additionally included in the consent form provided to the volunteers. They will be informed of all the possible adverse events that could be encountered during the study. They will be encouraged to contact the investigators if they notice any adverse events. The investigators have ample prior experience in the utilization of the protocols utilized in the studies.

This trial involves low-risk interventions for a population already at relatively low risk of serious clinical events based on the underlying conditions (e.g., chronic musculoskeletal pain conditions are chronic, not progressive, and typically involve low medical risk). Although a life-threatening event or long-term life-compromising effect due to the study treatments is extremely unlikely, we will implement several monitoring approaches to ensure patient safety. One is a “top-down” approach, in which after a “medically /psychologically serious event” occurs, we will investigate whether the event is indeed an adverse effect due to our protocol.

The other is a “bottom-up” approach. All participants will be asked to record any adverse events in their diaries and to report them to the therapists and the study coordinator. In addition, they will be asked about their experiences weekly at the treatment sessions. Participants will also be strongly encouraged to discuss with the research staff anything they feel to be an adverse event or other issues that are problematic for them about the study. Adverse events will be recorded and reported to the PI, co-investigators, and the University of Utah IRB. In the event of a report of a possible adverse event, the participant will be seen as soon as possible by the PI or Clinical Research Coordinator for an evaluation. The level of care will be determined and the participant referred appropriately for care, which may involve the resources of the University Primary Care Clinics, the University of Utah Hospital, or the University Neuropsychiatric Institute. The study clinicians will not provide medical care directly but will provide advice with regard to the appropriate type of medical care, and helping the participant obtain that level of care. If the PIs, treating physician, or the participant believes that a serious adverse event resulting from the study has occurred, the PI will immediately notify the University of Utah IRB and NIDA Program Officer. Further involvement in the study by that participant may be terminated.

Hence, several mechanisms will be in place to identify participants who may be experiencing adverse effects during the study: 1) communication between participants and the research staff; 2) direct, weekly observation of participant’s health status by the therapists and during physician monitoring at the follow-up sessions; and 3) verbal inquiry concerning potential adverse affects through weekly review by the therapists.

11.5 Safety Personnel

Medical Safety Monitor (MSM): The MSM will coordinate reporting of the safety data. The MSM will monitor and evaluate all collected UPs and SAEs by regular review/monitoring of reports of monitored SAEs or UP reported directly by the PI/personnel. The MSM will have a critical role, ensuring that all participants receive the highest quality care and that safety concerns are foremost in the conduct of this trial. The MSM may suggest measures to the PI to improve monitoring or prevent risk to participants. Upon approval by the DSMB, these modifications may be submitted as protocol modifications to the IRB. The MSM will review quarterly reports of UPs and SAEs generated by the DCC (in total, but not segregated by treatment assignment) and submit these to the SO. Once approved by the SO, the SAE reports will be sent by the MSM to the DSMB, and NIDA for distribution to the PI for filing with the local IRB. The summary will include descriptive information about SAEs reported and indicate both the number of participants enrolled as well as those experiencing a given SAE.

11.6 Reporting of Unanticipated Problems and Serious Adverse Events

A summary report of the UP and SAE collected by all methods of ascertainment will be prepared by the MSM for review by the DSMB, NIDA, and IRB in accordance with the Safety Monitoring Plan.

5. ETHICAL AND REGULATORY CONSIDERATIONS

12.1 Good Clinical Practice Statement

The trial will be conducted in accordance with the ethical principles that have their origin in the Declaration of Helsinki, and that are consistent with the ICH guidelines for Good Clinical Practice (GCP) and all federal as well as local regulatory requirements.

12.2 Informed Consent

The principles of informed consent are described in ICH guidelines for GCP (ICH E6[R1]).

As noted previously in the screening procedures outlined above, participants identified by EHR data pull will be contacted by phone if they do not otherwise opt out. Following phone screening, informed written consent will be obtained by trained study personnel with all potential participants receiving a general description of the study, including the study specific baseline evaluations, along with the study specific evaluations.

12.3 Institutional Review Board

The University of Utah IRB will be responsible for review and approval of the protocol, any amendments, the informed consent form (ICF) and any other materials provided to participants. The IRB will be informed of any event likely to affect the safety of the participant or the continued conduct of the trial. Records of IRB review and approval of study protocol, ICF, and amendments will be kept by the site PI.

12.4 Risks to Human Subjects

Risks associated with MORE or SG activity are deemed to be minimal. There is no evidence that mindfulness or supportive group psychotherapy activities worsen the symptoms of chronic pain or cause complications with opioid therapy. Our assessment of minimal risk does not, however, preclude the possibility that adverse events, and serious adverse events could occur. Therefore, a comprehensive data safety monitoring plan is outlined in the following sections. Authority for monitoring the safety of the protocol will reside in an independent DSMB responsible for holding the PI accountable for data quality and completeness, and assessing the ongoing safety of the trial participants through periodic meetings/review. Ongoing participant safety monitoring is the responsibility of the PI who will report adverse events to a Medical Safety Monitor appointed by the PI. The Medical Safety Monitor has the authority to directly report concerns to the DSMB if they arise.

There are potential risks associated with data collection and information management. These include inadvertent disclosure of personal health information or research data collected. Every effort will be made to inform the participant of this potential and minimize the risks as outlined below.

12.5.1 Protection Against Risks

<u>Minimizing Risks</u>: The study protocol will be implemented only after the PI, and study coordinator have undergone rigorous protocol training. The training and monitoring of performance in accordance with the study protocol will be the responsibility of the PI. Risk mitigation will occur through the following mechanisms.

<u>*Therapists*</u>: Licensed therapists with at least 5 years of mindfulness or addictions treatment experience working with persons with co-occurring chronic pain and opioid misuse will be hired and trained by the PI, who developed and implemented a 14-hour MORE training workshop and support group training protocols for three separate trials of MORE. Training will involve didactic and experiential instruction, with the PI monitoring and providing real-time feedback on mock therapist-client interactions through a one-way mirror and “bug in the ear” technology in the observation suite of the PI’s lab. Treatment sessions will be audio-recorded and reviewed by the PI and Co-Is to monitor fidelity, with feedback delivered to therapists in weekly supervision to maintain intervention integrity. Treatment groups will be comprised of 8-10 individuals, with sessions held in a

private group room at the University of Utah Primary Care Clinics.

The PI has extensive training and experience in mindfulness meditation, mindfulness instruction, and psychotherapy. He developed the MORE intervention and delivered it to several groups of substance dependent inpatients in the context of three randomized controlled pilot trials. He has also implemented a modified version of MORE in one-on-one individual therapy sessions in an outpatient integrative medicine clinic from 2007 – 2010. The PI (Eric Garland) is a licensed clinical social worker with 10+ years of experience who has treated hundreds of persons with co-occurring substance use, mental health, and medical disorders. Dr. Garland will provide consultation as necessary with regard to clinical or safety issues that may arise during the context of the study. Dr. Garland's review of treatment sessions will provide a further layer of protection against risk, as he will make recommendations and provide guidance with regard to managing difficult clinical issues that may arise during group sessions. Secure handling and file transfer protocol for study treatment audio-recordings is described below.

Medical Emergencies: The therapists will conduct the MORE and SG sessions in a group therapy room at the University of Utah Primary Care Clinics. This room is adjacent to clinical offices staffed by physicians, nurses, and clinical social workers, and all sessions will be held at times when medical personnel are present. All such persons are available and trained under guidelines for management of medical emergencies.

Adverse Event Reporting: All participants will be asked to record any adverse events in their diaries and to report them to the therapists and the study coordinator. In addition, they will be asked about their experiences weekly at the treatment sessions. Participants will also be strongly encouraged to discuss with the research staff anything they feel to be an adverse event or other issues that are problematic for them about the study. Adverse events will be recorded and reported to the PI, Co-investigators, and the University of Utah IRB. Hence, several mechanisms will be in place to identify participants who may be experiencing adverse effects during the study: 1) communication between participants and the research staff; 2) direct, weekly observation of participant's health status by the therapists and during study staff at the follow-up sessions; and 3) verbal inquiry concerning potential adverse affects through weekly review by the therapists.

Response to Adverse Events: In the event of a report of a possible adverse event, the participant will be seen as soon as possible by the PI or the MSM for an evaluation. The level of care will be determined and the participant referred appropriately for care, which may involve the resources of the University Primary Care Clinics, the University of Utah Hospital, or the University Neuropsychiatric Institute. Other than in the case of stress or craving resulting from the measurement protocol as described above, study personnel beyond the PI, or MSM will not provide medical care directly but will provide advice with regard to the appropriate type of medical care, and helping the participant obtain that level of care. If the PI, treating physician, or the participant believes that a serious adverse event resulting from the study has occurred, the PI will immediately notify the University of Utah IRB and NIH Program Officer. Further involvement in the study by that participant may be terminated.

12.5.2. Potential Benefits of the Proposed Research to Participants and Others

The potential benefits of the research to the participants are significant. Their symptoms of chronic pain, opioid misuse, craving, or stress may improve significantly as a result of being part of the study. All participants will undergo assessments of chronic pain and stress symptoms, coping strategies and psychological factors that may influence the course of their condition. Insights from participation in mindfulness training and support group activities may be useful to participants to improve their health. Research suggests that mindfulness training may be useful as an adjunctive treatment for a number of disorders associated with chronic pain that may be present in members of the study cohort. Participants in both groups may experience greater clinical improvement than they might otherwise experience with usual medical care alone. The process of participating in the MORE or the SG could enhance self-efficacy and other benefits of being part of a social support group. Participants may learn improved methods of managing chronic pain from each other's experience. Therefore, it is expected that both groups may experience significant benefit from participating in the research study.

The potential benefits to other individuals with chronic pain at risk for opioid misuse are also significant. The results of this research may provide credible evidence about the effects of MORE and SG participation as treatments for chronic pain and opioid misuse, and whether they may be usefully integrated with conventional medical. Any evidence derived from this research suggesting that MORE improves chronic pain and opioid misuse may help other patients with these issues obtain relief through this approach. Information derived from this research suggesting that MORE may not be useful for chronic pain and opioid misuse will help patients and their health-care providers focus on treatment modalities that are more effective.

Other benefits apply to future research efforts by clarifying methodological issues, such as participant accrual, research design of control interventions, and effect size estimation.

<u>Participant compensation.</u> Participants will be compensated in a pro-rated fashion up to $320 for completing all study assessments and visits. Participants will be paid $30 for the pre-assessment, and $50 for the post-assessment, $30 for the 3 follow-up assessments, plus $10 for each treatment session attended (to defray transportation costs [gas, parking, etc.] to the sessions), plus $40 for completing the EMAs, and a potential $30 bonus for completing all study activities.

12.5.3. Importance of the Knowledge to be Gained.
It is likely that the results of this project will provide two important pieces of information: The first relates to the feasibility of doing larger studies using this methodology including issues of: integration of MORE with conventional medical care; the approach used in the specific application of MORE for chronic pain and opioid misuse in primary care; use of a support group control; and statistical approaches as described earlier.
The second piece of information relates to evidence for or against the use of MORE in combination with usual medical care as a treatment for chronic pain, opioid craving, and opioid misuse behaviors in primary care.

Results supporting the use of MORE will lead to expanded comparative effectiveness studies and implementation science to more clearly define the magnitude of MORE's benefits and the scope of its clinical uses. If the use of MORE for chronic pain, opioid craving, and opioid misuse behaviors is supported by future studies, the prescribing of MORE could potentially diminish the side effects and high costs of extant pharmacological treatments for the targeted condition.

Additionally, a positive, well-conceived and conducted clinical trial could open the door for detailed investigation of the mechanism of action of MORE specifically, and mindfulness meditation, in general. Knowledge derived from this and follow-up studies could further enhance our understanding of human biology from the standpoint of health maintenance and healing in ways that have not been fully appreciated or well conceptualized to date.

If the results of this and future studies suggest that MORE is not effective and does not improve the clinical course in patients with chronic pain and opioid misuse in primary care, patients with these conditions and their healthcare providers can focus their efforts on other potentially useful therapies.

12.6. Inclusion of Women and Minorities.

Inclusion of Women. Women and men will be recruited for this study. Women are over-represented in samples of chronic pain patients; indeed, chronic pain is a condition that affects women to a disproportionately greater extent than men. Based on our prior experience delivering MORE and support groups in our pilot randomized controlled trial, the interventions are acceptable in mixed-gender groups. We will stratify our random assignment by gender to ensure equal representation in the groups.

Inclusion of Ethnic Minorities. All patients with chronic pain diagnoses receiving opioid therapy will be eligible to participate irrespective of race or ethnic background. There is no reason to expect differential accrual rates by ethnic background, and we expect the distribution of the study subjects to reflect the distribution among the eligible study population seen at the University of Utah Primary Care clinics (which, due to the demographics of Salt Lake City, will be predominately white). Ethnic background/race did not significantly

predict treatment participation or outcomes in our prior trials of MORE. It is appropriate to make the study available and accessible to all eligible patients irrespective of their ethnic background and to encourage and facilitate the participation of all eligible patients.

13. STUDY DOCUMENTS

13.1 Retention of Records

The PI will retain study-related documents for at least five years following the completion or discontinuation of the study. If the PI's personal situation is such that such archiving can no longer be ensured, the relevant records will be transferred to a central secure location for storage.

14. PUBLICATION AND DISSEMINATION POLICY

The PI in discussion with the Co-Is will set the rules for publication of the data and authorship in conformity with NIH policies. The clinical outcomes of the trial will be published first in accordance with Specific Aim 1, followed by a series of publications on the mechanistic trial outcomes, in accordance with Specific Aims 2 and 3.

14.1 Dissemination

The findings of this trial have important implications for public health and policy, requiring wide dissemination; additionally, several aspects of this trial's approach and implementation will shape clinical practice and form the basis for dissemination after the trial. Should MORE prove to be efficacious, the PI will provide clinical training in MORE to health care providers (e.g., social workers, psychologists, nurses, and physicians) in community settings.

TITLE: Mindfulness-Oriented Recovery Enhancement for Opioid Misuse and Chronic Pain in Primary Care: A Randomized Controlled Trial

**PI:**	**ERIC GARLAND, PHD**
**AGENCIES:**	**NATIONAL INSTITUTE ON DRUG ABUSE**
**NIDA GRANT NUMBER:**	**R01DA042033**
**APPLICANT ORGANIZATION:**	**UNIVERSITY OF UTAH**
**DATE OF ORIGINAL IRB APPROVAL:**	**03/25/2015**
**VERSION:**	**1.0**

Title	**Mindfulness-Oriented Recovery Enhancement for Opioid Misuse and Chronic Pain in Primary Care: A Randomized Controlled Trial**
Study Design	The design is a parallel group superiority trial. The unit of randomization is the individual patient.
Study Duration	5 years.
Trial Sites	University of Utah Health – Community Physicians Group.
Objective	Conduct a randomized controlled trial to determine the efficacy of a Mindfulness-Oriented Recovery Enhancement intervention for opioid misuse and chronic pain in primary care.
Number of Subjects	The target sample size is 260 participants enrolled (200 + oversampling by 30% for attrition) to provide 80% probability that a two-sided 95% confidence interval will have a margin (half-width) less than +/- .18 standard deviation for the between-groups difference in the study outcomes.
Main Inclusion Criteria	Age ≥18 with a chronic pain-related diagnosis, reporting current pain ≥3 on a 0-10 scale, currently taking prescription opioids for ≥3 months, and surpassed a validated cutpoint for opioid misuse on the Current Opioid Misuse Measure (COMM).
Intervention	Mindfulness-Oriented Recovery Enhancement (MORE) is a group behavioral intervention that unites mindfulness training, cognitive reappraisal, and positive psychological principles into an integrative intervention strategy targeting mechanisms of pain and opioid misuse.
Duration of Intervention	8 weeks.
Primary Outcome	The primary outcome is opioid misuse measured by the Drug Misuse Index, operationalized as scores on the COMM and triangulated by blinded clinical interview and urine drug screen. The co-primary outcome is chronic pain symptomology, operationalized as pain severity and functional interference scores on the Brief Pain Inventory (BPI).

Primary Analysis	Effects of treatment on the DMI will be analyzed with generalized mixed effects models, and effects on the BPI will be analyzed with mixed effect ANCOVA models, adjusting for baseline levels.
Secondary Outcomes	Opioid dose, psychological distress, and opioid craving.
Interim Analysis	Interim monitoring will focus on patient accrual, baseline comparability of treatment groups, protocol adherence, data completeness and quality, and safety. No interim outcome analysis is planned.

1. INTRODUCTION

This document is a protocol for a human research study to be conducted according to US and international standards of Good Clinical Practice (FDA Title 21 part 312 and International Conference on Harmonization guidelines), applicable government regulations, and institutional research policies and procedures.

2. STUDY OBJECTIVES

The proposed parallel-group randomized clinical trial will determine the efficacy of Mindfulness-Oriented Recovery Enhancement (MORE) as a treatment for opioid misuse and chronic pain in a primary care setting. Design features of this study draw upon strengths of Stage 2 and Stage 3 research per the NIH Stage Model,[1] maximizing internal validity, intervention potency, and fidelity assurance with research therapists while maximizing external validity by delivering the intervention in community clinics – the setting where most chronic pain patients seek care.[2]

MORE integrates training in mindfulness, reappraisal, and savoring of natural rewards to enhance hedonic regulation and target the risk chain at the point where maladaptive emotion-cognition interactions link chronic pain to opioid misuse. MORE is innovative in that it aims to modify associative learning mechanisms dysregulated by the allostatic effects of opioid misuse on brain reward systems via strengthening top-down cognitive control to restructure bottom-up reward learning from valuation of drug reward to natural reward – something that no other behavioral intervention for opioid misuse has been designed to do.

MORE was originally tested in a university setting as an intervention for opioid misuse among patients prescribed opioids for chronic pain in an R03-funded, Stage I pilot randomized controlled trial (RCT). Results demonstrated that MORE significantly decreased opioid misuse and opioid craving, as well as reduced chronic pain severity and functional impairment, relative to a support group (SG) control.[3] Notably, MORE significantly reduced opioid cue-reactivity and enhanced neurophysiological responsiveness to natural reward stimuli relative to the SG control, providing compelling preliminary evidence that MORE may restructure reward processing.[4,5]

The Specific Aims of this study are as follows:

AIM 1. To conduct a RCT of the Mindfulness-Oriented Recovery Enhancement (MORE) intervention for co-occurring aberrant drug-related behaviors and chronic pain in primary care.
We will compare the therapeutic impact of MORE and a conventional supportive group (SG) psychotherapy active control condition on clinical outcomes germane to opioid misuse and chronic pain. **Hypotheses**: Opioid misusing patients] assigned to MORE, as compared to SG participants, will evidence decreased opioid misuse and pain (*PRIMARY OUTCOMES*), as well as reduced opioid craving, opioid dosing, and psychological distress (*SECONDARY OUTCOMES*) from pre- to post-treatment through 9-month follow-up.

AIM 2. To test and quantify the degree to which MORE's impact on aberrant drug-related behaviors and pain is mediated by proactive control over emotion-cognition interactions (top-down mechanism).
Hypotheses: The impact of MORE on opioid misuse and pain will be mediated by improvements in: **a)** attentional disengagement from opioid-related cues (reduced attentional bias); **b)** emotion regulation**;** and **c)** cognitive coping (e.g., reinterpretation of pain as innocuous sensory information).

AIM 3. To test and quantify the degree to which MORE's impact on aberrant drug-related behaviors is mediated by restructuring of reward processing (bottom-up mechanism).
Hypotheses: The impact of MORE on opioid misuse will be mediated by restructured reward processing, as indicated by a shift in the relative responsiveness to opioid and natural reward cues.

3. BACKGROUND

Prescription opioid misuse among chronic pain patients is an emerging public health threat that is being addressed with heightened urgency at both clinical and policy levels. Though opioid analgesic therapy can manage chronic pain, it may confer with significant health risks, including dependence, overdose, and misuse. Opioid misuse is evidenced by aberrant drug-related behaviors such as unauthorized dose escalation or use of prescribed opioids to self-medicate negative emotions that exacerbate craving.[6] Research on treatments for opioid misuse among chronic pain patients is scant; according to a 2015 NIH-AHRQ systematic review of long-term opioid therapy for chronic pain, “no study evaluated the effectiveness of risk mitigation strategies for improving outcomes related to overdose, addiction, abuse, or misuse.”[7] Extant therapies may have limited efficacy because they fail to directly target cognitive-affective neural circuits that govern hedonic regulatory responses elicited by pain-, opioid-, and reward-related stimuli.[8] To address this need, the PI translated mechanistic findings from behavioral neuroscience into an innovative treatment for prescription opioid misuse, called **Mindfulness-Oriented Recovery Enhancement** (MORE). MORE integrates training in mindfulness, reappraisal, and savoring of natural rewards to enhance hedonic regulation and target the risk chain at the point where maladaptive emotion-cognition interactions link chronic pain to opioid misuse. MORE is innovative in that it aims to modify associative learning mechanisms dysregulated by the allostatic effects of opioid misuse on brain reward systems via strengthening **top-down cognitive control to restructure bottom-up reward learning from valuation of drug reward to natural reward** – something that no other behavioral intervention for opioid misuse has been designed to do. Accordingly, this project funded by the National Institute on Drug Abuse (NIDA) is a randomized clinical trial (RCT) of MORE to reduce opioid misuse and chronic pain symptoms among patients receiving long-term opioid therapy in primary care.

4. STUDY DESIGN

A two-arm parallel RCT design with an active control group will minimize internal threats to validity. Mindfulness-Oriented Recovery Enhancement (MORE; n=130) and the Supportive Group Psychotherapy control (SG; n=130) will be equated for time spent in treatment. Assessors will be blind to treatment condition. Target sample sizes were determined via power and precision analyses. The unit of randomization is the individual patient. The co-primary outcomes are opioid misuse and chronic pain symptoms. Participants will complete a clinical assessment battery at pre- and post-treatment, and at 3-, 6-, and 9-month follow-ups. Participants will complete a psychophysiological assessment protocol at pre- and post-treatment. Participants will complete ecological momentary assessments (EMAs) of symptoms, skill practice, and opioid use during treatment and 1 month afterward up to 3 times/day (morning, noon, and night).

4.3 Number of Subjects

Our estimates indicate that a target sample size of 260 participants will have 80% probability that a two-sided 95% confidence interval will have a margin (half-width) less than +/- .18 standard deviation for the between-groups difference in the study outcomes.

4.4 Subject Selection and Withdrawal

4.2.1 Inclusion and Exclusion Criteria

Table 1 lists the inclusion and exclusion criteria for screening patients for participation in the trial.

Table 1: Inclusion and Exclusion Criteria
**Inclusion Criteria**<br>• Age 18-60+<br>• Current back pain diagnosis as determined from ICD-9 codes in claims data (including but not limited to ICD-9 diagnoses 724.x, or 847.x) or current chronic pain diagnosis determined by physician assessment (including but not limited to ICD-9 diagnoses 338.0, 338.21, 338.22, 338.28, 338.29, 338.4)<br>• Current use of prescription opioid agonist or mixed agonist-antagonist analgesics for >90 days, and evidence of opioid misuse as indicated by the Current Opioid Misuse Measure<br>• Willingness to participate in study interventions and assessments
**Exclusion Criteria**<br>• Prior experience with Mindfulness-Based Stress Reduction, Mindfulness-Based Cognitive Therapy, or Mindfulness-Based Relapse Prevention<br>• Active suicidality, schizophrenia, psychotic disorder, and/or severe non-opioid substance use disorder<br>• Presence of clinically unstable systemic illness judged by physician to interfere with treatment

Because participants must be able to receive the MORE or SG treatments in English, it is not feasible to enroll participants who do not speak English.

4.4 Screening, Recruitment, and Enrollment

4.4.1 Screening

Patients aged 18 or older will be identified via the University of Utah Health system electronic health record (EHR) and subsequently screened. Patients will also be referred by their health care practitioner into the study. Screening will occur in clinic, over the phone, or in person at the Center on Mindfulness and Integrative Health Intervention Development (C-MIIND).

Screening questions assess the duration of prescribed opioid use, the type of opioid prescribed, the patient's pain condition, the patient's pain level while taking opioids, and whether or not the patient meets criteria for opioid misuse, according to the validated opioid misuse cutpoint on the COMM.[6]

4.4.2 Recruitment and Enrollment

Patients who screen positive will be informed about the study during an interview, during which the recruiter will confirm the absence of any exclusion criteria, review the purpose of the study, answer any questions and, after obtaining written informed consent, collect baseline data on: key demographic characteristics, clinical characteristics, and primary, secondary, and mechanistic outcomes. Recruitment staff will be kept blinded to randomization status of the patients and will be rigorously trained to reduce potential bias. Patients will remain blind to the nature of the experimental versus the control interventions by being told that the intention of the study is to compare two forms of group behavioral treatment for chronic pain, one based on mindfulness and the other based on social support. The informed consent process will not identify which condition (MORE or SG) is the experimental intervention and which is the control intervention.

4.4.3 Rescreening

Patients who screened out initially as not meeting study criteria for long-term opioid therapy (<90 days of opioid use) will be rescreened during subsequent visits. However, screen positive patients who had previously opted out or declined to participate will not be re-contacted.

4.4.4 Early Withdrawal of Participants

Because this trial is conducted via the "intent-to-treat" (ITT) framework, all participants will be analyzed in the group to which they were randomly assigned, regardless of whether they complete the intervention or are noncompliant. This preserves the effect of randomization. We plan to follow participants for the study outcomes even if they elect to stop the study intervention. If the participant elects full withdrawal from the study, this will be recorded and all contact with the participant will cease. Participants who are disruptive, or who develop active suicidality or psychosis, or medical conditions that would preclude completion of study procedures, may be withdrawn at any time by the discretion of the PI.

5. STUDY INTERVENTION

The **MORE arm** will participate in 8 weekly, 2-hour group sessions led by a Master's level clinical social worker. MORE sessions involve mindfulness training to disrupt aberrant drug-related behavior and reduce pain, cognitive reappraisal to decrease negative affect and regulate opioid craving, and savoring to augment natural reward processing and evoke positive emotion. MORE participants will be asked to engage in 15 minutes/day of skill practice at home, and complete daily EMA.

6. CONTROL INTERVENTION

The SG control arm will participate in 8 weekly, 2 hour SG sessions led by a Master's level clinical social worker, discussing topics and expressing emotions related to opioid misuse and chronic pain, in addition to completing daily 15 minute journaling sessions on pain and opioid-related themes and daily EMA. The SG format, which was adapted from the supportive group psychotherapy condition piloted in the PI's R03 and several of our prior RCTs. SG therapists will use a Rogerian client-centered therapy approach. We selected the SG as the control for the proposed R01 based on its success as a credible and active control condition in our preliminary studies of MORE,[3,9] which found no significant difference in ratings of intervention credibility between MORE and SG participants. Self-help and therapist-led SGs are standard care and among the most commonly available treatments for substance use disorders and chronic pain. The SG and MORE groups will be fixed and comprised of 8-10 individuals each.

7. OUTCOME MEASURES

7.2 Definition and Ascertainment of Outcomes

Outcome measures were informed by the Initiative on Methods, Measurement, and Pain Assessment in Clinical Trials (IMMPACT).[10] Outcome measures summarized below.

7.1.1. *<u>Primary outcomes (PRE/POST/FOLLOW-UP).</u>* Our pre-specified primary opioid misuse outcome in clinicaltrials.gov is a validated composite measure - the Drug Misuse Index (DMI).[11] Because there is no single way to identify patients who misuse opioids,[12] the DMI uses 3 levels of data to triangulate opioid misuse: self-reports on a structured questionnaire (i.e., the COMM); clinical assessment of opioid misuse—the Addiction Behaviors Checklist (ABC)[13]—rated by clinical staff (i.e., psychologists, social workers, and nurses) blinded to treatment assignment; and urine toxicology screens. The COMM consists of 17 items rated on a Likert scale (0=never, 4=very often) regarding how often in the past 30 days patients engaged in opioid misuse-related behaviors (e.g., used opioids in ways other than prescribed). COMM scores ≥9 and ABC scores ≥2 are considered positive. A positive rating from the urine screen is given when subjects test positive for illicit drugs or a non-prescribed opioid. Subjects with positive COMM scores will be given a positive DMI and classified as a misuser. If patients deny engaging in opioid misuse-related behavior (COMM scores <9), then positive ratings on both the urine screen and the ABC are needed for a positive DMI, because urine screens can be inaccurate due to variable drug metabolites, and clinician ratings may unreliable.[14,15] Otherwise, subjects will be given a negative DMI. Multiple studies support the validity of this DMI scoring method.[11,16–18] **Chronic pain symptoms** (pain severity and functional interference) will be assessed with the Brief Pain Inventory (BPI).[19]

7.1.2. *Secondary outcomes (PRE/POST/FOLLOW-UP).* **Opioid craving** will be measured with 2 items "How much do you want to take your opioids right now?" and "How strong of an urge do have to take opioids right now?" using 0-10 Numeric Rating Scales (NRS) delivered via EMA. Single item measures of craving have been shown to validly predict opioid misuse[16] and be sensitive to MORE treatment effects.[3] **Reduction in opioid dose** will be assessed by the Timeline Followback Procedure (TLFB) and triangulated by chart review of prescription history by the study coordinator.[20] Opioid dose will be converted to oral morphine-equivalent using standardized equianalgesic conversions. Subjects will also document daily opioid dose via EMA. **Psychological distress** will be measured with the Depression Anxiety Stress Scale-21 (DASS-21), a validated, reliable scale (α=.87-.94).[21]

7.1.3. Tertiary (mechanistic) outcomes *(PRE/POST)*.
Self-report mediators (PRE/POST). **Reinterpretation of pain sensations** as innocuous sensory information will be assessed with that validated subscale on the Coping Strategies Questionnaire (CSQ).[22] In the PI's R03, we found that effects of MORE on pain were mediated by increases in the Reinterpreting Pain Sensations of the CSQ.[3] **Mindfulness** will be measured with the Five Facet Mindfulness Questionnaire (FFMQ),[23] which the PI found in his R03 to mediate effects of MORE on pain.[3] **Reappraisal** will be measured with the positive reappraisal subscale of the Cognitive Emotion Regulation Questionnaire (CERQ)[149] which mediated the stress-reductive effects of mindfulness in an earlier study.[107] **Savoring** will be measured with the momentary savoring subscale from the Ways of Savoring Checklist (WOS), which mediated impact of daily positive events on positive mood in a prior study.[24]

Cognitive-affective tasks and psychophysiology (PRE/POST). See ancillary mechanistic study protocol.

7.1.4. Other tertiary (mechanistic) measures. **Trait positive and negative affect** will be measured with the PANAS. **Pain catastrophizing** will be measured with the CSQ. **Meaning in life** will be measured with the Meaning in Life Questionnaire. **Self-transcendence** will be measured with our own in-house instrument (in development). **Post-traumatic stress symptoms** will be measured with the Posttraumatic Symptom Checklist-Civilian Version (PCL-C). **Anhedonia** will be measured with the Snaith Hamilton Anhedonia and Pleasure Scale (SHAPS).

7.1.5. *EMA and process measures*. In symptom diaries 3 times/day, **opioid craving, pain intensity** and **unpleasantness,** and **positive and negative affect** will be assessed with 0-10 Numeric Rating Scales (NRS). Opioid dosing and amount of **mindfulness, reappraisal, and savoring skill practice** will be assessed at the end of the day. **State mindfulness** will be measured after each treatment session with the validated Toronto Mindfulness Questionnaire (TMS).[25]

8. STUDY PROCEDURES AND VISITS

8.3 Randomization

The trial has a two-group parallel randomized design. The unit of randomization is the individual patient. Following pre-intervention assessment, patients will be assigned in a 1:1 ratio to undergo MORE or a supportive group (SG) psychotherapy control condition.

8.4 Schedule of Events

The trial Schedule of Events is shown in **Table 3**.

Event	Screen	Pre -Tx	In Tx	Post-Tx	Follow-Up			
					1 Mo	3 Mo	6 Mo	9 Mo
Screening and Stratification Measures								
Informed Consent	X							
Demographics & Medical History	X							
MINI	X							
Primary and Secondary Outcome Measures								

BPI Pain severity and interference		X		X		X	X	X
DMI: ABC, COMM & Urine screen		X		X		X	X	X
Opioid dosing – TLFB and chart review		X		X		X	X	X
DASS Psychological distress		X		X		X	X	X
Opioid Craving NRS – EMA (see below)			X			X		
Mediators								
**Self-report mediators:** CSQ Reinterpretation FFMQ Mindfulness CERQ Reappraisal WOS Savoring		X		X		X	X	X
**Other tertiary measures:** PANAS Affect CSQ Pain Catastrophizing MLQ Meaning in Life SHAPS Anhedonia PCL PTSD		X		X		X	X	X
**Psychophysiological protocol**		X		X				
Intervention Process Measures and Ecological Momentary Assessments								
TMS State mindfulness (after each session)			X					
Opioid craving NRS - EMA			X			X		
Opioid dosing – EMA			X			X		
Affect NRS - EMA			X			X		
Pain Intensity & Unpleasantness NRS - EMA			X			X		
Therapeutic skill practice			X			X		

9. STATISTICAL ANALYSIS, SAMPLE SIZE AND POWER CALCULATIONS

9.6 Design Overview

This is a two-group, parallel randomized controlled superiority trial. The co-primary outcomes are opioid misuse (measured by the Drug Misuse Index [DMI]) and chronic pain symptoms (measured by the Brief Pain Inventory [BPI]). The target sample size is 260 participants before attrition to detect a small-moderate effect size reduction (Cohen's f = .20) in opioid misuse and pain with 80% power.

9.7 Subject Allocation to Treatment Arms

An electronic random number generator will randomize participants with simple random assignment in blocks of varying sizes (2 - 4) to preserve unpredictability of allocation, which will be concealed by opaque envelopes. To prevent bias and maintain allocation concealment, participants will not be allocated until the day of the first treatment session. Assessments will be conducted by project staff blinded to group assignment (which remained concealed throughout the study). To maintain blinding, the allocation list was inaccessible to project staff involved in assessment or treatment, and before each assessment, participants will be reminded to not reveal anything that would disclose their treatment assignment to study staff.

9.8 Sample Size

Power calculations are based on three complementary approaches. (a) *Univariate effect sizes*. The PI's R03 identified medium to large effect sizes on pain and opioid-related variables (Cohen's *d*=.50 - .84).[3] We used GPower 3.1 software to determine the smallest detectable effect for power of .80 against a range of sample sizes. The table indicates the smallest detectable effects for a treatment X time interaction term given different sample sizes, assuming a Type I error rate of .05 and varying correlations between repeated measures, which ranged from .34 -.56 per outcome in the R03. Cohen[26] defined *f*=0.10 and *f*=0.25 as small and medium effects, respectively. Thus our target N of 260 (N=200 + oversampling by 30% for attrition) will allow us to detect small-to-medium effects on continuous outcomes. Based on dichotomous clinical classification of opioid misuse data from the R03 (68% improvement for MORE versus 32% for SG), the proposed sample size will offer outstanding power (>.99) to detect differences of this magnitude on this outcome. (b) *Precision of parameter estimation*. Our target N will allow model parameters to be estimated with a high degree of precision. A per group sample size of N=130 provides 80% probability that a two-sided 95% confidence interval will have a margin (half-width) less than +/- .18 standard deviation. Thus the proposed sample will provide precision more than adequate for estimating small clinical effects with high confidence. (c) *Multivariate models*. Generally, structural equation power calculations can be based on Root Mean Square Error of Approximation (RMSEA) comparisons of nested models. The proposed sample provides, in a realistic testing scenario, greater than 80% power in a 1 *df* test (26 versus 25) of close fit comparing two models with RMSEA of .06 and .05 at α=.05. These calculations are conservative. Maximum likelihood techniques retain all observations at all times, and efficiently incorporate correlated observations to yield an Effective Sample Size that is quantifiably larger than the number of subjects. With fully specified repeated dimensions, the anticipated correlations will yield an effective sample size that is 40% larger, or roughly N=360.

Minimally detectable effect sizes (Cohen's *f*) for treatment X time interaction (pre-tx, post-tx, follow-up)

Correlation	N = 100	N = 110	N = 120	N = 130
0.25	0.16	0.15	0.14	0.14
0.50	0.13	0.12	0.12	0.11
0.75	0.09	0.09	0.08	0.08

9.9 Interim Monitoring

Interim monitoring will focus on patient accrual, baseline comparability of treatment groups, protocol adherence, data completeness and quality, and safety. No interim outcome analysis is planned.

Hypothetical treatment-response distributions

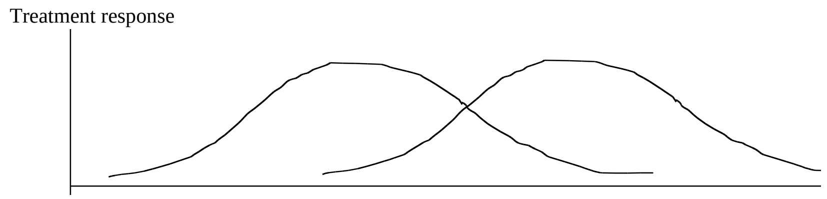

One of the purposes of the intermin monitoring is to provide the data-dependent guidance for the ongoing trial. Series of interim analyses can be conducted to determine whether the data-dependent stopping is called for, due to 1) the presence of overwhelming efficacy of the therapy(ies) in question or 2) "negative stopping" (terminate the project early because there appears to be no chance of a significant positive outcome). The basic premise underlying the former is to make the optimally effective treatment available to all patients as soon as possible. Generally, a sequence of interim analyses are conducted, using adjusted alpha levels (e.g., the Lan-DeMets approach with the O'Brien-Fleming spending function in order not to exceed an overall alpha of 5%). However, we propose that this assumption is problematic with our proposed study given our intent to investigate individual differences in treatment responses (i.e., moderators). Due to the large within-group variability, the presence of between-group differences does not provide specific information about who benefits from what treatment. For example, suppose that we obtain the data of treatment response in each of the 2 treatment groups as depicted in the Figure above. The distance between the two means may be sufficiently distant to show the significant group effect. However, when the within group variability is large, as in the case in many clinical trials in the area of chronic pain and addictions treatment, the presence of a significant between-groups difference does not tell us much about what treatment a clinician should provide given a patient. Thus, it would be premature to set up specific positive stopping roles based upon the interim group comparisons. Similarly, we will not consider the negative stopping. The rationale for this is that given the pre-specified power calculations based on structural equation models of moderated mediation

analyses, we will need to continue the trials until the end to assure the adequate level of statistical power to detect true difference. Thus, we propose that early termination of the project will occur only when the safety analysis of the project indicates as such (see below).

This study will be stopped prior to its completion if: (1) the intervention is associated with adverse effects that call into question the safety of the intervention; (2) any new information becomes available during the trial that necessitates stopping the trial; or (3) other situations occur that might warrant stopping the trial.

9.10 Statistical Analysis Plan

9.5.1. We will inspect scatter plots and descriptive statistics for study variables at pre-treatment, post-treatment, and the four follow-ups to determine the pattern of performance across time. Pearson correlation coefficients will be computed for associations among outcome measures and potential mediators. After data cleaning, we will examine the empirical distribution function of all variables, using the Kolmogorov-Smirnov GOF test and the Shapiro-Wilk normality test. If it is or can be transformed to a Gaussian distribution, parametric tests will be used for analysis. If not, non-parametric methods will be used.

9.5.2. In line with **Aim 1**, effects of treatment on opioid misuse as measured by DMI by 9-month follow-up will be analyzed with generalized linear mixed models with a binary outcome, with fixed effects consisting of a time factor and between-subjects treatment factor (MORE vs. SG). Because all patients will have a positive DMI at baseline (given the inclusion criterion of opioid misuse), models will include data from all post-treatment time points.Effects of treatment on continuous outcomes (will be evaluated using linear mixed models with fixed effects consisting of a time factor and between-subjects treatment factor (MORE vs. SG), and adjust for randomization imbalance by covarying baseline levels of the outcome. Our primary interest lies in the fixed effect of treatment, which with this model specification represents the average effect of treatment across all post-treatment measurement points. We will compare the change in log-likelihood (deviance) of the unrestricted maximum likelihood for the full model including interaction versus a constrained additive model. Random effects will incorporate correlated intercept and trend components, as in standard growth models, with additional dependence modeled, if needed and supported by the Bayesian Information Criterion, as autoregression in the repeated dimension. In addition, treatment group cohort and study therapist will also be modeled as random effects if indicated by the BIC and warranted by the ICC. If we identify a significant treatment X time interaction, we will investigate effects between time points through custom contrasts. Similar mixed models will be estimated for each of the proposed mediators, and mediational effects will be explored more fully in mediation models described below. Little's MCAR test will be used to determine if data are Missing Completely at Random (an ideal that is rarely met with longitudinal data). There is no definitive test for Missing at Random, which describes data that are random conditional on observed data. We will use maximum likelihood estimation (MLE) procedures to deal with missing data according to an intent-to-treat philosophy that is robust against common patterns of missing data (see also our didactic article[27]). MLE is based on all data observations; no values are deleted or imputed. We will conduct follow-ups on all clients regardless of whether or not they complete treatment. To control for false discovery in the three primary outcomes, we will compare the unadjusted p-values against Bonferroni-adjusted alpha =.05/3=.0167. For secondary outcomes, we conducted a global hypothesis test, at alpha=.05, of no treatment difference on any measure using a multivariate mixed effects linear model. Exact p-values will be reported for all comparisons.

9.5.3. To explore whether the cognitive, affective, and physiological mediators specified in **Aims 2 and 3** statistically mediate the effect of treatment on outcome variables, we will conduct structural equation path analyses according to established guidelines[28] by evaluating three regression paths: A) *'a'* path between treatment indicator and change in outcome (e.g., opioid misuse) from pre-treatment to follow-up; B) the *'b'* path between pre-post treatment change in the mediator (e.g., attentional bias) and pre-treatment to follow-up change in the outcome (e.g., opioid misuse); and C) the *'c'* path between treatment and pre-post treatment change in the mediator (e.g., attentional bias). We will test mediation by evaluating the significance of the joint product of the *a* and *b* paths, with bootstrapping[29] used to test the significance of the indirect effect. Our team developed a comprehensive analysis of mediation under possible moderation for clinical trials,[30] allowing for the possibility that treatment has changed relationships, not just levels, among variables - this will further guide analyses for Aims 2 and 3. Path models will be corrected for multiple comparisons. Mediational analyses will be published in a subsequent report following publication of the primary trial outcomes.

9.5.4. For EMA, we will use growth curve analysis[31] to examine treatment and homework practice effects on: A) trajectories of momentary craving, affect, and pain intensity/unpleasantness; B) relationships among these variables over time; and C) how trajectories in craving, pain, and affect predict variations in opioid dose in separate autoregressive latent trajectory (ALT)[32] models. Growth curve models will be specified with fixed effects consisting of a time factor and between-subjects treatment factor (MORE vs. SG), whereby the treatment X time interaction is the fixed effect of primary interest. As supported by the Bayesian Information Criterion, random effects will incorporate correlated intercept and trend components, as in standard growth models, with additional dependence modeled as autoregression in the repeated dimension. ALT models will estimate cross-lagged and auto-regressive effects, as well as latent intercept and growth factors, among opioid dose and other EMA variables, conditioned on a treatment group variable and process measures of homework practice (e.g., mindfulness, reappraisal, and savoring). EMA models will be corrected for multiple comparisons.

10. DATA MANAGEMENT

Data will be collected from the participants for research purposes only. Questionnaire data, clinical interview data, urine screen data, computerized cognitive task and psychophysiological data, and audio recorded materials will be the principal materials. They will be collected from the subjects at pre and post-intervention, as well as at each of the tri-monthly follow-ups, and weekly after the intervention sessions. These materials will be stored in the locked office of the PI prior to and during processing. Data management will include entry of pre-, post- and follow-up data directly into a designated computer (see Resources) for processing, with double entry to verify the accuracy of data. All computer data will be password protected and maintained on a computer in the PI's locked office, and participant information will be identified only by study identification numbers. Participants will be asked to provide contact information for follow-up purposes only. For added security, participant contact information will be removed from the original database and stored in a separate location. A separate record linking participant names and contact information to participant ID numbers will be created and stored separately in a password protected file. Only project staff will have access to subject identities. All necessary steps will be taken to ensure subject confidentiality. All information will be archived for 7 years from completion of the project.

10.1.1. *Research Materials Handling*: All materials derived from the study will be handled only by study personnel during collection, storage, and in subsequent data analysis. The PI is experienced in conducting clinical studies and is fully aware of the need for anonymity, privacy and security, and maintains strict surveillance of the office environment. No incidents of violation of patient confidentiality have occurred in the previous studies in which the PI has participated.

10.1.2. *Handling of Study Treatment Audio Recordings*. We will use a password protected, secure file transfer protocol to maintain security of audio recordings when they are sent to fidelity monitors. This protocol includes the following features:

- Access to the data entry system is restricted to SSL (128 bit) encrypted connections only. If FTP or application-driven email is implemented we will implement encryption for those services as required.
- The password is hashed (one-way-encrypted) using MD5 encoding so it is nearly impossible to reverse engineer the hash and get a person's password. Password length is restricted to a minimum length of eight characters to make brute force hacking difficult.
- After three failures the user is locked out for a period of one hour, making brute force hacking difficult.
- Access is logged making hacking attempts easier to discover.
- The servers are behind a locked door with a combination lock. Access is restricted to key IT personnel in the Data & Statistics Core. The servers are on a backup power supply so that only extended power outages would cause problems. All servers are backed up on a nightly basis. The data on each of the database servers is backed up nightly and the backups are saved for a period of ten days.
- Server logs are monitored for system problems and hacking attempts.
- All critical data transactions are maintained in a series of audit and archive tables.

- Firewalls: The University provides a series of CISCO firewalls and switched routers to provide the first line of campus security. Additionally, an external agency is responsible for monitoring firewall activity and event logs to provide an independent analysis of intrusion attempts and trends. Network Design: The University has subdivided its network into Virtual Local Area Networks (VLANS). A user must be physically mapped into a VLAN before any resources may be accessed.
- Each user is required to maintain a unique username and password. Once the user logs in and the system verifies they are a valid user, a “server session object” is created. This “object” monitors, tracks, and logs the user’s activity while logged in, and disconnects the user if they have not had any activity within a specified time frame.

10.1.3. Data Monitoring Procedures: There will be weekly meetings of the research team led by the PI for monitoring progress, reviewing recruitment goals, evaluation of lapsed participation, and review of missing data. Plans for remediation of missing information and lapsed participation will be formulated by the PI at those times as needed.

10.1.4. Data Risk Reporting: The Study Coordinator will be responsible for compiling recruitment numbers, completing assessments, maintaining databases, and identifying missing data and lapsed participation. They will report this information to the PI at weekly meetings.

10.1.5. Data Integrity: Data integrity will be monitored closely. All personnel will have undergone Good Clinical Practice (GCP) training prior to study initiation and will be human subjects/ethics certified. Study records will be entered into the password protected, secure study database (backed up on a secure server). Files and database entries will be audited by an independent research coordinator from the Social Research Institute at University of Utah. Audits will occur on approximately 10% of all study files and will be conducted yearly during the course of the study as part of overall quality assurance.

11. SAFETY AND ADVERSE EVENTS

11.5 Background

This section describes the requirements and processes for reporting adverse events (AE), serious AE (SAE) and unanticipated problems to the Central IRB, National Institute on Drug Abuse (NIDA), and DSMB. It incorporates guidelines provided by the Office of Human Research Protections (OHRP) of the Department of Health and Human Services (DHHS) and Food and Drug Administration (FDA) reporting requirements. NIH is obligated to ensure that researchers comply with their approved reporting procedures. Clinical trial investigators funded by NIDA are obligated under federal regulations to appropriately inform the Institute of adverse events and unanticipated problems, and NIDA is required to ensure that the appropriate procedures are in place to support this reporting.

11.6 Definitions

Adverse Event: Because 45 CFR 46 does not provide a specific definition for an adverse event (AE), the definition of an AE will conform to the International Conference on Harmonization (ICH) Good Clinical Practice (GCP) Guidelines. The same definition is used by the U.S. Food and Drug Administration (FDA) except that “drug” is typically used instead of the term “intervention.” An AE is defined as any untoward medical occurrence in a patient or clinical investigation subject administered an intervention and which does not necessarily have a causal relationship with this treatment. An AE can therefore be any unfavorable and unintended sign (including an abnormal laboratory finding), symptom, or disease temporally associated with the use of an investigational intervention, whether or not related to the intervention.

Serious Adverse Event (SAE): Any AE that:

- Results in death;
- Is life threatening, or places the participant at immediate risk of death from the event as it

occurred;

- Requires or prolongs hospitalization;
- Causes persistent or significant disability or incapacity;
- Results in congenital anomalies or birth defects;
- Is another condition, which the investigators judge to represent significant hazards.

Unanticipated Problem: any incident, experience, or outcome that meets all of the following criteria:

- Unexpected, in terms of nature, severity, or frequency, given (a) the research procedures that are described in the protocol-related documents, such as the IRB-approved research protocol and informed consent document; and (b) the characteristics of the study population;
- Related or possibly related to participation in the research; in this guidance document, possibly related means there is a reasonable possibility that the incident, experience, or outcome may have been caused by the procedures involved in the research;
- Suggests that the research places participants or others at a greater risk of harm (including physical, psychological, economic, or social harm) than was previously known or recognized.

Adverse Event Reporting Period: The study period during which adverse events must be reported is normally defined as the period from the initiation of any study procedures to the end of the study treatment follow-up.

Preexisting Condition: A preexisting condition is one that is present at the time of providing the consent for the study. A preexisting condition is considered an adverse event if the frequency, intensity, or the character of the condition worsens during the study period.

11.7 Responsibilities

Investigators conducting clinical research are responsible for:

- Assurance that their procedures are conducted in compliance with these guidelines.
- Submission of IRB-approved protocol to the NIDA program office. A DSMP that is commensurate with the study risks and reflects these guidelines will also be submitted to NIDA program staff.
- DSMP describes the plans for adverse events, serious adverse events, and unanticipated problems commensurate with the nature and complexity of the study.
  - Recipients of Serious Adverse Event and Unanticipated Problem reports must include the IRBs, DSMB, and NIDA.
  - Adherence to the DSMP with respect to timely submission of adverse events, serious adverse events, and unanticipated problems.

11.8 Ascertainment of AE, Unanticipated Problems (UP), and SAE

The PI will record all reportable events with start dates occurring any time after informed consent is obtained until 7 (for non-serious AEs) or 30 days (for SAEs) after the last day of study participation. At each study visit, the investigator will inquire about the occurrence of AE/SAEs since the last visit. Events will be followed for outcome information until resolution or stabilization.

Unanticipated problems will be recorded in the data collection system throughout the study.

Prior to the study, volunteers will undergo physical exams to rule out the presence of undetected medical or psychological/psychiatric problems. During the performance of the studies, the volunteers will be monitored at all times by research personnel associated with the project (research assistant, technologists) or the investigators themselves. All the volunteers will have direct access to the phone numbers and pagers of the study coordinator and the responsible clinician, as well as an additional 24-hour contact number (emergency

room services). These numbers are additionally included in the consent form provided to the volunteers. They will be informed of all the possible adverse events that could be encountered during the study. They will be encouraged to contact the investigators if they notice any adverse events. The investigators have ample prior experience in the utilization of the protocols utilized in the studies.

This trial involves low-risk interventions for a population already at relatively low risk of serious clinical events based on the underlying conditions (e.g., chronic musculoskeletal pain conditions are chronic, not progressive, and typically involve low medical risk). Although a life-threatening event or long-term life-compromising effect due to the study treatments is extremely unlikely, we will implement several monitoring approaches to ensure patient safety. One is a "top-down" approach, in which after a "medically /psychologically serious event" occurs, we will investigate whether the event is indeed an adverse effect due to our protocol.

The other is a "bottom-up" approach. All participants will be asked to record any adverse events in their diaries and to report them to the therapists and the study coordinator. In addition, they will be asked about their experiences weekly at the treatment sessions. Participants will also be strongly encouraged to discuss with the research staff anything they feel to be an adverse event or other issues that are problematic for them about the study. Adverse events will be recorded and reported to the PI, co-investigators, and the University of Utah IRB. In the event of a report of a possible adverse event, the participant will be seen as soon as possible by the PI or Clinical Research Coordinator for an evaluation. The level of care will be determined and the participant referred appropriately for care, which may involve the resources of the University Primary Care Clinics, the University of Utah Hospital, or the University Neuropsychiatric Institute. The study clinicians will not provide medical care directly but will provide advice with regard to the appropriate type of medical care, and helping the participant obtain that level of care. If the PIs, treating physician, or the participant believes that a serious adverse event resulting from the study has occurred, the PI will immediately notify the University of Utah IRB and NIDA Program Officer. Further involvement in the study by that participant may be terminated.

Hence, several mechanisms will be in place to identify participants who may be experiencing adverse effects during the study: 1) communication between participants and the research staff; 2) direct, weekly observation of participant's health status by the therapists and during physician monitoring at the follow-up sessions; and 3) verbal inquiry concerning potential adverse affects through weekly review by the therapists.

11.7 Safety Personnel

Medical Safety Monitor (MSM): The MSM will coordinate reporting of the safety data. The MSM will monitor and evaluate all collected UPs and SAEs by regular review/monitoring of reports of monitored SAEs or UP reported directly by the PI/personnel. The MSM will have a critical role, ensuring that all participants receive the highest quality care and that safety concerns are foremost in the conduct of this trial. The MSM may suggest measures to the PI to improve monitoring or prevent risk to participants. Upon approval by the DSMB, these modifications may be submitted as protocol modifications to the IRB. The MSM will review quarterly reports of UPs and SAEs generated by the DCC (in total, but not segregated by treatment assignment) and submit these to the SO. Once approved by the SO, the SAE reports will be sent by the MSM to the DSMB, and NIDA for distribution to the PI for filing with the local IRB. The summary will include descriptive information about SAEs reported and indicate both the number of participants enrolled as well as those experiencing a given SAE.

11.8 Reporting of Unanticipated Problems and Serious Adverse Events

A summary report of the UP and SAE collected by all methods of ascertainment will be prepared by the MSM for review by the DSMB, NIDA, and IRB in accordance with the Safety Monitoring Plan.

6. ETHICAL AND REGULATORY CONSIDERATIONS

12.5 Good Clinical Practice Statement

The trial will be conducted in accordance with the ethical principles that have their origin in the Declaration of Helsinki, and that are consistent with the ICH guidelines for Good Clinical Practice (GCP) and all federal

as well as local regulatory requirements.

12.6 Informed Consent

The principles of informed consent are described in ICH guidelines for GCP (ICH E6[R1]).

As noted previously in the screening procedures outlined above, participants identified by EHR data pull will be contacted by phone if they do not otherwise opt out. Following phone screening, informed written consent will be obtained by trained study personnel with all potential participants receiving a general description of the study, including the study specific baseline evaluations, along with the study specific evaluations.

12.7 Institutional Review Board

The University of Utah IRB will be responsible for review and approval of the protocol, any amendments, the informed consent form (ICF) and any other materials provided to participants. The IRB will be informed of any event likely to affect the safety of the participant or the continued conduct of the trial. Records of IRB review and approval of study protocol, ICF, and amendments will be kept by the site PI.

12.8 Risks to Human Subjects

Risks associated with MORE or SG activity are deemed to be minimal. There is no evidence that mindfulness or supportive group psychotherapy activities worsen the symptoms of chronic pain or cause complications with opioid therapy. Our assessment of minimal risk does not, however, preclude the possibility that adverse events, and serious adverse events could occur. Therefore, a comprehensive data safety monitoring plan is outlined in the following sections. Authority for monitoring the safety of the protocol will reside in an independent DSMB responsible for holding the PI accountable for data quality and completeness, and assessing the ongoing safety of the trial participants through periodic meetings/review. Ongoing participant safety monitoring is the responsibility of the PI who will report adverse events to a Medical Safety Monitor appointed by the PI. The Medical Safety Monitor has the authority to directly report concerns to the DSMB if they arise.

There are potential risks associated with data collection and information management. These include inadvertent disclosure of personal health information or research data collected. Every effort will be made to inform the participant of this potential and minimize the risks as outlined below.

12.5.2 Protection Against Risks

<u>Minimizing Risks</u>: The study protocol will be implemented only after the PI, and study coordinator have undergone rigorous protocol training. The training and monitoring of performance in accordance with the study protocol will be the responsibility of the PI. Risk mitigation will occur through the following mechanisms.

<u>*Therapists*</u>: Licensed therapists with at least 5 years of mindfulness or addictions treatment experience working with persons with co-occurring chronic pain and opioid misuse will be hired and trained by the PI, who developed and implemented a 14-hour MORE training workshop and support group training protocols for three separate trials of MORE. Training will involve didactic and experiential instruction, with the PI monitoring and providing real-time feedback on mock therapist-client interactions through a one-way mirror and “bug in the ear” technology in the observation suite of the PI’s lab. Treatment sessions will be audio-recorded and reviewed by the PI and Co-Is to monitor fidelity, with feedback delivered to therapists in weekly supervision to maintain intervention integrity. Treatment groups will be comprised of 8-10 individuals, with sessions held in a private group room at the University of Utah Primary Care Clinics.

The PI has extensive training and experience in mindfulness meditation, mindfulness instruction, and psychotherapy. He developed the MORE intervention and delivered it to several groups of substance dependent inpatients in the context of three randomized controlled pilot trials. He has also implemented a modified version of MORE in one-on-one individual therapy sessions in an outpatient integrative medicine

clinic from 2007 – 2010. The PI (Eric Garland) is a licensed clinical social worker with 10+ years of experience who has treated hundreds of persons with co-occurring substance use, mental health, and medical disorders. Dr. Garland will provide consultation as necessary with regard to clinical or safety issues that may arise during the context of the study. Dr. Garland's review of treatment sessions will provide a further layer of protection against risk, as he will make recommendations and provide guidance with regard to managing difficult clinical issues that may arise during group sessions. Secure handling and file transfer protocol for study treatment audio-recordings is described below.

Medical Emergencies: The therapists will conduct the MORE and SG sessions in a group therapy room at the University of Utah Primary Care Clinics. This room is adjacent to clinical offices staffed by physicians, nurses, and clinical social workers, and all sessions will be held at times when medical personnel are present. All such persons are available and trained under guidelines for management of medical emergencies.

Adverse Event Reporting: All participants will be asked to record any adverse events in their diaries and to report them to the therapists and the study coordinator. In addition, they will be asked about their experiences weekly at the treatment sessions. Participants will also be strongly encouraged to discuss with the research staff anything they feel to be an adverse event or other issues that are problematic for them about the study. Adverse events will be recorded and reported to the PI, Co-investigators, and the University of Utah IRB. Hence, several mechanisms will be in place to identify participants who may be experiencing adverse effects during the study: 1) communication between participants and the research staff; 2) direct, weekly observation of participant's health status by the therapists and during study staff at the follow-up sessions; and 3) verbal inquiry concerning potential adverse affects through weekly review by the therapists.

Response to Adverse Events: In the event of a report of a possible adverse event, the participant will be seen as soon as possible by the PI or the MSM for an evaluation. The level of care will be determined and the participant referred appropriately for care, which may involve the resources of the University Primary Care Clinics, the University of Utah Hospital, or the University Neuropsychiatric Institute. Other than in the case of stress or craving resulting from the measurement protocol as described above, study personnel beyond the PI, or MSM will not provide medical care directly but will provide advice with regard to the appropriate type of medical care, and helping the participant obtain that level of care. If the PI, treating physician, or the participant believes that a serious adverse event resulting from the study has occurred, the PI will immediately notify the University of Utah IRB and NIH Program Officer. Further involvement in the study by that participant may be terminated.

12.6.2. Potential Benefits of the Proposed Research to Participants and Others

The potential benefits of the research to the participants are significant. Their symptoms of chronic pain, opioid misuse, craving, or stress may improve significantly as a result of being part of the study. All participants will undergo assessments of chronic pain and stress symptoms, coping strategies and psychological factors that may influence the course of their condition. Insights from participation in mindfulness training and support group activities may be useful to participants to improve their health. Research suggests that mindfulness training may be useful as an adjunctive treatment for a number of disorders associated with chronic pain that may be present in members of the study cohort. Participants in both groups may experience greater clinical improvement than they might otherwise experience with usual medical care alone. The process of participating in the MORE or the SG could enhance self-efficacy and other benefits of being part of a social support group. Participants may learn improved methods of managing chronic pain from each other's experience. Therefore, it is expected that both groups may experience significant benefit from participating in the research study.

The potential benefits to other individuals with chronic pain at risk for opioid misuse are also significant. The results of this research may provide credible evidence about the effects of MORE and SG participation as treatments for chronic pain and opioid misuse, and whether they may be usefully integrated with conventional medical. Any evidence derived from this research suggesting that MORE improves chronic pain and opioid misuse may help other patients with these issues obtain relief through this approach. Information derived from this research suggesting that MORE may not be useful for chronic pain and opioid misuse will help patients and their health-care providers focus on treatment modalities that are more effective.

Other benefits apply to future research efforts by clarifying methodological issues, such as participant accrual, research design of control interventions, and effect size estimation.

Participant compensation. Participants will be compensated in a pro-rated fashion up to $300 for completing all study assessments and visits.

12.6.3. Importance of the Knowledge to be Gained.

It is likely that the results of this project will provide two important pieces of information: The first relates to the feasibility of doing larger studies using this methodology including issues of: integration of MORE with conventional medical care; the approach used in the specific application of MORE for chronic pain and opioid misuse in primary care; use of a support group control; and statistical approaches as described earlier.
The second piece of information relates to evidence for or against the use of MORE in combination with usual medical care as a treatment for chronic pain, opioid craving, and opioid misuse behaviors in primary care.

Results supporting the use of MORE will lead to expanded comparative effectiveness studies and implementation science to more clearly define the magnitude of MORE's benefits and the scope of its clinical uses. If the use of MORE for chronic pain, opioid craving, and opioid misuse behaviors is supported by future studies, the prescribing of MORE could potentially diminish the side effects and high costs of extant pharmacological treatments for the targeted condition.

Additionally, a positive, well-conceived and conducted clinical trial could open the door for detailed investigation of the mechanism of action of MORE specifically, and mindfulness meditation, in general. Knowledge derived from this and follow-up studies could further enhance our understanding of human biology from the standpoint of health maintenance and healing in ways that have not been fully appreciated or well conceptualized to date.

If the results of this and future studies suggest that MORE is not effective and does not improve the clinical course in patients with chronic pain and opioid misuse in primary care, patients with these conditions and their healthcare providers can focus their efforts on other potentially useful therapies.

12.7. Inclusion of Women and Minorities.

Inclusion of Women. Women and men will be recruited for this study. Women are over-represented in samples of chronic pain patients; indeed, chronic pain is a condition that affects women to a disproportionately greater extent than men. Based on our prior experience delivering MORE and support groups in our pilot randomized controlled trial, the interventions are acceptable in mixed-gender groups. We will stratify our random assignment by gender to ensure equal representation in the groups.
Inclusion of Ethnic Minorities. All patients with chronic pain diagnoses receiving opioid therapy will be eligible to participate irrespective of race or ethnic background. There is no reason to expect differential accrual rates by ethnic background, and we expect the distribution of the study subjects to reflect the distribution among the eligible study population seen at the University of Utah Primary Care clinics (which, due to the demographics of Salt Lake City, will be predominately white). Ethnic background/race did not significantly predict treatment participation or outcomes in our prior trials of MORE. It is appropriate to make the study available and accessible to all eligible patients irrespective of their ethnic background and to encourage and facilitate the participation of all eligible patients.

15. STUDY DOCUMENTS

13.1 Retention of Records

The PI will retain study-related documents for at least five years following the completion or discontinuation of the study. If the PI's personal situation is such that such archiving can no longer be ensured, the relevant records will be transferred to a central secure location for storage.

16. PUBLICATION AND DISSEMINATION POLICY

The PI in discussion with the Co-Is will set the rules for publication of the data and authorship in conformity with NIH policies. The clinical outcomes of the trial will be published first in accordance with Specific Aim 1, followed by a series of publications on the mechanistic trial outcomes, in accordance with Specific Aims 2 and 3.

14.1 Dissemination

The findings of this trial have important implications for public health and policy, requiring wide dissemination; additionally, several aspects of this trial's approach and implementation will shape clinical practice and form the basis for dissemination after the trial. Should MORE prove to be efficacious, the PI will provide clinical training in MORE to health care providers (e.g., social workers, psychologists, nurses, and physicians) in community settings.

FINAL SAP

Summary of Changes from SAP Version 1.0 to Version 2.0

In addition to corrections to typographical errors and spelling mistakes, the following changes were made to convert version 1.0 to version 2.0:

1. Comment about the final sample size (N=250, rather than the planned N=260).
2. Addition of details to the description of the EMA analysis.

Mindfulness-Oriented Recovery Enhancement for Opioid Misuse and Chronic Pain in Primary Care: A Randomized Controlled Trial

Statistical Analysis Plan Version 2.0
March 2020

Table of Contents

1. BACKGROUND
2. AIMS
3. STUDY DESIGN
4. ENDPOINTS
   - 4.1 Primary
   - 4.2 Secondary
   - 4.3 Tertiary
5. RANDOMIZATION
   - 5.1 Method of Randomization
   - 5.2 Allocation Concealment
6. SAMPLE SIZE
   - 6.1 Preliminary Data
   - 6.2 Sample Size Determination for Primary Outcome
   - 6.3 Power for Secondary Outcomes
   - 6.4 Power for Tertiary Outcomes
7. INTERIM MONITORING PLAN
   - 7.1 Overview
   - 7.2 Trial Adaptation Guidelines
   - 7.3 Monitoring Inter-practice Variation
   - 7.4 Monitoring of Efficacy and Futility
   - 7.5 Monitoring of Safety
8. ANALYTICAL PLAN
   - 8.1 Overview
   - 8.2 Comparability of Treatment Groups
   - 8.3 Analysis of Primary Outcome
   - 8.4 Analysis of Secondary and Tertiary Outcomes

Title	**Mindfulness-Oriented Recovery Enhancement for Opioid Misuse and Chronic Pain in Primary Care: A Randomized Controlled Trial**
Study Design	The design is a parallel group superiority trial. The unit of randomization is the individual patient.
Study Duration	5 years.
Trial Sites	University of Utah Health – Community Physicians Group.
Objective	Conduct a randomized controlled trial to determine the efficacy of a Mindfulness-Oriented Recovery Enhancement intervention for opioid misuse and chronic pain in primary care.
Number of Subjects	The target sample size is 260 participants enrolled (200 + oversampling by 30% for attrition) to provide 80% probability that a two-sided 95% confidence interval will have a margin (half-width) less than +/- .18 standard deviation for the between-groups difference in the study outcomes.
Main Inclusion Criteria	Age ≥18 with a chronic pain-related diagnosis, reporting current pain ≥3 on a 0-10 scale, currently taking prescription opioids for ≥3 months, and surpassed a validated cutpoint for opioid misuse on the Current Opioid Misuse Measure (COMM).
Intervention	Mindfulness-Oriented Recovery Enhancement (MORE) is a group behavioral intervention that unites mindfulness training, cognitive reappraisal, and positive psychological principles into an integrative intervention strategy targeting mechanisms of pain and opioid misuse.
Duration of Intervention	8 weeks.
Primary Outcome	The primary outcome is opioid misuse measured by the Drug Misuse Index, operationalized as scores on the COMM and triangulated by blinded clinical interview and urine drug screen. The co-primary outcome is chronic pain symptomology, operationalized as pain severity and functional interference scores on the Brief Pain Inventory (BPI).

Primary Analysis	Effects of treatment on the DMI will be analyzed with generalized mixed effects models, and effects on the BPI will be analyzed with mixed effect ANCOVA models, adjusting for baseline levels.
Secondary Outcomes	Opioid dose, psychological distress, and opioid craving.
Interim Analysis	Interim monitoring will focus on patient accrual, baseline comparability of treatment groups, protocol adherence, data completeness and quality, and safety. No interim outcome analysis is planned.

1. BACKGROUND

Prescription opioid misuse among chronic pain patients is an emerging public health threat that is being addressed with heightened urgency at both clinical and policy levels. Though opioid analgesic therapy can manage chronic pain, it may confer with significant health risks, including dependence, overdose, and misuse. Opioid misuse is evidenced by aberrant drug-related behaviors such as unauthorized dose escalation or use of prescribed opioids to self-medicate negative emotions that exacerbate craving.[6] Research on treatments for opioid misuse among chronic pain patients is scant; according to a 2015 NIH-AHRQ systematic review of long-term opioid therapy for chronic pain, "no study evaluated the effectiveness of risk mitigation strategies for improving outcomes related to overdose, addiction, abuse, or misuse."[7] Extant therapies may have limited efficacy because they fail to directly target cognitive-affective neural circuits that govern hedonic regulatory responses elicited by pain-, opioid-, and reward-related stimuli.[8] To address this need, the PI translated mechanistic findings from behavioral neuroscience into an innovative treatment for prescription opioid misuse, called **Mindfulness-Oriented Recovery Enhancement** (MORE). MORE integrates training in mindfulness, reappraisal, and savoring of natural rewards to enhance hedonic regulation and target the risk chain at the point where maladaptive emotion-cognition interactions link chronic pain to opioid misuse. MORE is innovative in that it aims to modify associative learning mechanisms dysregulated by the allostatic effects of opioid misuse on brain reward systems via strengthening **top-down cognitive control to restructure bottom-up reward learning from valuation of drug reward to natural reward** – something that no other behavioral intervention for opioid misuse has been designed to do. Accordingly, this project funded by the National Institute on Drug Abuse (NIDA) is a randomized clinical trial (RCT) of MORE to reduce opioid misuse and chronic pain symptoms among patients receiving long-term opioid therapy in primary care.

2. AIMS

AIM 1. To conduct a RCT of the Mindfulness-Oriented Recovery Enhancement (MORE) intervention for co-occurring aberrant drug-related behaviors and chronic pain in primary care.
We will compare the therapeutic impact of MORE and a conventional supportive group (SG) psychotherapy active control condition on clinical outcomes germane to opioid misuse and chronic pain. **Hypotheses**: Opioid misusing patients assigned to MORE, as compared to SG participants, will evidence decreased opioid misuse and pain (*PRIMARY OUTCOMES*), as well as reduced opioid craving, opioid dosing, and psychological distress (*SECONDARY OUTCOMES*) from pre- to post-treatment through 9-month follow-up.

AIM 2. To test and quantify the degree to which MORE's impact on opioid misuse and pain is mediated by proactive control over emotion-cognition interactions (top-down mechanism).
Hypotheses: The impact of MORE on opioid misuse and pain will be mediated by improvements in: **a)** attentional disengagement from opioid-related cues (reduced attentional bias); **b)** emotion regulation**;** and **c)** cognitive coping (e.g., reinterpretation of pain as innocuous sensory information).

AIM 3. To test and quantify the degree to which MORE's impact on aberrant drug-related behaviors is mediated by restructuring of reward processing (bottom-up mechanism).
Hypotheses: The impact of MORE on aberrant drug-related behaviors will be mediated by restructured reward processing, as indicated by **a)** decreases in psychophysiological indices of opioid cue-reactivity; **b)** increases in responsiveness to natural reward cues; and **c)** a shift in the relative salience of these cues.

3. STUDY DESIGN

A two-arm parallel RCT design with an active control group will minimize internal threats to validity. Mindfulness-Oriented Recovery Enhancement (MORE; n=130) and the Supportive Group Psychotherapy control (SG; n=130) will be equated for time spent in treatment. Assessors will be blind to treatment condition. Target sample sizes were determined via power and precision analyses. The unit of randomization is the individual patient. The co-primary outcomes are opioid misuse and chronic pain symptoms. Participants will complete a clinical assessment battery at pre- and post-treatment, and at 3-, 6-, and 9-month follow-ups. Participants will complete a psychophysiological assessment protocol at pre- and post-treatment. Participants

will complete ecological momentary assessments (EMAs) of symptoms, skill practice, and opioid use during treatment and 1 month afterward up to 3 times/day (morning, noon, and night).

4. ENDPOINTS

The primary, secondary, and tertiary outcome measures are summarized in Table 1.

Event	Screen	Pre -Tx	In Tx	Post-Tx	Follow-Up			
					1 Mo	3 Mo	6 Mo	9 Mo
Screening and Stratification Measures								
Informed Consent	X							
Demographics & Medical History	X							
MINI	X							
Primary and Secondary Outcome Measures								
BPI Pain severity and interference		X		X		X	X	X
DMI: ABC, COMM & Urine screen		X		X		X	X	X
Opioid dosing – TLFB and chartreview		X		X		X	X	X
DASS Psychological distress		X		X		X	X	X
Opioid Craving NRS – EMA (see below)			X			X		
Mediators								
**Self-report mediators:**<br>CSQ Reinterpretation<br>FFMQ Mindfulness<br>CERQ Reappraisal<br>WOS Savoring		X		X		X	X	X
**Other tertiary measures:**<br>PANAS Affect<br>CSQ Pain Catastrophizing<br>MLQ Meaning in Life<br>SHAPS Anhedonia<br>PCL PTSD<br>Self-Transcendence		X		X		X	X	X
**Psychophysiological protocol**		X		X				
Intervention Process Measures and Ecological Momentary Assessments								
TMS State mindfulness (after each session)			X					
Opioid craving NRS - EMA			X			X		
Opioid dosing – EMA			X			X		
Affect NRS - EMA			X			X		
Pain Intensity & Unpleasantness NRS - EMA			X			X		
Therapeutic skill practice			X			X		

4.1 Primary Outcomes

The primary outcome is opioid misuse measured by the Drug Misuse Index, operationalized as scores on the COMM and triangulated by blinded clinical interview and urine drug screen. The co-primary outcome is chronic pain symptomology, operationalized as pain severity and functional interference scores on the Brief Pain Inventory (BPI). Overall success of the intervention will occur if opioid misuse decreases and pain functioning improves, while pain severity does not worsen. For conservatism, we evaluate all outcomes with two-sided tests, and the Bonferroni correction to control for multiple comparisons.

<u>*Primary outcomes (PRE/POST/FOLLOW-UP).*</u> Our pre-specified primary **opioid misuse** outcome in clinicaltrials.gov is a validated composite measure - the Drug Misuse Index (DMI).[11] Because there is no single way to identify patients who misuse opioids,[12] the DMI uses 3 levels of data to triangulate opioid misuse: self-reports on a structured questionnaire (i.e., the COMM); clinical assessment of opioid misuse—the Addiction Behaviors Checklist (ABC)[13]—rated by clinical staff (i.e., psychologists, social workers, and nurses) blinded to treatment assignment; and urine toxicology screens. The COMM consists of 17 items rated on a Likert scale (0=never, 4=very often) regarding how often in the past 30 days patients engaged in opioid misuse-related behaviors (e.g., used opioids in ways other than prescribed). COMM scores ≥9 and ABC scores ≥2 are considered positive. A positive rating from the urine screen is given when subjects test positive for illicit drugs or a non-prescribed opioid. Subjects with positive COMM scores will be given a positive DMI and classified as a misuser. If patients deny engaging in opioid misuse-related behavior (COMM scores <9), then positive ratings on both the urine screen and the ABC are needed for a positive DMI, because urine screens can be inaccurate due to variable drug metabolites, and clinician ratings may unreliable.[14,15] Otherwise, subjects will be given a negative DMI. Multiple studies support the validity of this DMI scoring method.[11,16–18] **Chronic pain symptoms** (pain severity and functional interference) will be assessed with the Brief Pain Inventory (BPI).[19]

4.2 Secondary Outcomes

Opioid craving will be measured with 2 items "How much do you want to take your opioids right now?" and "How strong of an urge do have to take opioids right now?" using 0-10 Numeric Rating Scales (NRS) delivered via EMA. Single item measures of craving have been shown to validly predict opioid misuse[16] and be sensitive to MORE treatment effects.[3] **Reduction in opioid dose** will be assessed by the Timeline Followback Procedure (TLFB) and triangulated by chart review of prescription history by the study coordinator.[20] Opioid dose will be converted to oral morphine-equivalent using standardized equianalgesic conversions. Subjects will also document daily opioid dose via EMA. **Psychological distress** will be measured with the Depression Anxiety Stress Scale-21 (DASS-21), a validated, reliable scale (α=.87-.94).[21]

4.3 Tertiary Outcomes

The tertiary outcomes include psychophysiological measures, self-report mediators, and other EMA ratings of momentary affective state and momentary pain ratings.

5. RANDOMIZATION

5.1 Method of Randomization

An electronic random number generator will randomize participants with simple random assignment in blocks of varying sizes (2 - 4) to preserve unpredictability of allocation, which will be concealed by opaque envelopes.

All participants who meet trial eligibility criteria will be randomized via simple random assignment.
A computerized random number generator will produce the simple random assignment.

5.2 Allocation Concealment

Allocation concealment is an important consideration for randomized trials to control for selection bias. To prevent bias and maintain allocation concealment, participants will not be allocated until the day of the first treatment session. Assessments will be conducted by project staff blinded to group assignment (which remained concealed throughout the study). To maintain blinding, the allocation list will be inaccessible to project staff involved in assessment or treatment, and before each assessment, participants will be reminded to not reveal anything that would disclose their treatment assignment to study staff.

6. SAMPLE SIZE

6.1 Preliminary Data

Sample size considerations are based the effects of MORE versus SG on opioid- and pain-related variables from the PI's NIDA R03 grant (R03DA032517). The PI's R03-funded Stage 2 RCT identified medium to large effect sizes on pain and opioid-related variables over the course of the study (Cohen's *d*=.50 - .84).

6.2 Sample Size Determination

The table indicates the smallest detectable effects for a treatment X time interaction term given different sample sizes, assuming a Type I error rate of .05 and varying correlations between repeated measures, which ranged from .34 -.56 per outcome in the R03. Cohen[26] defined *f*=0.10 and *f*=0.25 as small and medium effects, respectively. Thus our target N of 260 (N=200 + oversampling by 30% for attrition) will allow us to detect small-to-medium effects on continuous outcomes. Based on dichotomous clinical classification of opioid misuse data from the R03 (68% improvement for MORE versus 32% for SG), the proposed sample size will offer outstanding power (>.99) to detect differences of this magnitude on this outcome. (b) *Precision of parameter estimation*. Our target N will allow model parameters to be estimated with a high degree of precision. A per group sample size of N=130 provides 80% probability that a two-sided 95% confidence interval will have a margin (half-width) less than +/- .18 standard deviation. Thus the proposed sample will provide precision more than adequate for estimating small clinical effects with high confidence. (c) *Multivariate models*. Generally, structural equation power calculations for the full information maximum likelihood sensitivity analyses can be based on Root Mean Square Error of Approximation (RMSEA) comparisons of nested models. The proposed sample provides, in a realistic testing scenario, greater than 80% power in a 1 *df* test (26 versus 25) of close fit comparing two models with RMSEA of .06 and .05 at α=.05. These calculations are conservative. Maximum likelihood techniques retain all observations at all times, and efficiently incorporate correlated observations to yield an Effective Sample Size that is quantifiably larger than the number of subjects. With fully specified repeated dimensions, the anticipated correlations will yield an effective sample size that is 40% larger, or roughly N=360.

Table 2. Sample Size Estimates

**Minimally detectable effect sizes (Cohen's *f*) for treatment X time interaction (pre-tx, post-tx, follow-up)**				
Correlation	N = 100	N = 110	N = 120	N = 130
0.25	0.16	0.15	0.14	0.14
0.50	0.13	0.12	0.12	0.11
0.75	0.09	0.09	0.08	0.08

Due to COVID-19 and related research restrictions at the University of Utah, recruitment ended on March 31, 2020, with a total of 250 participants enrolled (10 participants less than the originally anticipated target N).

7. INTERIM MONITORING PLAN

7.1 Overview

Interim monitoring will focus on patient accrual, baseline comparability of treatment groups in terms of demographic and clinical characteristics, protocol adherence, loss to follow-up, data completeness and quality, and safety.

7.2 Monitoring of Safety

Details of the safety and adverse event monitoring plan were provided in the study protocol. This is a minimal risk trial. Adverse and serious adverse events will be collected. Unanticipated problems will also be reported to the Medical Safety Monitor.

8. ANALYTIC PLAN

8.1 Overview

The analysis of the primary and secondary outcomes will be according to the principle of intent-to-treat, i.e., participants will be analyzed according to their original treatment assignment regardless of adherence to protocol. SAS 9.4, MPlus, and R 3.0.2 software will be used for all analyses.

8.2 Comparability of Treatment Groups

Comparability of treatment groups will be assessed by comparing the distribution of baseline characteristics in the two groups using appropriate graphical procedures, summary statistics and multivariate methods. The randomization is designed to produce balance on important covariates and baseline levels of the outcome measures. Nonetheless, some degree of pre-randomization imbalance in study outcomes may occur, and we regress post-randomization observations on baseline scores to adjust for such imbalance.

8.3 Analysis of the Primary Outcomes

Effects of treatment on opioid misuse and chronic pain symptoms will be evaluated using linear and nonlinear mixed models with fixed effects consisting of a time factor and between-subjects treatment factor (MORE vs. SG), and will adjust for randomization imbalance by covarying baseline levels of the outcome. For the continuous pain symptom outcome measured by the Brief Pain Inventory (BPI), the longitudinal trajectory for individual *i* on outcome *j* in treatment group *g* at post-randomization time *t* is modeled, under the full-rank parameterization, as

(1) $y_{ijg,t>1} = a_{ijg} + \beta y_{ijg,t=1} + \mu_{jg,t>1} + \varepsilon_{ijg,t>1}$, $a_{ijg} \sim N(0,\psi_j)$, $\varepsilon_{ijg,t>1} \sim MVN(0,\Theta_j)$,

where

a_{ijg} is a random intercept with zero mean and variance ψ_j ,

β is the fixed regression coefficient of the post measures on pre-randomization baseline (time 1) score $y_{ijg,t=1}$

$\mu_{jg,t>1}$ are the post-randomization treatment arm means at times 2 through 5, conditioned on baseline, equivalent in this parameterization to distinct intercept terms $\mu_{jgt>1} = E(y_{ijg,t>1} \mid a_i, y_{ijg,t=1})$ for each group and time, and $\varepsilon_{ijg,t>1}$ are multivariate normal random error terms assumed to follow either an independent scaled diagonal or AR1 error structure, as determined by comparison of BIC model statistics.

Opioid misuse as measured by the Drug Misuse Index (DMI) is a dichotomous longitudinal variable Y, and we will evaluate treatment impact with a generalized linear mixed model specifying a binary distribution and logit link, with time *t* and group *g* as categorical factors and a random intercept ζ_i :

$$\log(\pi_i / (1-\pi_i)) = \alpha_g + \beta_t + \gamma_{gt} + \zeta_i$$

$$Y_{igt} \mid \zeta_i \sim Bernoulli(\pi_{igt}),\ \zeta_i \sim iid\ N(0,\psi).$$

For the opioid misuse outcome (Drug Misuse Index; DMI), it is not necessary to adjust for baseline differences, as all participants exhibited opioid misuse at baseline.

Our primary interest lies in estimating, for the BPI co-primary pain outcome j, the adjusted main effect, which represents the overall benefit of treatment across all four post-treatment measurement points conditional on baseline:

(2) $\hat{\delta}_j = (1/4)\sum_{t=2}^{t=5} \hat{\mu}_{g=SG,t} - \hat{\mu}_{g=MORE,t}$, a linear combination over the four post-randomization (t>1) adjusted treatment means. The key estimand for binary DMI will be the main effect on the additive logit scale over the four post-randomization occasions.

This estimand is meaningful whether a time-by-treatment arm interaction is present or not, as it represents the expected overall treatment benefit for individuals randomly selected from the two populations. The null hypothesis of no treatment impact is $H_{0j} : \delta_j = 0$, a single numerator degree-of-freedom test, which we evaluate at alpha=.05/3 = .0167 (i.e., Bonferroni adjustment for the three primary variables) under restricted maximum likelihood with Kenward-Roger denominator degrees of freedom. To control for false discovery in the three primary outcomes, we will compare the unadjusted p-values against Bonferroni-adjusted alpha =.05/3 = .0167.

The analysis of primary outcomes achieves unbiased estimates of model parameters when missingness arises from processes that depend on observed covariates (Missing at Random, or MAR). Maximum likelihood

estimation (MLE) procedures under an intent-to-treat philosophy are robust against common patterns of missing data.[27,33] MLE is based on all data observations; no values are deleted or imputed. In our tables and reporting, we refer to the p-values under the standard MAR assumption as p_{MAR}. There is however no definitive test for the MAR assumption. But we investigate sensitivity to missing data and possible other covariates using two other approaches. We extend the scope of the MAR analysis using Full Information (Direct) Maximum Likelihood under broader multivariate patterns of missingness, incorporating simultaneous longitudinal outcomes and key sociodemographic variables as auxiliary variables correlated with the outcome. In simulations, this approach often yields performance equal to or better than achieved by multiple imputation and explicit nonignorable missingness models.[34–36] These analyses will be conducted with the latent variable modeling program MPlus, which incorporates full information maximum likelihood estimation in multivariate models. The global hypothesis test will be examined under three sensitivity variations:

a) Replication of the analysis model (1) under simultaneous inclusion of the three primary outcomes of drug misuse, pain severity, and pain interference, repeated over post-randomization assessments, along with respective baseline predictor values. In this multivariate analysis, pain severity and pain interference are continuous variables, while drug misuse is represented as a dichotomous outcome under a logit link. All three outcomes are free to correlate in the analysis, with the global hypothesis test imposed by nonlinear equality constraints on the net benefits $H_{0j}:\delta_j=0$ for j=1,2,3, a 3 df test.

b) Replication of the 3 df global hypothesis test in (a) with the addition of the continuous log-transformed opioid use secondary outcome, and the continuous Distress secondary outcome as correlated variables.

c) Replication of (b) with the addition of the demographic predictor variables gender and continuous age at baseline.

(d-f) Re-running variations a, b, and c under constrained longitudinal analysis (CLDA) rather than regression adjustment for baseline. Under CLDA, the baseline means are constrained to be equal across arms. These constraints are straightforward under multivariate modeling. The overall benefit contrasts among the post-baseline means, conditional on within-outcome baseline treatment arm equalities, are equivalent to the difference in areas under the curve for the conditional post-baseline mean trajectories.

In the text and tables, we report p-values obtained under Model C as p_{MAR+}, to indicate that the standard MAR analysis has been supplemented with several auxiliary variables, as described above.

Finally, we re-estimated all analyses under a simple selection model[37,38] in which missingness is dependent on the value of the unobserved outcome in as well as the prior observed responses. In the text and table, we report p-values obtained under this model as p_{NMAR}. The Not Missing at Random designation indicates that this model does not require the more restrictive assumptions of MAR, but may be valid when missingness depends on underlying and unobserved values.

8.4 Analysis of the Secondary and Tertiary Outcomes.

Analysis of opioid medication dose will also be implemented as a linear mixed effects model of longitudinal morphine equivalent intake, conditional on baseline, under a log transformation to reduce skew. Distress and other psychological outcomes will similarly be analyzed using linear mixed models adjusted for randomization imbalance by covarying baseline levels of the outcome. Exact unadjusted p-values are reported for the secondary and tertiary outcomes. As in the analysis of our continuous primary outcomes, our primary interest lies in the adjusted main effect of treatment, which with this model specification represents the average effect of treatment across all post-treatment measurement points.

Control for multiple comparisons among the secondary and tertiary variables is provided by a global hypothesis test evaluated at alpha=.05 in the multivariate mixed effects model as $H_{0,\text{joint}}:\forall j,\ \delta_j=0$ versus $H_{1,\text{joint}}: some\ \delta_j \neq 0$ for some *j*. Multivariate contrasts may be implemented in the univariate mixed model

framework of SAS 9.4 by introducing a categorical indicator variable for outcome *j*, interacting this variable with all other effects, and specifying distinct covariance matrices for each *j*. The global net benefit hypothesis is a custom contrast with *J* df, where J is the number of non-primary outcomes. Rejection of the global hypothesis test implies a significant treatment impact on at least one secondary outcome *j*, and we report exact p-values for the univariate tests.

Descriptive analysis of opioid dosing data will examine group differences in the proportion of participants who achieve an opioid dose reduction of ≥50% at each time point.

To explore whether the cognitive, affective, and physiological mediators specified in **Aims 2 and 3** statistically mediate the effect of treatment on outcome variables, we will conduct structural equation path analyses according to established guidelines[28] by evaluating three regression paths: A) *'a'* path between treatment indicator and change in outcome (e.g., opioid misuse) from pre-treatment to follow-up; B) the *'b'* path between pre-post treatment change in the mediator (e.g., attentional bias) and pre-treatment to follow-up change in the outcome (e.g., opioid misuse); and C) the *'c'* path between treatment and pre-post treatment change in the mediator (e.g., attentional bias). We will test mediation by evaluating the significance of the joint product of the *a* and *b* paths, with bootstrapping[29] used to test the significance of the indirect effect. Our team developed a comprehensive analysis of mediation under possible moderation for clinical trials,[30] allowing for the possibility that treatment has changed relationships, not just levels, among variables - this will further guide analyses for Aims 2 and 3. Path models will be corrected for multiple comparisons. Mediational analyses will be published in a subsequent report following publication of the primary trial outcomes.

For EMA, we will use growth curve analysis[31] to examine treatment and homework practice effects on: trajectories of craving (and other tertiary EMA outcomes – e.g., affective state). The basic growth curve model will be specified with fixed effects consisting of continuous time *t* (in days), categorical period *p* (AM, Noon, or PM reporting) and the categorical between-subjects treatment factor (*g* = MORE vs. SG). Random effects will incorporate correlated intercept and period-specific trend components, as in standard growth models:

$$y_{itpg} = b_{0i} + b_{1ipg} t + \varepsilon_{itpg}\ ;\ b_{0i}, b_{1ipg} \sim MVN([\beta_{0p}, \beta_{1pg}]', \mathbf{\Psi}),\ \varepsilon_{itpg} \sim N(0, \sigma^2)\ .$$

The model allows different trends in the three different reporting periods of the day. The key null hypothesis of treatment impact in EMA will be evaluated by testing the equality of the three fixed effect period trends between arms:

$$H_0 : \sum_p \beta_{1p,g=SG} / 3 = \sum_p \beta_{1p,g=MORE} / 3 = \sum_p \beta_{1p} / 3$$

Additional temporal complexity and serial dependence will be considered in polynomial and autoregressive models, as evaluated with the Bayesian Information Criterion. More complicated relationships will be considered in ALT models that include additional cross-lagged and auto-regressive effects.

ORIGINAL SAP

Mindfulness-Oriented Recovery Enhancement for Opioid Misuse and Chronic Pain in Primary Care: A Randomized Controlled Trial

Statistical Analysis Plan Version 1.0
October 2015

Table of Contents

1. BACKGROUND
2. AIMS
3. STUDY DESIGN
4. ENDPOINTS
    - 4.1 Primary
    - 4.2 Secondary
    - 4.3 Tertiary
5. RANDOMIZATION
    - 5.1 Method of Randomization
    - 5.2 Allocation Concealment
6. SAMPLE SIZE
    - 6.1 Preliminary Data
    - 6.2 Sample Size Determination for Primary Outcome
    - 6.3 Power for Secondary Outcomes
    - 6.4 Power for Tertiary Outcomes
7. INTERIM MONITORING PLAN
    - 7.1 Overview
    - 7.2 Trial Adaptation Guidelines
    - 7.3 Monitoring Inter-practice Variation
    - 7.4 Monitoring of Efficacy and Futility
    - 7.5 Monitoring of Safety
8. ANALYTICAL PLAN
    - 8.1 Overview
    - 8.2 Comparability of Treatment Groups
    - 8.3 Analysis of Primary Outcome
    - 8.4 Analysis of Secondary and Tertiary Outcomes

Title	**Mindfulness-Oriented Recovery Enhancement for Opioid Misuse and Chronic Pain in Primary Care: A Randomized Controlled Trial**
Study Design	The design is a parallel group superiority trial. The unit of randomization is the individual patient.
Study Duration	5 years.
Trial Sites	University of Utah Health – Community Physicians Group.
Objective	Conduct a randomized controlled trial to determine the efficacy of a Mindfulness-Oriented Recovery Enhancement intervention for opioid misuse and chronic pain in primary care.
Number of Subjects	The target sample size is 260 participants enrolled (200 + oversampling by 30% for attrition) to provide 80% probability that a two-sided 95% confidence interval will have a margin (half-width) less than +/- .18 standard deviation for the between-groups difference in the study outcomes.
Main Inclusion Criteria	Age ≥18 with a chronic pain-related diagnosis, reporting current pain ≥3 on a 0-10 scale, currently taking prescription opioids for ≥3 months, and surpassed a validated cutpoint for opioid misuse on the Current Opioid Misuse Measure (COMM).
Intervention	Mindfulness-Oriented Recovery Enhancement (MORE) is a group behavioral intervention that unites mindfulness training, cognitive reappraisal, and positive psychological principles into an integrative intervention strategy targeting mechanisms of pain and opioid misuse.
Duration of Intervention	8 weeks.
Primary Outcome	The primary outcome is opioid misuse measured by the Drug Misuse Index, operationalized as scores on the COMM and triangulated by blinded clinical interview and urine drug screen. The co-primary outcome is chronic pain symptomology, operationalized as pain severity and functional interference scores on the Brief Pain Inventory (BPI).

Primary Analysis	Effects of treatment on the DMI will be analyzed with generalized mixed effects models, and effects on the BPI will be analyzed with mixed effect ANCOVA models, adjusting for baseline levels.
Secondary Outcomes	Opioid dose, psychological distress, and opioid craving.
Interim Analysis	Interim monitoring will focus on patient accrual, baseline comparability of treatment groups, protocol adherence, data completeness and quality, and safety. No interim outcome analysis is planned.

1. BACKGROUND

Prescription opioid misuse among chronic pain patients is an emerging public health threat that is being addressed with heightened urgency at both clinical and policy levels. Though opioid analgesic therapy can manage chronic pain, it may confer with significant health risks, including dependence, overdose, and misuse. Opioid misuse is evidenced by aberrant drug-related behaviors such as unauthorized dose escalation or use of prescribed opioids to self-medicate negative emotions that exacerbate craving.[6] Research on treatments for opioid misuse among chronic pain patients is scant; according to a 2015 NIH-AHRQ systematic review of long-term opioid therapy for chronic pain, "no study evaluated the effectiveness of risk mitigation strategies for improving outcomes related to overdose, addiction, abuse, or misuse."[7] Extant therapies may have limited efficacy because they fail to directly target cognitive-affective neural circuits that govern hedonic regulatory responses elicited by pain-, opioid-, and reward-related stimuli.[8] To address this need, the PI translated mechanistic findings from behavioral neuroscience into an innovative treatment for prescription opioid misuse, called **<u>Mindfulness-Oriented Recovery Enhancement</u>** (MORE). MORE integrates training in mindfulness, reappraisal, and savoring of natural rewards to enhance hedonic regulation and target the risk chain at the point where maladaptive emotion-cognition interactions link chronic pain to opioid misuse. MORE is innovative in that it aims to modify associative learning mechanisms dysregulated by the allostatic effects of opioid misuse on brain reward systems via strengthening **top-down cognitive control to restructure bottom-up reward learning from valuation of drug reward to natural reward** – something that no other behavioral intervention for opioid misuse has been designed to do. Accordingly, this project funded by the National Institute on Drug Abuse (NIDA) is a randomized clinical trial (RCT) of MORE to reduce opioid misuse and chronic pain symptoms among patients receiving long-term opioid therapy in primary care.

2. AIMS

AIM 1. To conduct a RCT of the Mindfulness-Oriented Recovery Enhancement (MORE) intervention for co-occurring aberrant drug-related behaviors and chronic pain in primary care.
We will compare the therapeutic impact of MORE and a conventional supportive group (SG) psychotherapy active control condition on clinical outcomes germane to opioid misuse and chronic pain. **<u>Hypotheses</u>**: Opioid misusing patients assigned to MORE, as compared to SG participants, will evidence decreased opioid misuse and pain (*PRIMARY OUTCOMES*), as well as reduced opioid craving, opioid dosing, and psychological distress (*SECONDARY OUTCOMES*) from pre- to post-treatment through 9-month follow-up.

AIM 2. To test and quantify the degree to which MORE's impact on opioid misuse and pain is mediated by proactive control over emotion-cognition interactions (top-down mechanism).
<u>Hypotheses</u>: The impact of MORE on opioid misuse and pain will be mediated by improvements in: **a)** attentional disengagement from opioid-related cues (reduced attentional bias); **b)** emotion regulation**;** and **c)** cognitive coping (e.g., reinterpretation of pain as innocuous sensory information).

AIM 3. To test and quantify the degree to which MORE's impact on aberrant drug-related behaviors is mediated by restructuring of reward processing (bottom-up mechanism).
<u>Hypotheses</u>: The impact of MORE on aberrant drug-related behaviors will be mediated by restructured reward processing, as indicated by **a)** decreases in psychophysiological indices of opioid cue-reactivity; **b)** increases in responsiveness to natural reward cues; and **c)** a shift in the relative salience of these cues.

3. STUDY DESIGN

A two-arm parallel RCT design with an active control group will minimize internal threats to validity. Mindfulness-Oriented Recovery Enhancement (MORE; n=130) and the Supportive Group Psychotherapy control (SG; n=130) will be equated for time spent in treatment. Assessors will be blind to treatment condition. Target sample sizes were determined via power and precision analyses. The unit of randomization is the individual patient. The co-primary outcomes are opioid misuse and chronic pain symptoms. Participants will complete a clinical assessment battery at pre- and post-treatment, and at 3-, 6-, and 9-month follow-ups. Participants will complete a psychophysiological assessment protocol at pre- and post-treatment. Participants

will complete ecological momentary assessments (EMAs) of symptoms, skill practice, and opioid use during treatment and 1 month afterward up to 3 times/day (morning, noon, and night).

4. ENDPOINTS

The primary, secondary, and tertiary outcome measures are summarized in Table 1.

Event	Screen	Pre -Tx	In Tx	Post-Tx	Follow-Up			
					1 Mo	3 Mo	6 Mo	9 Mo
Screening and Stratification Measures								
Informed Consent	X							
Demographics & Medical History	X							
MINI	X							
Primary and Secondary Outcome Measures								
BPI Pain severity and interference		X		X		X	X	X
DMI: ABC, COMM & Urine screen		X		X		X	X	X
Opioid dosing – TLFB and chartreview		X		X		X	X	X
DASS Psychological distress		X		X		X	X	X
Opioid Craving NRS – EMA (see below)			X			X		
Mediators								
**Self-report mediators:**<br>CSQ Reinterpretation<br>FFMQ Mindfulness<br>CERQ Reappraisal<br>WOS Savoring		X		X		X	X	X
**Other tertiary measures:**<br>PANAS Affect<br>CSQ Pain Catastrophizing<br>MLQ Meaning in Life<br>SHAPS Anhedonia<br>PCL PTSD		X		X		X	X	X
**Psychophysiological protocol**		X		X				
Intervention Process Measures and Ecological Momentary Assessments								
TMS State mindfulness (after each session)			X					
Opioid craving NRS - EMA			X			X		
Opioid dosing – EMA			X			X		
Affect NRS - EMA			X			X		
Pain Intensity & Unpleasantness NRS - EMA			X			X		
Therapeutic skill practice			X			X		

4.4 Primary Outcomes

The primary outcome is opioid misuse measured by the Drug Misuse Index, operationalized as scores on the COMM and triangulated by blinded clinical interview and urine drug screen. The co-primary outcome is chronic pain symptomology, operationalized as pain severity and functional interference scores on the Brief Pain Inventory (BPI). Overall success of the intervention will occur if opioid misuse decreases and pain functioning improves, while pain severity does not worsen. For conservatism, we evaluate all outcomes with two-sided tests, and the Bonferroni correction to control for multiple comparisons.

Primary outcomes (PRE/POST/FOLLOW-UP). Our pre-specified primary **opioid misuse** outcome in clinicaltrials.gov is a validated composite measure - the Drug Misuse Index (DMI).[11] Because there is no single way to identify patients who misuse opioids,[12] the DMI uses 3 levels of data to triangulate opioid misuse: self-reports on a structured questionnaire (i.e., the COMM); clinical assessment of opioid misuse—the Addiction Behaviors Checklist (ABC)[13]—rated by clinical staff (i.e., psychologists, social workers, and nurses) blinded to treatment assignment; and urine toxicology screens. The COMM consists of 17 items rated on a Likert scale (0=never, 4=very often) regarding how often in the past 30 days patients engaged in opioid misuse-related behaviors (e.g., used opioids in ways other than prescribed). COMM scores ≥9 and ABC scores ≥2 are considered positive. A positive rating from the urine screen is given when subjects test positive for illicit drugs or a non-prescribed opioid. Subjects with positive COMM scores will be given a positive DMI and classified as a misuser. If patients deny engaging in opioid misuse-related behavior (COMM scores <9), then positive ratings on both the urine screen and the ABC are needed for a positive DMI, because urine screens can be inaccurate due to variable drug metabolites, and clinician ratings may unreliable.[14,15] Otherwise, subjects will be given a negative DMI. Multiple studies support the validity of this DMI scoring method.[11,16–18] **Chronic pain symptoms** (pain severity and functional interference) will be assessed with the Brief Pain Inventory (BPI).[19]

4.5 Secondary Outcomes

Opioid craving will be measured with 2 items "How much do you want to take your opioids right now?" and "How strong of an urge do have to take opioids right now?" using 0-10 Numeric Rating Scales (NRS) delivered via EMA. Single item measures of craving have been shown to validly predict opioid misuse[16] and be sensitive to MORE treatment effects.[3] **Reduction in opioid dose** will be assessed by the Timeline Followback Procedure (TLFB) and triangulated by chart review of prescription history by the study coordinator.[20] Opioid dose will be converted to oral morphine-equivalent using standardized equianalgesic conversions. Subjects will also document daily opioid dose via EMA. **Psychological distress** will be measured with the Depression Anxiety Stress Scale-21 (DASS-21), a validated, reliable scale (α=.87-.94).[21]

4.6 Tertiary Outcomes

The tertiary outcomes include psychophysiological measures, self-report mediators, and other EMA ratings of momentary affective state and momentary pain ratings.

5. RANDOMIZATION

5.3 Method of Randomization

All participants who meet trial eligibility criteria will be randomized via simple random assignment.
A computerized random number generator will produce the simple random assignment.

5.4 Allocation Concealment

Allocation concealment is an important consideration for randomized trials to control for selection bias. To prevent bias and maintain allocation concealment, participants will not be allocated until the day of the first treatment session. Assessments will be conducted by project staff blinded to group assignment (which remained concealed throughout the study). To maintain blinding, the allocation list will be inaccessible to project staff involved in assessment or treatment, and before each assessment, participants will be reminded to not reveal anything that would disclose their treatment assignment to study staff.

6. SAMPLE SIZE

6.2 Preliminary Data

Sample size considerations are based the effects of MORE versus SG on opioid- and pain-related variables from the PI's NIDA R03 grant (R03DA032517). The PI's R03-funded Stage 2 RCT identified medium to large effect sizes on pain and opioid-related variables over the course of the study (Cohen's d=.50 - .84).

6.3 Sample Size Determination

The table indicates the smallest detectable effects for a treatment X time interaction term given different sample sizes, assuming a Type I error rate of .05 and varying correlations between repeated measures, which ranged from .34 -.56 per outcome in the R03. Cohen[26] defined *f*=0.10 and *f*=0.25 as small and medium effects, respectively. Thus our target N of 260 (N=200 + oversampling by 30% for attrition) will allow us to detect small-to-medium effects on continuous outcomes. Based on dichotomous clinical classification of opioid misuse data from the R03 (68% improvement for MORE versus 32% for SG), the proposed sample size will offer outstanding power (>.99) to detect differences of this magnitude on this outcome. (b) *Precision of parameter estimation*. Our target N will allow model parameters to be estimated with a high degree of precision. A per group sample size of N=130 provides 80% probability that a two-sided 95% confidence interval will have a margin (half-width) less than +/- .18 standard deviation. Thus the proposed sample will provide precision more than adequate for estimating small clinical effects with high confidence. (c) *Multivariate models*. Generally, structural equation power calculations for the full information maximum likelihood sensitivity analyses can be based on Root Mean Square Error of Approximation (RMSEA) comparisons of nested models. The proposed sample provides, in a realistic testing scenario, greater than 80% power in a 1 *df* test (26 versus 25) of close fit comparing two models with RMSEA of .06 and .05 at α=.05. These calculations are conservative. Maximum likelihood techniques retain all observations at all times, and efficiently incorporate correlated observations to yield an Effective Sample Size that is quantifiably larger than the number of subjects. With fully specified repeated dimensions, the anticipated correlations will yield an effective sample size that is 40% larger, or roughly N=360.

Table 2. Sample Size Estimates

**Minimally detectable effect sizes (Cohen's *f*) for treatment X time interaction (pre-tx, post-tx, follow-up)**				
Correlation	N = 100	N = 110	N = 120	N = 130
0.25	0.16	0.15	0.14	0.14
0.50	0.13	0.12	0.12	0.11
0.75	0.09	0.09	0.08	0.08

9. INTERIM MONITORING PLAN

9.1 Overview

Interim monitoring will focus on patient accrual, baseline comparability of treatment groups in terms of demographic and clinical characteristics, protocol adherence, loss to follow-up, data completeness and quality, and safety.

9.2 Monitoring of Safety

Details of the safety and adverse event monitoring plan were provided in the study protocol. This is a minimal risk trial. Adverse and serious adverse events will be collected. Unanticipated problems will also be reported to the Medical Safety Monitor.

10. ANALYTIC PLAN

10.1 Overview

The analysis of the primary and secondary outcomes will be according to the principle of intent-to-treat, i.e., participants will be analyzed according to their original treatment assignment regardless of adherence to protocol. SAS 9.4, MPlus, and R 3.0.2 software will be used for all analyses.

10.2 Comparability of Treatment Groups

Comparability of treatment groups will be assessed by comparing the distribution of baseline characteristics in the two groups using appropriate graphical procedures, summary statistics and multivariate methods. The randomization is designed to produce balance on important covariates and baseline levels of the outcome measures. Nonetheless, some degree of pre-randomization imbalance in study outcomes may occur, and we regress post-randomization observations on baseline scores to adjust for such imbalance.

10.3 Analysis of the Primary Outcomes

Effects of treatment on opioid misuse and chronic pain symptoms will be evaluated using linear and nonlinear mixed models with fixed effects consisting of a time factor and between-subjects treatment factor (MORE vs. SG), and will adjust for randomization imbalance by covarying baseline levels of the outcome. For the continuous pain symptom outcome measured by the Brief Pain Inventory (BPI), the longitudinal trajectory for individual *i* on outcome *j* in treatment group *g* at post-randomization time *t* is modeled, under the full-rank parameterization, as

(1) $y_{ijg,t>1} = a_{ijg} + \beta y_{ijg,t=1} + \mu_{jg,t>1} + \varepsilon_{ijg,t>1}$, $a_{ijg} \sim N(0,\psi_j)$, $\varepsilon_{ijg,t>1} \sim MVN(0,\Theta_j)$,

where

a_{ijg} is a random intercept with zero mean and variance ψ_j ,

β is the fixed regression coefficient of the post measures on pre-randomization baseline (time 1) score $y_{ijg,t=1}$

$\mu_{jg,t>1}$ are the post-randomization treatment arm means at times 2 through 5, conditioned on baseline, equivalent in this parameterization to distinct intercept terms $\mu_{jgt>1} = E(y_{ijg,t>1} \mid a_i, y_{ijg,t=1})$ for each group and time, and $\varepsilon_{ijg,t>1}$ are multivariate normal random error terms assumed to follow either an independent scaled diagonal or AR1 error structure, as determined by comparison of BIC model statistics.

Opioid misuse as measured by the Drug Misuse Index (DMI) is a dichotomous longitudinal variable Y, and we will evaluate treatment impact with a generalized linear mixed model specifying a binary distribution and logit link, with time *t* and group *g* as categorical factors and a random intercept ζ_i :

$$\log(\pi_i / (1-\pi_i)) = \alpha_g + \beta_t + \gamma_{gt} + \zeta_i$$

$$Y_{igt} \mid \zeta_i \sim Bernoulli(\pi_{igt}),\ \zeta_i \sim iid\ N(0,\psi).$$

For the opioid misuse outcome (Drug Misuse Index; DMI), it is not necessary to adjust for baseline differences, as all participants exhibited opioid misuse at baseline.

Our primary interest lies in estimating, for each primary outcome j, the adjusted main effect, which represents the overall benefit of treatment across all post-treatment measurement points conditional on baseline:

(2) $\hat{\delta}_j = (1/4)\sum_{t=2}^{t=5} \hat{\mu}_{g=SG,t} - \hat{\mu}_{g=MORE,t}$, a linear combination over the eight post-randomization (t>1) adjusted treatment means. The key estimand for binary DMI will be the main effect on the additive logit scale over the four post-randomization occasions.

This estimand is meaningful whether a time-by-treatment arm interaction is present or not, as it represents the expected overall treatment benefit for individuals randomly selected from the two populations. The null hypothesis of no treatment impact is $H_{0j} : \delta_j = 0$, a single numerator degree-of-freedom test, which we evaluate at alpha=.05/3 = .0167 (i.e., Bonferroni adjustment for the three primary variables) under restricted maximum likelihood with Kenward-Roger denominator degrees of freedom. To control for false discovery in the three primary outcomes, we will compare the unadjusted p-values against Bonferroni-adjusted alpha =.05/3 = .0167.

The analysis of primary outcomes achieves unbiased estimates of model parameters when missingness arises from processes that depend on observed covariates (Missing at Random, or MAR). Maximum likelihood estimation (MLE) procedures under an intent-to-treat philosophy are robust against common patterns of missing data.[27,33] MLE is based on all data observations; no values are deleted or imputed. In our tables and reporting, we refer to the p-values under the standard MAR assumption as p_{MAR}. There is however no definitive test for the MAR assumption. But we investigate sensitivity to missing data and possible other covariates using two other approaches. We extend the scope of the MAR analysis using Full Information (Direct) Maximum Likelihood under broader multivariate patterns of missingness, incorporating simultaneous longitudinal outcomes and key sociodemographic variables as auxiliary variables correlated with the outcome.

In simulations, this approach often yields performance equal to or better than achieved by multiple imputation and explicit nonignorable missingness models.[34–36] These analyses will be conducted with the latent variable modeling program MPlus, which incorporates full information maximum likelihood estimation in multivariate models. The global hypothesis test will be examined under three sensitivity variations:

a) Replication of the analysis model (1) under simultaneous inclusion of the three primary outcomes of drug misuse, pain severity, and pain interference, repeated over post-randomization assessments, along with respective baseline predictor values. In this multivariate analysis, pain severity and pain interference are continuous variables, while drug misuse is represented as a dichotomous outcome under a logit link. All three outcomes are free to correlate in the analysis, with the global hypothesis test imposed by nonlinear equality constraints on the net benefits $H_{0j} : \delta_j = 0$ for j=1,2,3, a 3 df test.

b) Replication of the 3 df global hypothesis test in (a) with the addition of the continuous log-transformed opioid use secondary outcome, and the continuous Distress secondary outcome as correlated variables.

c) Replication of (b) with the addition of the demographic predictor variables gender and continuous age at baseline.

(d-f) Re-running variations a, b, and c under constrained longitudinal analysis (CLDA) rather than regression adjustment for baseline. Under CLDA, the baseline means are constrained to be equal across arms. These constraints are straightforward under multivariate modeling. The overall benefit contrasts among the post-baseline means, conditional on within-outcome baseline treatment arm equalities, are equivalent to the difference in areas under the curve for the conditional post-baseline mean trajectories.

In the text and tables, we report p-values obtained under Model C as p_{MAR+}, to indicate that the standard MAR analysis has been supplemented with several auxiliary variables, as described above.

Finally, we re-estimated all analyses under a simple selection model[37,38] in which missingness is dependent on the value of the unobserved outcome in as well as the prior observed responses. In the text and table, we report p-values obtained under this model as p_{NMAR}. The Not Missing at Random designation indicates that this model does not require the more restrictive assumptions of MAR, but may be valid when missingness depends on underlying and unobserved values.

10.4 Analysis of the Secondary and Tertiary Outcomes.

Analysis of opioid medication dose will also be implemented as a linear mixed effects model of longitudinal morphine equivalent intake, conditional on baseline, under a log transformation to reduce skew. Distress and other psychological outcomes will similarly be analyzed using linear mixed models adjusted for randomization imbalance by covarying baseline levels of the outcome. Exact unadjusted p-values are reported for the secondary and tertiary outcomes. As in the analysis of our continuous primary outcomes, our primary interest lies in the adjusted main effect of treatment, which with this model specification represents the average effect of treatment across all post-treatment measurement points.

Control for multiple comparisons among the secondary and tertiary variables is provided by a global hypothesis test evaluated at alpha=.05 in the multivariate mixed effects model as $H_{0,\text{joint}} : \forall j, \delta_j = 0$ versus $H_{1,\text{joint}} : some\ \delta_j \neq 0$ for some *j*. Multivariate contrasts may be implemented in the univariate mixed model framework of SAS 9.4 by introducing a categorical indicator variable for outcome *j*, interacting this variable with all other effects, and specifying distinct covariance matrices for each *j*. The global net benefit hypothesis is a custom contrast with *J* df, where J is the number of non-primary outcomes. Rejection of the global hypothesis test implies a significant treatment impact on at least one secondary outcome *j*, and we report exact p-values for the univariate tests.

Descriptive analysis of opioid dosing data will examine group differences in the proportion of participants

who achieve an opioid dose reduction of ≥50% at each time point.

To explore whether the cognitive, affective, and physiological mediators specified in **Aims 2 and 3** statistically mediate the effect of treatment on outcome variables, we will conduct structural equation path analyses according to established guidelines[28] by evaluating three regression paths: A) *'a'* path between treatment indicator and change in outcome (e.g., opioid misuse) from pre-treatment to follow-up; B) the *'b'* path between pre-post treatment change in the mediator (e.g., attentional bias) and pre-treatment to follow-up change in the outcome (e.g., opioid misuse); and C) the *'c'* path between treatment and pre-post treatment change in the mediator (e.g., attentional bias). We will test mediation by evaluating the significance of the joint product of the *a* and *b* paths, with bootstrapping[29] used to test the significance of the indirect effect. Our team developed a comprehensive analysis of mediation under possible moderation for clinical trials,[30] allowing for the possibility that treatment has changed relationships, not just levels, among variables - this will further guide analyses for Aims 2 and 3. Path models will be corrected for multiple comparisons. Mediational analyses will be published in a subsequent report following publication of the primary trial outcomes.

For EMA, we will use growth curve analysis[31] to examine treatment and homework practice effects on: trajectories of craving (and other tertiary EMA outcomes – e.g., affective state). The basic growth curve model will be specified with fixed effects consisting of continuous time *t* (in days), categorical period *p* (AM, Noon, or PM reporting) and the categorical between-subjects treatment factor (g = MORE vs. SG). Random effects will incorporate correlated intercept and period-specific trend components, as in standard growth models:

$$y_{itpg} = b_{0i} + b_{1ipg} t + \varepsilon_{itpg} \; ; \; b_{0i}, b_{1ipg} \sim MVN([\beta_{0p}, \beta_{1pg}]', \mathbf{\Psi}) \, , \, \varepsilon_{itpg} \sim N(0, \sigma^2) \, .$$

The model allows different trends in the three different reporting periods of the day. The key null hypothesis of treatment impact in EMA will be evaluated by testing the equality of the three fixed effect period trends between arms:

$$H_0 : \sum_p \beta_{1p,g=SG} / 3 = \sum_p \beta_{1p,g=MORE} / 3 = \sum_p \beta_{1p} / 3$$

Additional temporal complexity and serial dependence will be considered in polynomial and autoregressive models, as evaluated with the Bayesian Information Criterion. More complicated relationships will be considered in ALT models that include additional cross-lagged and auto-regressive effects.

References

1. Onken LS, Carroll KM, Shoham V, Cuthbert BN, Riddle M. Reenvisioning Clinical Science Unifying the Discipline to Improve the Public Health. *Clin Psychol Sci*. 2014;2(1):22-34.

2. Barry DT, Irwin KS, Jones ES, et al. Opioids, Chronic Pain, and Addiction in Primary Care. *J Pain Off J Am Pain Soc*. 2010;11(12):1442-1450. doi:10.1016/j.jpain.2010.04.002

3. Garland EL, Manusov EG, Froeliger B, Kelly A, Williams JM, Howard MO. Mindfulness-oriented recovery enhancement for chronic pain and prescription opioid misuse: Results from an early-stage randomized controlled trial. *J Consult Clin Psychol*. 2014;82(3):448.

4. Garland EL, Froeliger B, Howard MO. Effects of Mindfulness-Oriented Recovery Enhancement on reward responsiveness and opioid cue-reactivity. *Psychopharmacology (Berl)*. 2014;231(16):3229-3238. doi:10.1007/s00213-014-3504-7

5. Garland EL, Froeliger B, Howard MO. Neurophysiological evidence for remediation of reward processing deficits in chronic pain and opioid misuse following treatment with Mindfulness-Oriented Recovery Enhancement: exploratory ERP findings from a pilot RCT. *J Behav Med*. 2014;38(2):327-336. doi:10.1007/s10865-014-9607-0

6. Butler SF, Budman SH, Fernandez KC, et al. Development and validation of the Current Opioid Misuse Measure. *Pain*. 2007;130:144-156.

7. Chou R, Turner JA, Devine EB, et al. The Effectiveness and Risks of Long-Term Opioid Therapy for Chronic Pain: A Systematic Review for a National Institutes of Health Pathways to Prevention Workshop. *Ann Intern Med*. 2015;162(4):276. doi:10.7326/M14-2559

8. Garland EL, Froeliger B, Zeidan F, Partin K, Howard MO. The downward spiral of chronic pain, prescription opioid misuse, and addiction: Cognitive, affective, and neuropsychopharmacologic pathways. *Neurosci Biobehav Rev*. 2013;37(10):2597-2607.

9. Garland EL, Gaylord SA, Boettiger CA, Howard MO. Mindfulness training modifies cognitive, affective, and physiological mechanisms implicated in alcohol dependence: Results from a randomized controlled pilot trial. *J Psychoactive Drugs*. 2010;42:177-192.

10. Dworkin RH, Turk DC, Farrar JT, et al. Core outcome measures for chronic pain clinical trials: IMMPACT recommendations. *Pain*. 2005;113(1):9-19.

11. Jamison RN, Ross EL, Michna E, Chen LQ, Holcomb C, Wasan AD. Substance misuse treatment for high-risk chronic pain patients on opioid therapy: A randomized trial. *PAIN*. 2010;150(3):390-400. doi:10.1016/j.pain.2010.02.033

12. Jamison RN, Edwards RR. Risk factor assessment for problematic use of opioids for chronic pain. *Clin Neuropsychol*. 2013;27(1):60-80.

13. Wu SM, Compton P, Bolus R, et al. The addiction behaviors checklist: validation of a new clinician-based measure of inappropriate opioid use in chronic pain. *J Pain Symptom Manage*. 2006;32(4):342-351.

14. Michna E, Jamison RN, Pham L-D, et al. Urine toxicology screening among chronic pain patients on opioid therapy: frequency and predictability of abnormal findings. *Clin J Pain*. 2007;23(2):173-179. doi:10.1097/AJP.0b013e31802b4f95

15. Nedeljkovic SS, Wasan A, Jamison RN. Assessment of efficacy of long-term opioid therapy in pain patients with substance abuse potential. *Clin J Pain*. 2002;18(4):S39-S51.

16. Wasan AD, Ross EL, Michna E, et al. Craving of Prescription Opioids in Patients With Chronic Pain: A Longitudinal Outcomes Trial. *J Pain*. Published online 2012.

17. Wasan AD, Butler SF, Budman SH, et al. Does report of craving opioid medication predict aberrant drug behavior among chronic pain patients? *Clin J Pain*. 2009;25:193-198.

18. Wasan AD, Michna E, Edwards RR, et al. Psychiatric Comorbidity Is Associated Prospectively with Diminished Opioid Analgesia and Increased Opioid Misuse in Patients with Chronic Low Back Pain: *Anesthesiology*. 2015;123(4):861-872. doi:10.1097/ALN.0000000000000768

19. Cleeland CS. *Brief Pain Inventory–Short Form (BPI–SF)*. Houston, TX; 1994.

20. Fals-Stewart W, O'Farrell TJ, Freitas TT, McFarlin SK, Rutigliano P. The Timeline Followback reports of psychoactive substance use by drug-abusing patients: Psychometric properties. *J Consult Clin Psychol J Consult Clin Psychol*. 2000;68(1):134.

21. Antony MM, Bieling PJ, Cox BJ, Enns MW, Swinson RP. Psychometric properties of the 42-item and 21-item versions of the Depression Anxiety Stress Scales in clinical groups and a community sample. *Psychol Assess*. 1998;10(2):176-181. doi:10.1037/1040-3590.10.2.176

22. Rosenstiel AK, Keefe FJ. The use of coping strategies in chronic low back pain patients: Relationship to patient characteristics and current adjustment. *Pain*. 1983;17:33-44.

23. Baer RA, Smith GT, Hopkins J, Krietemeyer J, Toney L. Using self-report assessment methods to explore facets of mindfulness. *Assessment*. 2006;13:27-45.

24. Jose PE, Lim BT, Bryant FB. Does savoring increase happiness? A daily diary study. *J Posit Psychol*. 2012;7(3):176-187.

25. Lau MA, Bishop SR, Segal ZV, et al. The Toronto Mindfulness Scale: development and validation. *J Clin Psychol*. 2006;62:1445-1467.

26. Cohen J. *Statistical Power Analysis for the Behavioral Sciences*. Routledge Academic; 1988. Accessed April 4, 2013. http://books.google.com.proxy.lib.fsu.edu/books?hl=en&lr=&id=Tl0N2lRAO9oC&oi=fnd&pg=PR11&dq=cohen+1988+effect+size&ots=dp3FRln-Yy&sig=joEEBO258j2pEogz5xn_Wf0iRJQ

27. Donaldson GW, Moinpour CM. Learning to live with missing quality-of-life data in advanced-stage disease trials. *J Clin Oncol Off J Am Soc Clin Oncol*. 2005;23(30):7380-7384. doi:10.1200/JCO.2005.07.022

28. Hayes AF. *Introduction to Mediation, Moderation, and Conditional Process Analysis: A Regression-Based Approach*. Guilford Press; 2013. Accessed September 12, 2015. https://books.google.com/books?hl=en&lr=&id=iWFSpQFh-y4C&oi=fnd&pg=PP1&dq=mediation&ots=1y0_nF3BRV&sig=TJr_FPVNz0ZaXEhNuQPivmrH610

29. Preacher KJ, Hayes AF. Asymptotic and resampling strategies for assessing and comparing indirect effects in multiple mediator models. *Behav Res Methods*. 2008;40(3):879-891.

30. Donaldson GW, Nakamura Y, Moinpour C. Mediators, moderators, and modulators of causal effects in clinical trials—-Dynamically Modified Outcomes (DYNAMO) in health-related quality of life. *Qual Life Res*. 2009;18(2):137-145.

31. Singer J, Willett J. *Applied Longitudinal Data Analysis: Modeling Change and Event Occurrence*. Oxford University Press; 2003.

32. Bollen KA, Curran PJ. Autoregressive latent trajectory (ALT) models a synthesis of two traditions. *Sociol Methods Res*. 2004;32(3):336-383.

33. Schafer JL. *Analysis of Incomplete Multivariate Data*. CRC press; 1997.

34. Allison PD. *Missing Data*. Sage publications; 2001.

35. Beunckens C, Molenberghs G, Kenward MG. Direct likelihood analysis versus simple forms of imputation for missing data in randomized clinical trials. *Clin Trials*. 2005;2(5):379-386.

36. Molenberghs G, Kenward M. *Missing Data in Clinical Studies*. Vol 61. John Wiley & Sons; 2007.

37. Heckman JJ. The common structure of statistical models of truncation, sample selection and limited dependent variables and a simple estimator for such models. In: *Annals of Economic and Social Measurement, Volume 5, Number 4*. NBER; 1976:475-492.

38. Little RJ, Rubin DB. *Statistical Analysis with Missing Data*. Vol 793. John Wiley & Sons; 2019.
