# Supplementary material for: Mindfulness-Oriented Recovery Enhancement vs Supportive Group Therapy for Co-occurring Opioid Misuse and Chronic Pain in Primary Care: A Randomized Clinical Trial
Source: JAMA Intern Med. 2022 Feb 28;182(4):407–17. doi: 10.1001/jamainternmed.2022.0033 (PMC8886485; doi:10.1001/jamainternmed.2022.0033)
Supplement: Supplement 2. — eMethods 1. Additional Intervention, Training, and Fidelity Monitoring Details eMethods 2. Considering Mixed-Effects Models in Which Time Is a Key Component eMethods 3. Additional Details on Outcome Analyses eMethods 4. Missing Data Strategy and Additional Sensitivity Analyses eTable 1. Adverse Events eTable 2. Sample Proportions of Patients Achieving a Minimally Clinically Important Reduction in Chronic Pain Symptoms [file jamainternmed-e220033-s002.pdf]

Supplemental Online Content

Garland EL, Hanley AW, Nakamura Y, et al. Mindfulness-Oriented Recovery Enhancement vs supportive group therapy for co-occurring opioid misuse and chronic pain in primary care: a randomized clinical trial. *JAMA Intern Med*. Published online February 28, 2022. doi:10.1001/jamainternmed.2022.0033

eMethods 1. Additional Intervention, Training, and Fidelity Monitoring Details

eMethods 2. Considering Mixed-Effects Models in Which Time Is a Key Component

eMethods 3. Additional Details on Outcome Analyses

eMethods 4. Missing Data Strategy and Additional Sensitivity Analyses

eTable 1. Adverse Events

eTable 2. Sample Proportions of Patients Achieving a Minimally Clinically Important Reduction in Chronic Pain Symptoms

This supplementary material has been provided by the authors to give readers additional information about their work

© 2022 Garland EL et al. *JAMA Internal Medicine.*

eMethods 1. Additional Intervention, Training, and Fidelity Monitoring Details

To help control for therapist effects, all four study therapists delivered both MORE and supportive psychotherapy. Therapists were Master's level clinical social workers, with an average of 6.4 years of experience providing mindfulness and supportive psychotherapy interventions for people with chronic pain and substance use disorders. Although there is no certification in MORE or supportive psychotherapy, MORE therapists were required to have an established, personal meditation practice. Therapists were trained by the first author, who developed a 13-hour MORE training workshop and support group training protocols for the initial pilot study of MORE (R03DA032517). Training involved didactic and experiential instruction, with the first author monitoring and providing real-time feedback on mock therapist-client interactions at the Center on Mindfulness and Integrative Health Intervention Development (C-MIIND).

MORE sessions focused on applying mindfulness, reappraisal, and savoring skills to promote positive psychological health, produce analgesia, and reduce maladaptive affective and appetitive responses. In MORE, participants were taught foundational mindfulness skills to promote self-awareness, self-regulation, and self-transcendence. As the MORE treatment sequence progressed, mindfulness training synergized reappraisal and savoring techniques not found in other mindfulness-based interventions. To cope with symptoms of pain and craving, patients were first taught unique mindful breathing and body scan meditations designed to decompose experiences of pain and craving into their constituent sensations (e.g., heat, tightness, tingling, vibration), as well as to increase awareness of the center, edges, and permeability (versus solidity) of these sensations, and any adjacent or distal pleasant sensations. Next, patients were taught reappraisal techniques, in which integrated mindfulness and cognitive restructuring skills were used to disengage from negative appraisals and generate adaptive reappraisals to reduce distress and opioid misuse. Finally, patients were taught to savor the pleasant sensory features of naturally rewarding objects and events while cultivating meta-awareness of the positive emotions and pleasurable sensations occasioned by rewarding life experiences. Psychoeducation content of the MORE session topics included the following: 1) discriminating between nociception, pain, and suffering; 2) gaining awareness of automaticity in chronic pain and opioid use; 3) disrupting the link between negative emotions, catastrophizing, and pain experience through reappraisal; 4) savoring pleasant experiences to remediate reward dysregulation; 5) regulating opioid craving through mindful awareness and mindful reappraisal; 6) preventing opioid misuse by disrupting the link between stress and craving; 7) cultivating self-transcendence and meaning in life by connecting with something greater than the self; and 8) developing a mindful recovery plan.

The supportive psychotherapy intervention involved discussion of topics pertinent to chronic pain and opioid misuse that were selected to roughly match corresponding themes in the MORE intervention, including: physical and psychological dimensions of pain; stress and coping; stigma of opioid use and misuse; use of opioids to alleviate negative emotions; and opioid-related adverse effects.. During these supportive psychotherapy discussions, no cognitive-behavioral or mindfulness and acceptance-based skills were discussed or taught. Instead, supportive psychotherapy participants were guided via client-centered reflective listening techniques to disclose feelings and thoughts about group topics, as well as to provide advice and emotional support for their peers. During the supportive psychotherapy intervention, therapists engaged in

an array of general therapeutic behaviors, including building rapport, presenting unconditional positive regard, active listening, empathic responding, elicitation of emotional expression, and promoting mutual support between group members. No specific therapeutic skill training was provided. This control intervention, which typifies a widely-available form of conventional, process-oriented group therapy, was found in three prior RCTs to have equivalent perceived credibility (via the Treatment Credibility Questionnaire, Borkovec & Nau, 1972) to mindfulness-based interventions, including MORE (Garland et al., 2010, 2014; Gaylord et al., 2011). We prevented supportive psychotherapy clinicians from leading or responding to discussions that addressed acceptance and mindfulness by using a manualized supportive psychotherapy protocol and an intensive fidelity monitoring system.

Each treatment session was audio-recorded, and reviewed by a clinical supervisor before the next treatment session, to prevent any treatment diffusion or deviation from the supportive psychotherapy protocol, which was manualized. The validated MORE Fidelity Measure (MORE-FM; Hanley & Garland, 2020) and a parallel supportive psychotherapy fidelity measure were used to assess treatment fidelity. The MORE-FM demonstrated adequate interrater agreement (Adherence subscale ICC = 0.77, Competence subscale ICC = 0.51) and internal consistency (Adherence $\alpha = 0.89$, Competence $\alpha = 0.92$). Initially, the first author reviewed all MORE and supportive psychotherapy treatment session recordings to monitor fidelity and provided 1 hour of clinical supervision a week until a level of adequate or greater therapist competence and adherence was achieved (mean scores > 3 across items scored on a Likert scale of 0 – 6: 17 items for the MORE Fidelity Measure, 11 items for the Supportive Psychotherapy Fidelity Measure). At that time, clinical supervision was provided every few weeks (or more frequently, as needed) by the first author or by a senior therapist who had already achieved adequate fidelity.

To prevent bias from unintentionally impacting fidelity assessment, clinically-trained research staff (e.g., graduate-level social workers and psychologists trained by the first author in MORE and the supportive psychotherapy interventions) then continued to conduct fidelity monitoring by double rating fidelity for a random sample of ≥ two sessions per cohort. Conflicts between raters were discussed, with consensus being reached or being mediated by a third party. One of the fidelity measure items specifically assessed whether the supportive psychotherapy therapist provided any mindfulness-related content during the session. Fidelity ratings for MORE (mean competence=5.1, mean adherence=5.0) and the supportive psychotherapy interventions (mean competence=5.4, mean adherence=4.9) were excellent, indicating that therapists skillfully adhered to each of the manualized protocols with no treatment diffusion. There were zero instances of supportive psychotherapy clinicians leading discussions on mindfulness or acceptance-based skills or concepts. Supportive psychotherapy clinicians were also explicitly trained to divert discussion away from mindfulness-oriented content. On the few instances when participants raised topics related to mindfulness, supportive psychotherapy clinicians diverted the conversation back to the topic of that supportive psychotherapy session.

© 2022 Garland EL et al. *JAMA Internal Medicine.*

eMethods 2. Considering Mixed-Effects Models in Which Time is a Key Component

In this trial, there were 3 primary outcome measures: the DMI, BPI Pain Severity, and BPI Pain Interference Subscales. There were 3 secondary outcome measures: opioid dose, distress, and opioid craving.

Based on recommendations from peer review, we considered a constrained longitudinal analysis (cLDA) as an alternative to ANCOVA framework for achieving baseline adjustment that is highly appropriate for RCTs yet also yields a meaningful Group X Time interaction. cLDA produces similar results to the ANCOVA framework but treats baseline as an observation sampled from a single population (i.e., with baseline population means assumed equal). In our mixed effects context, the cLDA model is:

$$y_{igt} = b_{0i} + \alpha_{t>0} + g\delta_{t>0} + \varepsilon_{igt}\ ,\ b_{0i} \sim N(\beta_0, \psi)\ ,\ \varepsilon_{igt} \sim N(0, \sigma^2)\ ,$$

Where y_{ijt} is an observation on person i in group g (0=Supportive Psychotherapy, 1=MORE) at time t=0 (pre-randomization baseline), 1 (post), 2 (3 months) , 3 (6 months), 4 (9 months),

b_{0i} is the random intercept for person i, having baseline mean β (equal across treatment arms) and normally distributed variance Ψ,

$\alpha_{t>0}$ is the effect, relative to baseline, of supportive psychotherapy control arm post-baseline times 1, 2, 3, and 4

$g\delta_{t>0}$ is the incremental effect provided by MORE (g=1) at post-baseline times 1, 2, 3, and 4

and ε_{igt} is normally distributed random error .

The cLDA model, with its baseline assumption of equal population means, leads to two interpretable hypotheses for treatment impact:

(1) Overall benefit. $H_{benefit} : \delta = 0$, is the single-degree of freedom test that the estimand $\delta = \sum_{t>0} \delta_{gt}$ the overall benefit, is zero. This yields results that are very similar to that provided by ANCOVA adjustment for baseline.
(2) Trajectory equality. $H_{GT} : \delta_{gt>0} = 0$ (i.e., t=1,2,3,4), a four degree-of-freedom test of interaction or lack of parallelism (i.e., the Group X Time interaction). Given the assumption of equal population means at baseline, lack of parallelism implies coincident treatment arm mean profiles. The test of interaction (lack of parallelism) becomes a test of identical profiles.

Naturally neither the interaction nor the baseline adjustment can apply in the case of binary Drug Misuse Index (DMI) of opioid misuse, which by design has no variance at baseline. For DMI, the first outcome point occurs post-randomization. Although cLDA is not possible in this case, inspection of the data indicates that the maximal effect of MORE vs. supportive psychotherapy on the DMI occurred at the 9-month follow-up (OR=2.94, 95% CI 1.19-7.29, p=0.02).

As expected, our estimates and tests of hypothesis (1) are very similar to the results obtained with our baseline-adjusted ANCOVA analytic framework. With four degrees of freedom the Hypothesis (2) Group X Time test is less powerful, but agrees overall with the ANCOVA results.

For pain severity, the Group X Time interaction test yields F=3.17 (p=.014), compared with p=.003 reported in Table 2. For pain interference, the Group X Time interaction test yields F=9.30 (p<.001), agreeing with the p<.001 reported in Table 2. Hypothesis 2 is not defined for DMI, which has no baseline variation, and thus the Group X Time interaction cannot be computed. For the secondary outcomes, the situation is similar. cLDA for Distress yields for the full Group X Time interaction F=3.112, p=.015. For (log) Morphine Equivalent Dose, F=3.33, p=.010. cLDA for EMA of craving yields for the full Group X Time interaction F=10.70 (p=.001). The less powerful Group X Time interaction tests agree with the findings provided by the ANCOVA-style tests.

We also examined the effect of adding a continuous random slope term to the mixed model (retaining the categorical unstructured fixed effects patterns) for our primary outcomes. This model keeps the nonlinear pattern of mean changes while allowing individual differences in the steepness of these profiles. It is thus more flexible than a standard random slopes and random intercepts model, which fits mean linear trends in each group. Unlike the fixed pre-randomization intercept, which is held equal for both groups, the mean fixed effect for slope, or rate-of-change, is allowed to differ across treatment arms.

$$y_{igt} = b_{0i} + b_{1i}t + \alpha_{t>0} + g\delta_{t>0} + \varepsilon_{igt} \text{ , } b_{0i}, b_{1i} \sim MVN([\beta_0, \beta_{1g}]', \Psi) \text{ , } \varepsilon_{igt} \sim N(0, \sigma^2)$$

(The DMI analysis is based on the analogous generalized linear mixed model.) For the pain intensity mixed model and the DMI generalized mixed model, the addition of the time random effect was insignificant. For pain severity, the adjusted main effect was unchanged in magnitude from Table 2, but achieved greater significance (p=.002). From this we conclude that adding a temporal component did not change the interpretation of the primary outcomes.

© 2022 Garland EL et al. *JAMA Internal Medicine.*

eMethods 3. Additional Details on Outcome Analyses

The original analyses included patient as a random effect in all general linear mixed, and generalized linear mixed, models. Adding clinic as a random effect, along with a random effect of patients hierarchically nested within clinics, in mixed effects analysis models did not noticeably change the significance or magnitude of the main effect of treatment on the DMI outcome, and left all estimates essentially unchanged (e.g., the main effect of treatment on the DMI changed from .7211 [p=.0123] to .7021 [p=.0148]). Similarly, adding clinic as a random effect had no effect on BPI outcomes, as clinic variance was indistinguishable from 0 in those multi-level models (i.e., identical to a model with no random effect of clinic). Because results from models with clinic as a random effect were essentially identical to models without the random effect, for statistical parsimony we did not retain clinic in the final models.

© 2022 Garland EL et al. *JAMA Internal Medicine.*

eMethods 4. Missing Data Strategy and Additional Sensitivity Analyses

Our goal for completing a data collection for timepoint was within 30 days of the target timepoint. If participants did not provide data within 2 months of the target, data were considered missing for that timepoint. The table below depicts the mean and median time of actual data acquisition around each planned timepoint.

Timepoint	Post (target goal within 30 days)	3 mo (target goal within 120 days)	6 mo (target goal within 210 days)	9 mo (target goal within 300 days)
Mean	15.5	122.5	214.5	313.1
Median	13.0	116.0	211.0	308

We employed multiple strategies retain participants in the trial, including re-contacting participants multiple times by phone, email, text, and paper mail, attempting to intercept patients at scheduled medical visits, etc. Nonetheless, given the often transient and vulnerable nature of the study sample, with high rates of chronic medical conditions, serious psychiatric disorders, substance use disorders, chronic pain, and poverty, it was difficult to retain all participants in the trial. Also, due to the onset of COVID-19 in the final study year, a number of participants declined to provide follow-up data during the height of the pandemic. The University also had safety regulations in place for several months during the peak of COVID-19 that constrained "non-essential" human subjects research. Although we conducted this trial with an intent-to-treat philosophy and strove to obtain outcome data on all participants regardless of whether or not they received a full dose of the study treatments, outcome data were missing due to patients discontinuing the study and becoming lost to follow-up or declining to provide outcome data.

Data can be not missing at random (NMAR) when the probability of missingness depends on the underlying magnitude of the outcome (such as study dropout due to a disinclination to report opioid misuse, or study dropout due to not responding to study treatments). To ensure that our study findings do not merely reflect the influence of "treatment responders," we conducted and reported NMAR data analyses. We employed a simple NMAR selection model[28] (see Supplement 1 for details and additional references) to incorporate this possible dependence into the likelihood estimation. Results from the NMAR selection models did not differ in statistical significance or magnitude from the primary analyses reported under the 'missing at random' (MAR) assumption.

To handle the missing data produced by study discontinuation, we incorporated multiple statistical approaches for sensitivity analyses. The primary MAR analyses, and their MAR+, and NMAR variations, are based on strong population models. In Kenward's (2013) terminology, their estimands, even those for the NMAR selection model, fall within a *de jure* framework. Convergent results from these analyses provided additional confidence that the effect estimates reported were robust under reasonable variations of the population model. They did not, however, allow for alternative scenarios in which treatment arm dropouts were not predictable from their past history. For a more stringent evaluation of treatment benefit, we now consider a pattern mixture model, within a *de facto* framework, where we assume that treatment dropouts

© 2022 Garland EL et al. *JAMA Internal Medicine.*

from MORE would evolve in a manner similar to the control group after the point of discontinuation (Mallinckrodt et al, 2013). In other words, treatment dropouts would receive no benefit from their previous treatment history, but would instead respond similarly to control group patients with no treatment exposure. This is a lower-bound worst case scenario (assuming a non-harmful treatment) for the treatment's effect that produces overall estimates that are necessarily more conservative than those reported for Table 2. To implement this analysis, we used the control-based multiple imputation strategy with SAS Proc MI and Proc MIanalyze (with 1000 imputations), as detailed in Ratitch et al (2013). These imputations embedded the same SAS Proc Mixed and SAS Proc glimmix models used to analyze the data, but within the multiple imputation pattern mixture context. Using this conservative pattern mixture control group switching modeling approach, the results remain essentially unchanged. Under pattern mixture analysis, the lower-bound estimates for overall MORE benefit are: DMI OR=1.85 [1.02, 3.10], p=0.026; BPI Pain Severity=0.362 (0.158), p=0.022; BPI Pain Interference=0.780 (0.215), p=0.0003. Given the functional equivalence of these results with those from our original pre-specified analytic approach, we have additional confidence in our MAR, MAR+, and NMAR analyses.

Kenward MG (2013). The handling of missing data in clinical trials. Clin Invest 3(3):241-250.

Mallinckrodt CH, Lin Q, Molenberghs M. (2013). A structured framework for assessing sensitivity to missing data assumptions in longitudinal clinical trials. Pharm Stat. 12(1):1-6.

Ratitch B, O'Kelly M, Tosiello R. (2013). Missing data in clinical trials: from clinical assumptions to statistical analysis using pattern mixture models. Pharm Stat 12(6):337-47.

Additional Sensitivity Analyses

We conducted sensitivity analyses including duration of opioid treatment as a covariate. In these models, duration of opioid treatment did not significantly predict any outcome (ps from 0.16 to 0.67). Further, model parameters remained essentially unchanged, with the main effect of treatment (MORE vs. supportive psychotherapy) continuing to prove significant for the DMI, BPI pain severity and interference outcomes.

Because of the study discontinuation rate due to the onset of COVID-19, we conducted an additional sensitivity analysis in the sample of patients prior to the onset of COVID-19 in the final study year. The results of this sensitivity analysis were the same as those of the analysis of the full sample: the main effect of treatment (MORE vs. supportive psychotherapy) on outcomes remained significant for the DMI (OR=1.96, 95% CI [1.06, 3.61], p=0.027), BPI pain severity (-0.42, 95% CI [-0.77, -0.06], p=0.022), and BPI pain interference (BPI pain interference was -1.05, 95% CI [-1.53, -0.57], p<.001).

© 2022 Garland EL et al. *JAMA Internal Medicine.*

eTable 1. Adverse Events

**Overall**	**MORE**	**Supportive Psychotherapy**	**Total**	**%** (*enrolled)
**Body System and Preferred Term**				
**Psychological** (n=4)				
Severe depression/suicidal ideation*	1	1	2	0.8%
Obsessive thoughts*	0	1	1	0.4%
High suicidal ideation*	0	1	1	0.4%
**Renal** (n=1)				
Pyelonephritis	1	0	1	0.4%
**Cardiovascular and Pulmonary** (n=5)				
Ventricular pulmonary embolism	0	1	1	0.4%
Stroke	0	1	1	0.4%
Chest pain w/ edema in hands and feet	1	0	1	0.4%
Viral bronchitis	0	1	1	0.4%
Pneumonia	0	1	1	0.4%
**Neurological** (n=2)				
Seizure	1	1	2	0.8%
Cataplexy	0	1	1	0.4%
**Dermatological** (n=1)				
Allergic reaction	1	0	1	0.4%
**Trauma** (n=2)				
Sexual assault	1	1	2	0.8%
**Drug Overdose** (n=1)				
Heroin overdose*	1	0	1	0.4%
**Total**	7	10	17	6.8%

* Note – these psychological and overdose-related adverse events were deemed to be unrelated to the study interventions by the independent medical monitor, the trial Data Safety and Monitoring Committee (DSMC), and the Institutional Review Board. For instance, the instance of high suicidal ideation occurred in a patient prior to receiving the study treatment, and the heroin overdose occurred in a patient who was using heroin (in addition to prescription opioids) at the time of enrollment into the trial and before receiving the study treatment.

© 2022 Garland EL et al. *JAMA Internal Medicine.*

eTable 2. Sample Proportions of Patients Achieving a Minimally Clinically Important Reduction in Chronic Pain Symptoms

	MORE	Supportive Psychotherapy	p value
	N (%)	N(%)	
**BPI Pain Severity**			
Post-Treatment	48 (49.5%)	30 (30.9%)	.008
3-Month Follow-Up	41 (50.6%)	24 (30.0%)	.008
6-Month Follow-Up	25 (34.2%)	23 (28.0%)	.41
9-Month Follow-Up	35 (50.0%)	22 (29.3%)	.01
**BPI Pain Interference**			
Post-Treatment	41 (42.3%)	24 (24.7%)	.01
3-Month Follow-Up	35 (43.2%)	19 (23.8%)	.009
6-Month Follow-Up	32 (43.8%)	19 (23.2%)	.006
9-Month Follow-Up	41 (58.6%)	19 (25.3%)	<.001

Note: percentages reflect actual observed data without MAR+ or NMAR analyses. * Per the Initiative on Methods, Measurement, and Pain Assessment in Clinical Trials (IMMPACT) recommendations (Dworkin et al., 2008), a minimally clinically important reduction in pain severity and pain interference on the Brief Pain Inventory is a ≥10% decrease from baseline, and a 1 point decrease from baseline, respectively.

The main manuscript presents effect sizes for the overall between-group benefit. Adjusted standardized between-group point estimates of MORE versus supportive psychotherapy at 9-month follow-up for pain severity (0.48) and pain interference (0.76) exceed those observed in the de C Williams et al. (2020) meta-analysis for CBT versus active control conditions at follow-up (0.08 and 0.12 for pain severity and disability, respectively).

© 2022 Garland EL et al. *JAMA Internal Medicine.*
