# Supplementary material for: Mindfulness-Oriented Recovery Enhancement vs Supportive Group Therapy for Co-occurring Opioid Misuse and Chronic Pain in Primary Care: A Randomized Clinical Trial
Source: JAMA Intern Med. 2022 Feb 28;182(4):407–17. doi: 10.1001/jamainternmed.2022.0033 (PMC8886485; doi:10.1001/jamainternmed.2022.0033)
Supplement: Supplement 3. — Data Sharing Statement [file jamainternmed-e220033-s003.pdf]

Data Sharing Statement

Garland. Mindfulness-Oriented Recovery Enhancement vs Supportive Group Therapy for Co-occurring Opioid Misuse and Chronic Pain in Primary Care. *JAMA Intern Med.* Published February 28, 2022. doi:10.1001/jamainternmed.2022.0033

Data
Data available: Yes
Data types: Deidentified participant data (demographics, summary outcome variables)
How to access data: Data will be available upon reasonable request. Email eric.garland@socwk.utah.edu
When available: With publication

Supporting Documents
Document types: None

Additional Information
Who can access the data: Data will be available upon reasonable request.
Types of analyses: Meta-analysis.
Mechanisms of data availability: Data will be available with a signed data access agreement.
